# Nanovaccines with ferroptosis, necroptosis and STING-activation for synergistic immunotherapy

**DOI:** 10.1186/s13046-026-03726-2

**Published:** 2026-05-11

**Authors:** Jia-Rui Du, Mu-Le Tu, Yong-Xu Xia, Yuan-Qiang Lin, Rui Yang, Chang Weng, Yi Liu, Hao Zhang, Hui Wang, Wen-Jie Feng, Deng-Ke Teng

**Affiliations:** 1 Department of Ultrasound, China-Japan Union Hospital of Jilin University, Changchun, 130033 P.R. China; 2 Institute of Translational Medicine, The First Hospital of Jilin University, Changchun, 130021 P. R. China; 3 Department of Ultrasound, The Third Affiliated Clinical Hospital of Changchun University of Chinese Medicine, Changchun, 130117 P. R. China; 4 State Key Laboratory of Supramolecular Structure and Materials, College of Chemistry, Jilin University, Changchun, 130012 P. R. China

**Keywords:** Nanovaccines, Ferroptosis, Necroptosis, STING pathway, Immunogenic cell death, Immunotherapy

## Abstract

Nanovaccines based on immunogenic cell death (ICD) present a promising strategy in tumor immunotherapy, serving as a bridge connecting tumor cell death to the induction of anti-tumor immunity. However, their efficacy is severely limited by the immunologically silent nature of apoptosis-dependent ICD and the immunosuppressive tumor microenvironment (TME). In this work, we designed a metal‒polyphenol network nanovaccine (SRF@FeShik-cGAMP/HA). Upon reaching the tumor tissue through specific targeting, the nanovaccines disassemble within the tumor cells, releasing the Fenton reaction catalyst ferrous iron (Fe^2+^), the system Xc- inhibitor sorafenib (SRF), and necroptosis inducer shikonin. Fe^2+^, SRF, and shikonin collectively promote the accumulation of lipid peroxide and amplify ferroptosis by providing an exogenous iron source, inhibiting glutathione biosynthesis, and boosting reactive oxygen species production, respectively. Meanwhile, shikonin induces necroptosis. The combination of ferroptosis and necroptosis overcomes the limitation of tumor immune silencing and triggers a strong ICD. Simultaneously, the immune adjuvant 2’3’-cyclic-GMP-AMP (cGAMP) activates the STING pathway, reversing the immunosuppressive TME and enhancing the anti-tumor immune response mediated by ICD. The synergistic action of ferroptosis, necroptosis, and the STING pathway produces a cascade of antitumor immune responses and establishes long-term immune memory. This nanovaccine demonstrates promising therapeutic effects in primary, orthotopic, and recurrent hepatocellular carcinoma (HCC) models. Collectively, our findings present a cancer immunotherapy strategy that exploits dual ferroptosis and necroptosis induction to trigger ICD, bypassing the limitations of apoptosis-dependent pathways. This strategy coordinates with the STING pathway to achieve potent antitumor immunity, providing a promising paradigm for HCC treatment, and offering a promising and translatable path for clinical development.

**Graphical abstract:**

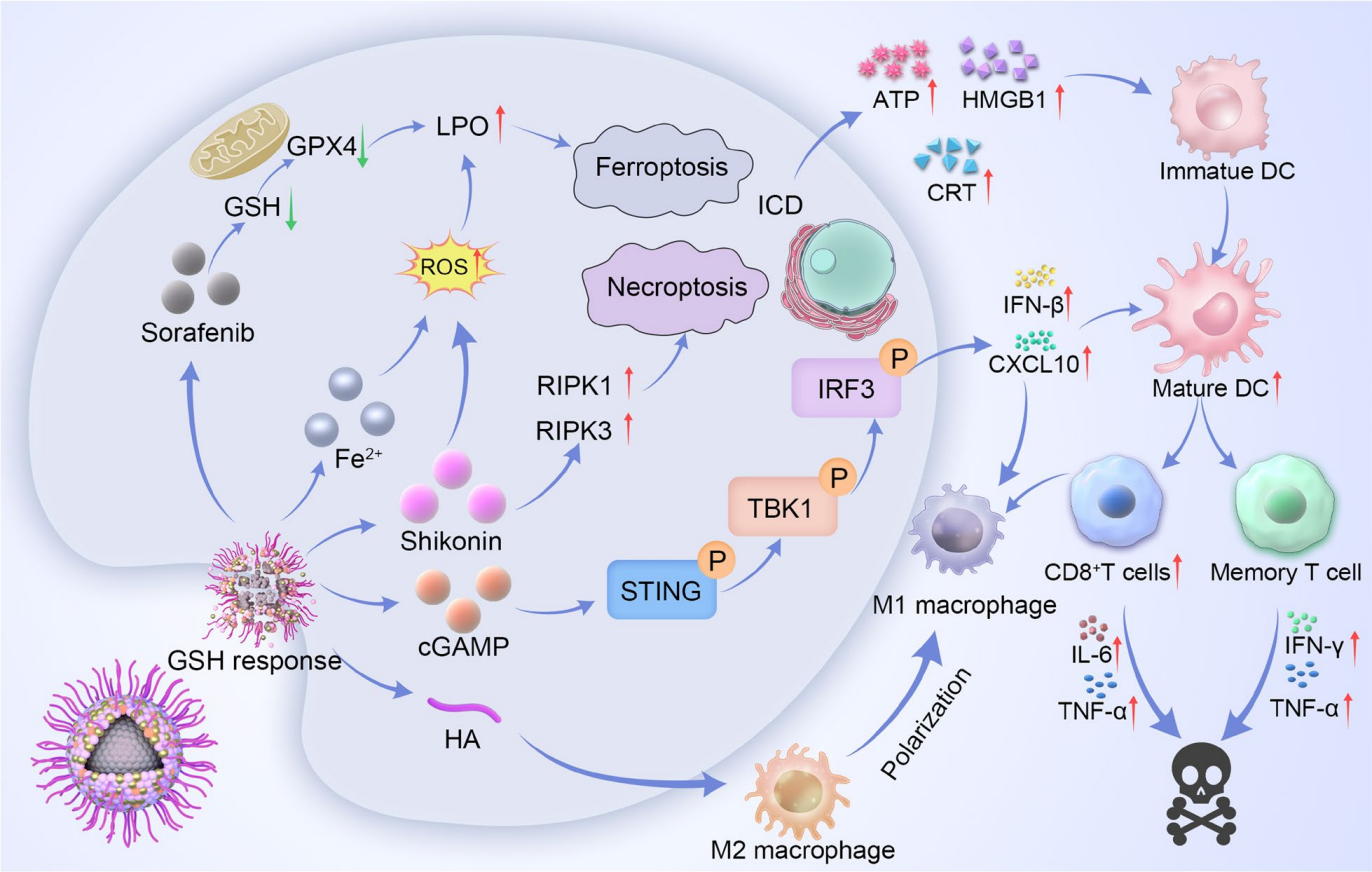

**Supplementary Information:**

The online version contains supplementary material available at 10.1186/s13046-026-03726-2.

## Introduction

With increasing research on tumor therapy, nanovaccines, which can activate the host immune system and destroy tumor cells while preventing tumor recurrence by establishing long-term antitumor immune memory, have been revealed to be a promising immunotherapy strategy [1–3]. However, constructing effective nanovaccines targeting professional antigen-presenting cells (APCs) for tumor immunotherapy remains an enormous challenge [4].

Immunogenic cell death (ICD) is a special type of cell death characterized by the release of tumor-specific antigens and damage-associated molecular patterns (DAMPs). Two key DAMPs are calreticulin (CRT) and high-mobility group protein B1 (HMGB1). CRT functions as a signal that encourages the phagocytosis of dying cells and is involved in enhancing the presentation of antigens. HMGB1 facilitates the stable interaction between APCs and the debris of dying tumor cells. These two DAMPs play a crucial role in the activation and maturation of APCs and the initiation of cytotoxic T lymphocytes (CTLs) [5]. Therefore, inducing ICD within tumors can transform the dead and ruptured tumor cells into vaccines to meet the demands of immune stimulation [6]. However, in most cases, ICD relies on the apoptosis pathway of tumor cells [7–10], and apoptotic cells usually maintain immune silencing [11, 12]. Therefore, exploring ICD-induced enhancement of antitumor immunity through nonapoptotic cell death pathways is necessary.

Ferroptosis is a type of nonapoptotic regulated cell death characterized by iron-mediated lipid peroxides (LPO) and the accumulation of reactive oxygen species (ROS) [13–15]. It triggers the activation of ICD by releasing DAMPs as “find me” signals, thereby increasing immunogenicity [16, 17]. Meanwhile, necroptosis represents a more immunogenic variant of programmed cell death [18], which is facilitated by receptor-interacting protein kinase-1 (RIPK1), RIPK3, along with its substrate executioner protein, mixed lineage kinase domain-like (MLKL) [19, 20]. Necroptosis occurs through membrane permeabilization, leading to the release of intracellular contents, especially immunogenic DAMPs, to induce ICD in tumor cells [21, 22]. Thus, antitumor therapeutic approaches that rely on ferroptosis and necroptosis are expected to overcome the immunosuppression of tumor cells mediated by apoptosis. Moreover, we are convinced that inducing ICD in cancer cells *via* ferroptosis and necroptosis has the potential to enhance antitumor immunity in a synergistic manner.

However, the tumor microenvironment (TME) is a highly immunosuppressive “cold” environment. In this environment, there is insufficient infiltration of immune cells, defective antigen presentation function, metabolic competition and acidosis, all of which will weaken the ICD triggered by ferroptosis and necroptosis in tumor cells [23, 24]. Ferroptosis and necroptosis initiate immune activation by releasing immunogenic signals. However, the immunosuppressive TME limits the durability and efficacy of the anti-tumor immune response initiated by ferroptosis and necroptosis. Therefore, a powerful immune adjuvant is needed to enhance the immunogenicity of ferroptosis and necroptosis and transform the “cold” TME into a “hot” immune inflammatory environment [25]. Among them, 2’3’-cyclic-GMP-AMP (cGAMP) is a stimulatory STING agonist that can directly bind to and activate the STING protein, triggering a series of cascading reactions, leading to the production of type I interferons [26, 27]. Type I interferons can greatly promote the maturation and activation of dendritic cells (DCs), enhance their antigen-presenting capacity, and facilitate the cross-presentation of tumor antigens released by ICD to prime T cells [28]. At the same time, it can also recruit effector immune cells such as CTLs and natural killer (NK) cells to infiltrate the tumor interior, reversing the immunosuppressive TME from “cold” to “hot” [29]. Therefore, ferroptosis and necroptosis initiate ICD in tumor cells, and cGAMP enhances this process by stimulating type I interferon production. This synergistic effect overcomes immunosuppression, amplifies ICD-derived immunogenic signals, and ensures effective immune surveillance and activation.

Herein, we constructed a nanovaccine (SRF@FeShik-cGAMP/HA) that can synergistically activate the STING pathway and trigger dual ferroptosis and necroptosis (Scheme 1). The nanovaccines are prepared by sequentially self-assembling ferric ion (Fe^3+^), shikonin, and sorafenib (SRF), encapsulating cGAMP *via* Fe^3+^-mediated competitive coordination, and subsequently functionalizing the surface with low-molecular-weight hyaluronic acid (HA, MW < 5 kDa). SRF@FeShik-cGAMP/HA can target the CD44 receptor and increase nanovaccines enrichment in the tumor area. After entering tumor cells, the nanovaccines decompose due to the high concentration of glutathione (GSH) in tumors, releasing their components. HA can reverse the immunosuppressive TME, polarize M2-type macrophages into M1-type, and induce the secretion of H_2_O_2_ to provide sufficient H_2_O_2_ in situ for the Fenton reaction. Released Fenton reaction catalyst Fe^2+^, system Xc- inhibitor SRF, and necroptosis inducer shikonin collectively promote the accumulation of LPO and amplify ferroptosis by providing an exogenous iron source, inhibiting GSH biosynthesis, and boosting ROS production, respectively. Meanwhile, the released shikonin induces necroptosis, and dual ferroptosis and necroptosis trigger a strong ICD. Simultaneously, STING agonist cGAMP activates the STING pathway, further enhances the tumor antigen presentation ability. The synergistic effect of ferroptosis, necroptosis, and the STING pathway triggers a domino-like effect, activating multistep cascade antitumor immune responses. This process promotes the maturation and activation of DCs, recruits CTLs and NK cells to infiltrate the tumor interior, reverses the immunosuppressive TME, and establishes long-term immune memory. Our nanovaccines have shown promising anti-tumor therapeutic effects on orthotopic, subcutaneous, and even recurrent hepatocellular carcinoma (HCC) models. This work confirms the immunotherapeutic potential of combining dual ferroptosis, necroptosis, and STING pathway, and is expected to provide new ideas for cancer immunotherapy.

**Sch. 1:**
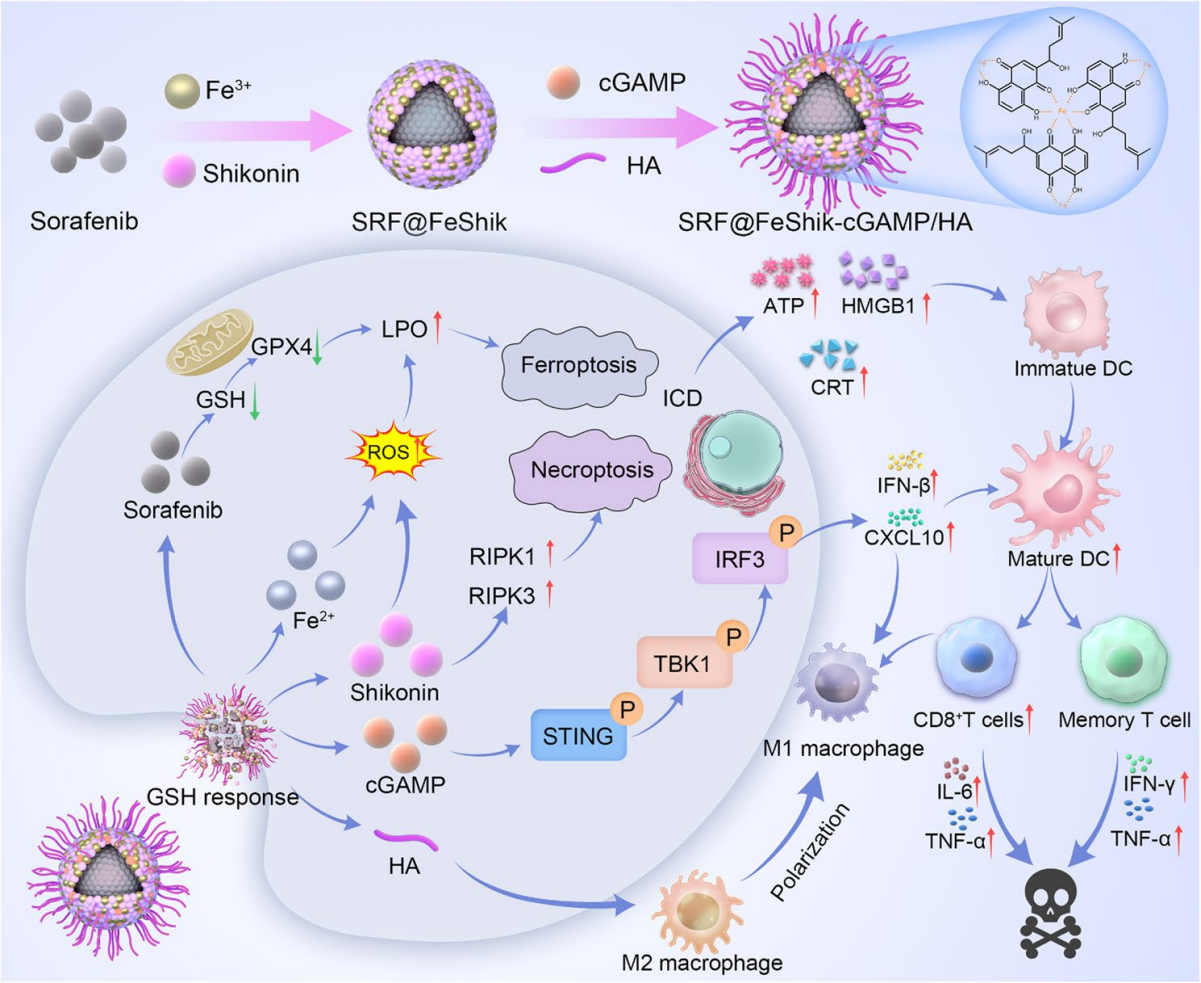
Preparation and mechanism of nanovaccines. Schematic illustration of the preparation of SRF@FeShik-cGAMP/HA nanovaccines and the mechanism of ferroptosis, necroptosis and STING-activation for synergistic immunotherapy

## Materials and methods

### Materials

SRF, iron chloride hexahydrate (FeCl_3_·6H_2_O), urea, sodium chloride (NaCl), disodium ethylenediaminetetraacetate (EDTA), Ortho-phthalaldehyde (OPA), methylene blue (MB), 5-dimethyl-1-pyrroline N-oxide (DMPO), 1,10-phenanthroline, potassium thiocyanate, fluorescein isothiocyanate (FITC), and IR780 were purchased from Aladdin Biochemical Technology Co., Ltd. Shikonin was obtained from Chengdu Gelipu Biotechnology Co., Ltd. cGAMP was purchased from Medbio Medical Technology Co., Ltd. HA (MW = 4.8 kDa) was purchased from Bloomage Freda Biopharm Co., Ltd. Disodium terephthalate (TPA) was purchased from Alfa Aesar. Anti-glutathione peroxidase 4 (GPX4) antibody, anti-RIPK1 antibody, anti-CRT antibody, and anti-HMGB1 antibody were purchased from Abcam. Anti-RIPK3 antibody was purchased from Proteintech. GPX4, RIPK1, RIPK3, tumor necrosis factor α (TNF-α), interferon-γ (IFN-γ), and interleukin-6 (IL-6) enzyme-linked immunosorbent assay (ELISA) kits were purchased from Jiangsu Yutong Biotechnology Co., Ltd. 4’,6-diamidino-2-phenylindole (DAPI), GSH and GSSG assay kit, mitochondrial membrane potential assay kit with JC-1, mitotracker green and 2′,7′-dichlorofluorescein diacetate (DCFH-DA) were purchased from Beyotime Biotechnology. Calcein AM/PI and a cell counting kit-8 (CCK-8) were purchased from Dojindo Molecular Technology. Mouse anti-CD86/PE and mouse anti-CD206/FITC antibodies were purchased from BioLegend Co., Ltd. Phospho-STING and phospho-TBK1 were purchased from Cell Signaling Technology. Phospho-IRF3 was purchased from Affbiotech Co., Ltd.

### Cells and animals

Hepa1-6 mouse liver cancer cells, mouse mononuclear macrophage cell RAW264.7 cells, and human vascular endothelial cells (HUVECs) were provided by the Research Center of China-Japan Union Hospital of Jilin University. These cells were cultured and subcultured in high-glucose DMEM containing 10% fetal bovine serum and 1% penicillin-streptomycin. These cells were cultured at 5% CO_2_ in a constant-temperature incubator at 37 °C. Female C57BL/6J mice (4 weeks, 13–15 g) were purchased from Spiff (Beijing) Biotechnology Co., Ltd.

### Characterization

Transmission electron microscopy (TEM) images were obtained with a JEOL JEM-2100 F. Fourier transform infrared (FTIR) spectra were recorded using a Bruker VERTEX 80v spectrometer. Ultraviolet-visible (UV-vis) absorption spectra were obtained on an ultraviolet‒visible spectrophotometer (Shimadzu, UV2600u). The size of the nanovaccines was measured with a Malvern Zetasizer Nano ZS. Flow cytometry analysis was performed with a flow cytometer (BD FACSCalibur). Confocal laser scanning microscopy (CLSM) was performed using the fluorescence imaging system (Olympus FV1000). Iron concentrations were quantified by inductively coupled plasma mass spectrometry (ICP-MS, Agilent 7700).

### Preparation of SRF@FeShik

The preparation was initiated by dispersing 600 µL of SRF solution (40 mg/mL in DMF) into 120 mL of deionized water under ultrasonication. Subsequently, 1.2 mL of shikonin solution (5 mg/mL in DMF) and 900 µL of FeCl_3_·6H_2_O solution (10 mg/mL) were added sequentially to the mixture. The reaction proceeded under continuous ultrasonication for 1 h. The resulting SRF@FeShik was then isolated by centrifugation (5000 rpm, 15 min) and washed repeatedly with deionized water to remove unreacted precursors.

### Preparation of SRF@FeShik-cGAMP/HA

First, 1.11 mL of SRF@FeShik was dispersed in 18.82 mL of deionized water under stirring. Then, 80 µL of cGAMP (1 mg/mL) was added, and the mixture was stirred for 2 h. Subsequently, 72 µL of HA solution (40 mg/mL) was introduced, followed by continued stirring for 8 h. The reaction mixture was centrifuged at 4500 rpm for 15 min to collect the SRF@FeShik-cGAMP/HA, which was then redispersed in water for further use. 

### *In vitro* responsiveness assay

To evaluate the GSH depletion capability of the nanovaccines, OPA was employed as a fluorescent probe. Nanovaccines at various concentrations (0, 10, 20, 30, 40, 50, 60, and 70 µg/mL) were incubated with a GSH solution. The depletion of GSH was monitored by measuring the change in photoluminescence (PL) intensity using a fluorescence spectrometer (excitation: 350 nm, emission: 420 nm).

To investigate the release profiles of shikonin and Fe^2+^, nanovaccines were dialyzed under continuous agitation in the presence or absence of 10 mM GSH. At specified time intervals, 3 mL of supernatant was withdrawn and substituted with the same volume of fresh medium. Shikonin quantification: the amount of released shikonin was determined by measuring the absorbance at 517 nm using UV‒vis spectroscopy, and the concentration was calculated using a standard calibration curve. Fe^2+^ detection: Fe^2+^ release was assessed using 1,10-phenanthroline as an indicator. The background absorbance (prior to 1,10-phenanthroline addition) was subtracted, and the Fe^2+^ concentration was quantified based on the absorbance at 510 nm. As for the release profiles of SRF, Fe^3+^, and shikonin at pH 7.4, 6.5, and 5.5, the detection method is the same as above. SRF quantification: the amount of released SRF was determined by measuring the absorbance at 268 nm using UV‒vis spectroscopy, and the concentration was calculated using a standard calibration curve. Fe^3+^ detection: Fe^3+^ release was assessed using potassium thiocyanate as an indicator.

### Hydroxyl radical (·OH) generation

A 2 mM GSH solution containing 20 µL of each nanovaccines were first incubated at 37 °C for 2.5 h, followed by the addition of MB and H_2_O_2_ and further incubation at 37 °C for 30 min before UV‒vis spectrophotometric analysis. Parallel experiments were conducted using either TPA as a fluorescent probe for fluorescence spectroscopic quantification (excitation: 310 nm; emission: 425 nm) or DMPO as a spin-trapping agent for electron spin resonance (ESR) spectroscopic analysis, with identical reaction conditions maintained for both methods.

### Cellular uptake and cytotoxicity assay

To investigate the targeting of SRF@FeShik-cGAMP/HA to Hepa1-6 cells, the cells were grown in confocal dishes for 24 h at a density of 1 × 10^5^, FITC-tagged SRF@FeShik-cGAMP and SRF@FeShik-cGAMP/HA were prepared in serum-free DMEM kept in the dark and introduced to the dishes, where they were incubated for intervals of 1, 2, or 3 h. Besides, to verify the targeting specificity, Hepa1-6 cells were precultured with HA for 2 h and then incubated with ^FITC^SRF@FeShik-cGAMP/HA at the same dose for different time. Then, following three washes with PBS, the cells were fixed using 4% paraformaldehyde (PFA) and subsequently stained with Hoechst 33342 for 10 min. Results were captured using CLSM. Similarly, Hepa1-6 cells were seeded into 6-well plates at a density of 2 × 10^5^, and ^FITC^SRF@FeShik-cGAMP, ^FITC^SRF@FeShik-cGAMP/HA, and HA + ^FITC^SRF@FeShik-cGAMP/HA was dispensed into each well, followed by incubation for the same duration, digestion, centrifugation, and resuspension in PBS. The cellular uptake of the nanovaccines was subsequently analyzed *via* flow cytometry.

The cytotoxicity of SRF@FeShik-cGAMP/HA was examined *via* CCK-8 assays. HUVECs and Hepa1-6 cells were cultured in 96-well plates (5 × 10^3^ per well) and incubated 24 h. Thereafter, gradient concentrations of SRF@FeShik-cGAMP/HA (1, 3, 5, 7, 9, 10, and 11 µg/mL) were added to each well for 24 h of incubation. After incubation, remove the culture medium, and measure cell viability through a CCK-8 assay. Live and dead cells were detected using Calcein AM/PI. Hepa1-6 cells were inoculated in confocal dishes and treated with PBS, SRF, FeShik, SRF@FeShik, SRF@FeShik-HA or SRF@FeShik-cGAMP/HA. A proportional addition of Calcein AM and PI dyes was subsequently made, and after 30 min of staining, CLSM was utilized to ascertain the distributions of live and dead cells.

To investigate the mechanism of SRF@FeShik-cGAMP/HA cytotoxicity, ferroptosis inhibitor ferrostatin-1 (Fer-1) (1 µM), necroptosis inhibitor necrostatin-1 (Nec-1) (1 µM), vitamin C (VC) (50 µM), vitamin E (VE) (200 µM), or deferoxamine (DFO) (5 µM) was introduced to Hepa1-6 cells in 96-well plates, following which we evaluated cellular activity using CCK-8 assays to investigate the mechanism of action of the nanovaccines after a 24-hour incubation period.

### Detection of intracellular Fe^2+^, GSH and GSSG

Hepa1-6 cells were cultured in confocal dishes. After incubation with PBS, SRF, FeShik, SRF@FeShik, SRF@FeShik-HA or SRF@FeShik-cGAMP/HA for 24 h, the Hepa1-6 cells were incubated with FerroOrange for 30 min and observed using CLSM. Hepa1-6 cells were inoculated in 6-well cell culture plates and incubated for 24 h, and then treated with PBS, SRF, FeShik, SRF@FeShik, SRF@FeShik-HA or SRF@FeShik-cGAMP/HA. The GSH and GSSG assay kit was used to measure the concentration of GSH and GSSG, while the BCA assay kit was employed to determine the protein concentration. For time-dependent GSH/GSSG ratio analysis, cells were treated with SRF@FeShik-cGAMP/HA for 0, 1, 2, 4, 6, 8, 10, 12, and 24 h, and the experimental procedure was the same as described above.

### Detection of GPX4 and RIPK1/RIPK3 expression

For ferroptosis-related and necroptosis-related ELISA analyses, Hepa1-6 cells were inoculated in 6-well cell culture plates and incubated for 24 h, and then treated with PBS, SRF, FeShik, SRF@FeShik, SRF@FeShik-HA or SRF@FeShik-cGAMP/HA. After treatments, the cells were dissolved using cell lysis buffer. Then, solution after cell lysis was used to detect the RIPK1/RIPK3, and GPX4 content by ELISA kit according to vendors’ instructions.

### Intracellular LPO accumulation assay

The probe utilized for detecting LPO accumulation within the cells was BODIPY^581/591^-C11. Hepa1-6 cells were seeded into confocal dishes and allowed to incubate for 24 h. Following this incubation period, the cells underwent various treatments (PBS, SRF, FeShik, SRF@FeShik, SRF@FeShik-HA or SRF@FeShik-cGAMP/HA) were washed with PBS after another 24 h, and were subsequently stained with the BODIPY^581/591^-C11 reagent for 30 min. The intracellular LPO content was then assessed using CLSM. Malondialdehyde (MDA) assay kit was used to determine the MDA content in the cells. Hepa1-6 cells were inoculated in 6-well cell culture plates and incubated for 24 h, and then treated with PBS, SRF, FeShik, SRF@FeShik, SRF@FeShik-HA or SRF@FeShik-cGAMP/HA. Then, the cells were collected and lysed. The MDA content in the different groups was assessed using an MDA assay kit.

### Detection of intracellular ROS

To assess the production of intracellular ROS following various treatments, we cultured Hepa1-6 cells in confocal dishes and applied treatments with PBS, SRF, FeShik, SRF@FeShik, SRF@FeShik-HA, or SRF@FeShik-cGAMP/HA. After a 24-hour incubation period, the cells were stained with the ROS-indicator DCFH-DA for 30 min, followed by three washes with PBS. The content of intracellular ROS was then evaluated *via* CLSM. Moreover, flow cytometry was employed to further investigate the ROS levels in Hepa1-6 cells post-treatment with nanovaccines. These cells were cultured in 6-well plates and incubated for 24 h. Subsequently, the same treatments were applied for an additional 24 h, after which the cells were digested with trypsin, centrifuged, resuspended, and stained for 30 min. The intracellular ROS levels were quantified using flow cytometry.

### Mitochondrial membrane potential assay

To detect changes in the intracellular mitochondrial membrane potential, we inoculated Hepa1-6 cells in confocal dishes for 24 h of incubation. After treatment (PBS, SRF, FeShik, SRF@FeShik, SRF@FeShik-HA, or SRF@FeShik-cGAMP/HA), the cells were incubated for 24 h, washed with PBS and stained with JC-1 working solution for 20 min. Intracellular fluorescence was observed *via* CLSM. Moreover, flow cytometry was employed to further investigate the mitochondrial depolarization in Hepa1-6 cells post-treatment with nanovaccines. These cells were cultured in 6-well plates and incubated for 24 h. Subsequently, the same treatments were applied for an additional 24 h, after which the cells were digested with trypsin, centrifuged, resuspended, and stained for 30 min. The intracellular red/green ratio were quantified using flow cytometry.

### RNA sequencing and analysis

Hepa1-6 cells were seeded into 6-well plates and subjected to treatment with either PBS or SRF@FeShik-cGAMP/HA. Following a 24-hour culture period, RNA was extracted from the samples using TRIzol, and genomic DNA was eliminated with DNaseI. An RNA sequencing (RNA-seq) transcriptome library was established, and high-throughput sequencing was subsequently performed.

### ICD effects *in vitro*

To examine the ICD effect of SRF@FeShik-cGAMP/HA, exposure of CRT, release of HMGB1, and secretion of ATP were examined in vitro. We inoculated Hepa1-6 cells in confocal dishes and treated the cells with PBS, SRF, FeShik, SRF@FeShik, SRF@FeShik-HA or SRF@FeShik-cGAMP/HA. Subsequently, the cells were treated with 4% PFA for fixation and permeabilized using 0.1% Triton X-100 for 10 min. Following three washes with PBS, the cells underwent blocking and were incubated with either anti-HMGB1/FL594 or anti-CRT/FL594 antibody for 30 min. After the medium was removed, the cells underwent three washes with PBS and were subsequently stained using DAPI for a duration of 10 min. Fluorescence imaging of the cells was performed by CLSM.

The ATP assay kit detects ATP secretion. Hepa1-6 cells were inoculated in 6-well cell culture plates and incubated for 24 h, and treated with PBS, SRF, FeShik, SRF@FeShik, SRF@FeShik-HA or SRF@FeShik-cGAMP/HA. Following the incubation period, the supernatants were gathered, and the content of extracellular ATP was assessed using an ATP assay kit in accordance with the manufacturer’s guidelines.

### STING *in vitro*

Hepa1-6 cells were inoculated in confocal dishes, incubated for 24 h, and then, subjected to different treatments (PBS, SRF, FeShik, SRF@FeShik, SRF@FeShik-HA or SRF@FeShik-cGAMP/HA), washed with PBS after 24 h of incubation, and stained with p-STING for 30 min. After the medium was removed, the cells underwent three washes with PBS and were subsequently stained using DAPI for a duration of 10 min. Fluorescence imaging of the cells was performed by CLSM. The secretion of type I interferon was detected. Hepa1-6 cells were inoculated in 6-well cell culture plates and incubated for 24 h, and then treated with PBS, SRF, FeShik, SRF@FeShik, SRF@FeShik-HA or SRF@FeShik-cGAMP/HA. The supernatant was analyzed for proinflammatory cytokines, specifically interferon-β (IFN-β) and C-X-C motif chemokine 10 (CXCL10), utilizing ELISA kits according to a standard procedure.

### Tumor-associated macrophage polarization and measurement of H_2_O_2_ production

RAW264.7 cells were polarized into M2 macrophages, as detailed in our previous study [30]. To investigate whether SRF@FeShik-cGAMP/HA could polarize M2 macrophages toward the M1 phenotype, M2 macrophages were incubated with SRF@FeShik-cGAMP or SRF@FeShik-cGAMP/HA. Subsequently, the cells underwent washing with PBS and were exposed to CD86/PE and CD206/FITC for a period of 30 min. Ultimately, the proportion of M1 to M2 macrophages was assessed using flow cytometry.

Quantification of H_2_O_2_ generation by M1 macrophages. The quantity of H_2_O_2_ generated by the macrophages was assessed utilizing a H_2_O_2_ assay kit. M2 macrophages were treated with SRF@FeShik, SRF@FeShik-cGAMP or SRF@FeShik-cGAMP/HA. After incubation, follow the instructions of the kit for detection and measure the absorbance using an enzyme label instrument. The H_2_O_2_ levels for each group were determined based on the standard curve.

### *In vitro* DC maturation

In accordance with our previous research [31], mouse bone marrow-derived dendritic cells (BMDCs) were isolated from C57BL/6J mice. For the study of DC maturation, Hepa1-6 cells were inoculated in 6-well plates overnight. The cells were then treated with PBS, SRF, FeShik, SRF@FeShik, SRF@FeShik-HA or SRF@FeShik-cGAMP/HA for 24 h. Subsequently, the BMDCs were co-incubated with the Hepa1-6 tumor cells. The cells were subsequently harvested and stained with anti-CD80-PE, anti-CD86-PE-Cy5, and anti-CD11c-FITC antibodies. After washing with PBS, the fluorescence signals were acquired by flow cytometry. ELISA was used to measure the levels of TNF-α and IL-6.

### *In vivo* biodistribution and pharmacokinetic evaluation

Hepa1-6 cells (1 × 10^6^) were subcutaneously inoculated into C57BL/6J mice (female, 4 weeks) to establish tumor model. ^IR780^SRF@FeShik-cGAMP and ^IR780^SRF@FeShik-cGAMP/HA (10 mg/kg) were intravenously injected into the tumor-bearing mice. Fluorescence imaging was performed in vivo at different time points (0, 4, 8, 12, 24, 36, and 48 h). After being sacrificed at 48 h point, the organs and tumors were collected.

For the pharmacokinetic assay, six female C57BL/6J mice were injected intravenously with ^IR780^SRF@FeShik-cGAMP or ^IR780^SRF@FeShik-cGAMP/HA. Blood was collected into an anticoagulant tube from the orbital venous plexus of each mouse by a capillary pipette at different timepoints. The fluorescence intensity related to IR780 was then assessed using a two-dimensional InGaAs array (Princeton Instruments, NIRvana-640) at a laser wavelength of 808 nm. The same method was used to determine the Fe content in heart, liver, spleen, lungs, kidneys, tumor tissue and plasma samples by ICP-MS.

### *In vivo* antitumor effect and antitumor immune mechanism study

Establish the tumor-bearing mice model according to the above method. Upon reaching a tumor volume of 100 mm^3^, the Hepa1-6 tumor-bearing mice were randomly allocated into six distinct groups, with five parallel samples established for each group: (I) Control, (II) SRF, (III) FeShik, (IV) SRF@FeShik, (V) SRF@FeShik-HA, and (VI) SRF@FeShik-cGAMP/HA (10 mg/kg). Mouse body weights and tumor volumes were recorded every 2 days. Following the treatment, tumors from each group were photographed, enabling a comparison of the antitumor effects across the different groups. Additionally, tissues such as the heart, liver, spleen, lungs, and kidneys were collected for hematoxylin-eosin (H&E) staining. After various treatments for 7 days, the tumors were collected for H&E staining, fluorescence staining, and immunofluorescence analysis of necroptosis (RIPK1, RIPK3), ferroptosis (GPX4), ROS (DHE), LPO (BODIPY^581/591^-C11), and ICD (CRT, HMGB1). Moreover, a survival analysis was performed, and the mice were monitored for 60 days. The mice were humanely euthanized when their tumor volume increased to 1500 mm^3^.

To assess the impact of SRF@FeShik-cGAMP/HA on reprogramming the TME, tumors were harvested three days following the administration of various treatments. The presence of M1 and M2 macrophages in the tumors post-treatment was evaluated using immunofluorescence and flow cytometry, following established protocols. For flow cytometry, the cells underwent staining with antibodies against anti-CD11b-APC, anti-F4/80-FITC, anti-CD86-PE-Cy5, and anti-CD206-PE. In the case of immunofluorescence, anti-CD86-PE and anti-CD206-FITC antibodies were utilized to visualize the localization of M1 and M2 macrophages within tumor tissues, respectively.

To determine the effects of SRF@FeShik-cGAMP/HA on the STING pathway, tumors were collected 7 days after various treatments were administered. Subsequently, immunofluorescence staining for p-STING, p-TBK1, and p-IRF3 was performed, and mouse serum was collected for the measurement of CXCL10 and IFN-β levels *via* ELISA.

To analyze the maturation of DCs, spleen samples were collected 14 days after various treatments were administered. Spleens were processed to create a single-cell suspension. Subsequently, these cells were stained with anti-CD11c-FITC, anti-CD80-PE, and anti-CD86-PE-Cy5 antibodies for a duration of 30 min. The cells were then collected *via* centrifugation for flow cytometric analysis. Mouse serum was collected for the measurement of TNF-α and IL-6 levels *via* ELISA.

To analyze the infiltration of NK cells, the tumors were collected 5 days after various treatments. The cells were blocked with anti-mouse CD16/CD32 antibody, stained with anti-CD3-FITC, anti-CD45-PE, and anti-NK1.1-APC antibodies for 30 min and then analyzed using flow cytometry.

For CTLs infiltration analysis, the tumors were collected 14 days after various treatments. The cells were stained with anti-CD3-FITC, anti-CD4-PE, and anti-CD8a-PE-Cy5 antibodies for 30 min and then analyzed using flow cytometry. To examine regulatory T cells (Tregs), the cells were blocked with anti-mouse CD16/CD32 antibody to reduce nonspecific Fc receptor binding. Then, the cells were stained with anti-CD25-FITC, anti-CD4-PE, and anti-Foxp3-PE-Cy5 antibodies for 30 min and analyzed using flow cytometry. For the immunofluorescence analysis, anti-CD8a-PE or anti-Foxp3-PE antibody was used to determine the distribution of CD8 and Treg cells in tumor tissues, respectively.

### Long-term immune effects *in vivo*

A Hepa1-6 tumor-bearing mice model was constructed. SRF@FeShik-cGAMP/HA was injected into the mice on days 0, 3 and 6. On day 28, Hepa1-6 cells were re-implanted into the opposite side of the tumor in the mice. Three days later, serum samples were obtained and diluted for further examination. The levels of TNF-α and IFN-γ were quantified using ELISA. To evaluate immune memory effects, spleens from the mice were collected and the cells were stained with anti-CD3-FITC, anti-CD8-PE-Cy5, anti-CD44-PE, and anti-CD62L-APC antibodies for 30 min, followed by flow cytometry analysis. Tumor volumes were monitored every two days over a period of 50 days to assess the recurrence of antitumor effects.

### Ultrasound visualization of the antitumor effect on orthotopic HCC model

An orthotopic HCC model was constructed using Hepa1-6 cells for real-time ultrasound visualization to observe the in vivo antitumor effect of SRF@FeShik-cGAMP/HA. The specific steps were as follows: The mice were anesthetized and fixed in a supine position on the surgical board. The fur was shaved and disinfected. A longitudinal incision of approximately 1–1.5 cm was made on the midline of the upper abdomen using fine scissors. The skin and peritoneum were gently separated layer by layer, and the abdominal cavity was opened. Use sterile cotton swabs to gently pull out the left or middle lobe of the liver from the abdominal cavity, and place it on a moist saline gauze to keep it moist. Injecting the Hepa1-6 cells suspension (1 × 10^6^, 20 µL), a transparent vesicle could be observed forming beneath the liver capsule. After the injection was completed, gently restore the liver to its position within the abdominal cavity and suture the incision. Seven days after the orthotopic injection of Hepa1-6 cells, the mice were randomly divided into 2 groups and injected with PBS or SRF@FeShik-cGAMP/HA (10 mg/kg). After 0, 3, 6, 9 and 12 days, the volume of the tumors in the liver of the mice was monitored by ultrasound and photographed.

### *In vivo* safety evaluation

Female C57BL/6J mice were randomly divided into four groups, three were injected with SRF@FeShik-cGAMP/HA (10 mg/kg) *via* the tail vein. The control group received an equivalent volume of PBS as the other group. Blood samples were taken from the mice after they were sacrificed at intervals of 1, 7, and 30 days. A biochemical analysis of the blood was conducted, and major organs such as the heart, liver, spleen, lungs, and kidneys were harvested for H&E and fluorescence staining assays.

### Statistical analysis

Experimental data were expressed in this manuscript as the mean ± standard deviation. Comparisons between different groups were analyzed by ANOVA (Analysis of Variance). Values of *P < 0.05, **P < 0.01 and ***P < 0.001 were considered to indicate statistical significance.

## Results and discussion

### Preparation and characterization of SRF@FeShik-cGAMP/HA

**Fig. 1:**
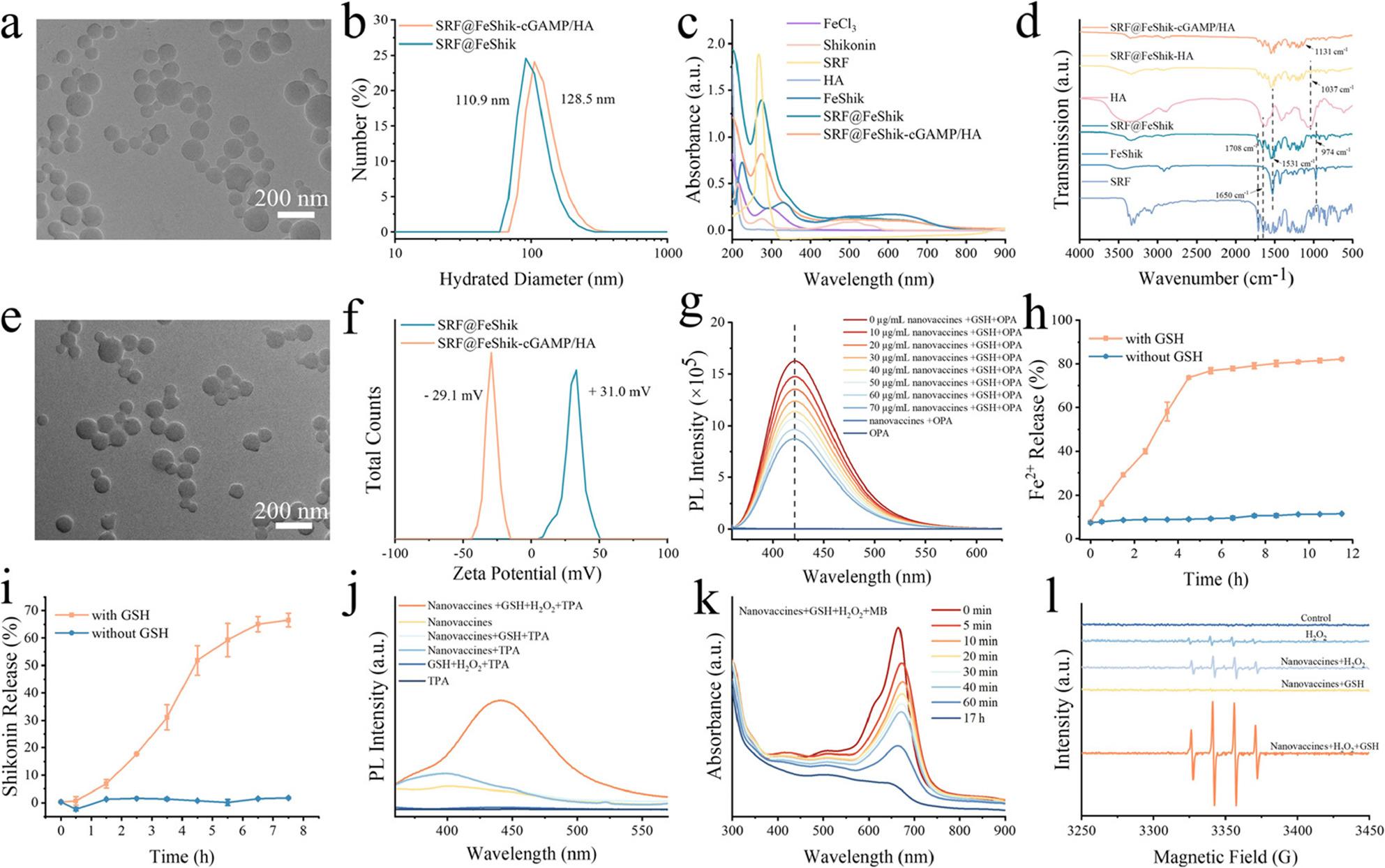
Characterization of SRF@FeShik-cGAMP/HA nanovaccines. **a** TEM image of SRF@FeShik. **b** Hydrated diameters of SRF@FeShik, and SRF@FeShik-cGAMP/HA. **c** UV-vis absorption spectra of FeCl_3_, shikonin, SRF, HA, FeShik, SRF@FeShik, and SRF@FeShik-cGAMP/HA. **d** FTIR spectra of SRF, FeShik, SRF@FeShik, HA, SRF@FeShik-HA, and SRF@FeShik-cGAMP/HA. **e** TEM image of SRF@FeShik-cGAMP/HA. **f** Zeta potential of SRF@FeShik, and SRF@FeShik-cGAMP/HA. **g** Evaluation of GSH depletion capacity in nanovaccines using OPA fluorescence assay. **h** Accumulative Fe^2+^ release of nanovaccines with or without GSH (*n* = 3). **i** Accumulative shikonin release of nanovaccines with or without GSH (*n* = 3). ·OH production evaluation *via* detecting the fluorescence spectra of TPA (**j**), the UV-vis absorption spectra of MB (**k**), and ESR spectra of DMPO (**l**)

As mentioned in the Materials and Methods section, SRF@FeShik is prepared through the self-assembly of Fe^3+^, shikonin, and SRF. The adhesion of FeShik on the SRF surface suppresses its crystal growth caused by Ostwald ripening, confines the particle size to the nanoscale and enhances aqueous dispersibility. The resulting SRF@FeShik are spherical nanoparticles with an average diameter of 101.7 nm (Figs. 1a and S1). Dynamic light scattering (DLS) reveals a hydrodynamic diameter of 110.9 nm (Fig. 1b). The absorption peak in the UV-vis spectrum between 450 and 800 nm is attributed to the charge transfer transition induced by the coordination process between the Fe^3+^ and shikonin (Fig. 1c). The absorption peaks at 268 nm belong to SRF. The FTIR peaks at 1531 and 974 cm^− 1^ verify FeShik formation (Fig. 1d). The peak at 1531 cm^− 1^ corresponds to the C = O stretching vibration bond that has shifted upon the coordination between the Fe^3+^ and shikonin. The peak at 974 cm^− 1^ is characteristic of the Fe-O coordination bond. In addition, the peaks at 1708 and 1650 cm^− 1^ are attributed to the C = O stretching vibrations of the urea group and amide group, respectively, in SRF. These results confirm the successful preparation of SRF@FeShik.

cGAMP is subsequently incorporated by a competitive coordination strategy between SRF@FeShik and cGAMP. Additionally, the nanovaccines surface is functionalized with low-molecular-weight HA. The prepared SRF@FeShik-cGAMP/HA retains its nanospherical structure, with an average diameter of 104.4 nm (Figs. 1e and S2). The hydrodynamic diameter increases to 128.5 nm, and the surface potential shifts from + 31.0 mV to − 29.1 mV (Fig. 1b and f). The peak in the FTIR spectrum at 1037 cm^− 1^ is attributed to the stretching vibration of the aliphatic ether C-O-C bond in HA, whereas the peak at 1131 cm^− 1^ corresponds to the in-plane bending vibration of the primary amine N-H in the cGAMP adjuvant (Fig. 1d). These results confirm the presence of cGAMP and HA. Furthermore, the hydrodynamic diameter, polydispersity index (PDI), and zeta potential of SRF@FeShik and SRF@FeShik-cGAMP/HA undergo negligible changes when stored in H_2_O, PBS (pH 7.4) or serum-containing media over a 7-day period, highlighting the robust colloidal stability of our nanovaccines (Fig. S3).

To investigate the forces driving assembly, SRF@FeShik-cGAMP/HA is treated with various interaction-disrupting reagents (Fig. S4). Precipitation occurs in coordination interaction disruptor disodium EDTA and electrostatic interaction disruptor sodium NaCl solutions, accompanied by gradual reddening in the supernatant after EDTA treatment, indicating structural disassembly of the nanovaccines and the release of shikonin. Treatment of the colloidal solution with the hydrophobic interaction disruptor Triton X-100 exhibits significant alterations, with TEM images confirming complete loss of the nanomorphology. Partial destruction of the nanoparticle morphology is also observed in the π‒π interaction disruptor DMSO group, which includes nonspherical nanoparticles. Further characterization by DLS and UV‒vis spectroscopy reveals markedly increased particle sizes in the NaCl and EDTA solutions, whereas reduced sizes are detected in the Triton X‒100 solution, which is consistent with the observed changes in the state of the solution. The UV‒vis spectrum reveals the disappearance of characteristic peaks in these three solutions, further confirming nanovaccines disassembly. Collectively, these results demonstrate that the primary driving forces for SRF@FeShik-cGAMP/HA formation include coordination, electrostatic, and hydrophobic interactions, with possible contributions from π‒π stacking interactions.

### GSH depletion and ·OH production evaluation

Given that the tumor cells exhibit significantly elevated intracellular GSH concentrations (1–10 mM), the response of GSH to the nanovaccines is investigated. OPA, which can react with GSH to form a benzothiazole derivative that emits bright blue fluorescence at 420 nm, is employed as an indicator to assess GSH depletion by measuring fluorescence spectra. Neither OPA alone nor the mixture of nanovaccines with GSH exhibits any fluorescence signal, but their combination results in a distinct fluorescence signal (Fig. 1g). The intensity at 420 nm decreases progressively as the nanovaccines concentration increases, confirming GSH depletion. To further assess the GSH-induced disassembly of the nanovaccines, TEM images at different time points are obtained, revealing GSH-triggered nanovaccines disassembly, with complete disintegration observed after 12 h (Fig. S5). Furthermore, analysis of the disassembly products is performed using the Fe^2+^ indicator 1,10-phenanthroline. A standard curve is established *via* UV‒vis spectroscopy to quantify Fe^2+^ release (Fig. S6). The results reveal sustained Fe^2+^ release under GSH stimulation, reaching a plateau of ~ 80% at 5 h, whereas negligible Fe^2+^ release is detected without GSH (Fig. 1h). Similarly, shikonin release reaches ~ 70% after 7 h of GSH incubation (Figs. 1i and S7). These findings confirm the response of nanovaccines to the GSH, which enables efficient GSH depletion from the TME and the subsequent release of Fe^2+^ and shikonin.

Fe^2+^ exhibits significantly greater catalytic activity than Fe^3+^ in the Fenton reaction, converting H_2_O_2_ into highly reactive ·OH. TPA is employed as a fluorescent probe for ·OH detection, as it reacts with ·OH to form a strongly fluorescent product. ·OH generation is confirmed by fluorescence spectroscopy only in the presence of the nanovaccines, GSH, and H_2_O_2_ (Fig. 1j). Additionally, these observations are supported by the results of MB decolorization assays. The characteristic absorption peak of MB disappears exclusively in the group containing the nanovaccines, GSH and H_2_O_2_ (Fig. S8), with a time-dependent decline in absorbance (Fig. 1k). ·OH production is further validated by ESR spectroscopy using DMPO as a trapping agent. The characteristic 1:2:2:1 signal confirms the generation of ·OH, which is minimal with the nanovaccines alone but becomes prominent upon GSH-induced disassembly (Fig. 1l). These results demonstrate that the responsiveness of nanovaccines to GSH leads to GSH depletion, Fe^2+^ and shikonin release, and subsequent ·OH generation *via* the Fenton reaction.

Subsequently, the effect of pH on the stability of our nanovaccines is also characterized. The separate release profiles of SRF, Fe^3+^, and shikonin are investigated at pH 7.4, 6.5, and 5.5 in the presence of serum (Figs. S9-S11). Negligible release is observed at all tested pH values within 48 h, indicating that our nanovaccines are stable under extracellular physiological conditions and do not cause premature payload leakage during blood circulation. This pH-insensitive property is consistent with our previous study [32], as the SRF@FeShik nanovaccine is fabricated under acidic conditions (pH ~ 2.6), conferring high stability against extracellular pH changes. In contrast, remarkable and sustained release is triggered by high concentrations of GSH, which are characteristically abundant inside tumor cells, suggesting that our nanovaccines could be selectively activated after internalization into tumor cells rather than responding to extracellular pH changes in the TME.

### Selective cytotoxicity

The cytotoxicity of SRF@FeShik-cGAMP/HA toward Hepa1-6 liver cancer cells (tumor cells) and HUVECs (normal cells) is evaluated by a CCK-8 assay. The viability of Hepa1-6 cells decreases rapidly with increasing concentrations of SRF@FeShik-cGAMP/HA. Only 21.9% of the Hepa1-6 cells survive after incubation with 11 µg/mL SRF@FeShik-cGAMP/HA (Fig. S12a). In contrast, the viability of HUVECs remains as high as 77.1% under the same conditions (Fig. S12b). This suggested the outstanding safety profile of our nanovaccines on normal cells. The selective cytotoxicity of SRF@FeShik-cGAMP/HA derives mainly from the high GSH level in Hepa1-6 cells, which effectively triggers nanovaccines breakdown, resulting in GSH consumption and the release of drugs. In addition, the selective cytotoxicity of SRF@FeShik-cGAMP/HA may relate to the targeting of CD44 by HA. HA can effectively target tumor cells with high CD44 expression [30]. We use FITC-tagged SRF@FeShik-cGAMP and SRF@FeShik-cGAMP/HA to investigate the internalization of Hepa1-6 cells at different time points (Figs. S13 and S14). The flow cytometry results reveal that the cell uptake efficiency in the ^FITC^SRF@FeShik-cGAMP group is 13.0 ± 0.3%, 30.0 ± 5.2%, and 35.9 ± 1.5% at 1, 2, and 3 h, respectively, whereas in the ^FITC^SRF@FeShik-cGAMP/HA group, these values increase to 47.9 ± 0.1%, 90.7 ± 0.5%, and 91.6 ± 0.4%. The distinct difference indicates the specific uptake of ^FITC^SRF@FeShik-cGAMP/HA by Hepa1-6 cells, which is because HA can bind to CD44, a highly expressed HA receptor on the Hepa1-6 cell membrane, thus improving the active targeting capacity of ^FITC^SRF@FeShik-cGAMP/HA to Hepa1-6 cells. Furthermore, after Hepa1-6 cells are pre-cultured with excess free HA for 2 h, the internalization of ^FITC^SRF@FeShik-cGAMP/HA is significantly reduced to 19.6 ± 1.3%, 44.0 ± 3.0%, and 48.2 ± 0.5% at 1, 2, and 3 h, respectively. Besides, the CLSM visualization results are consistent with the flow cytometric results. The amount of ^FITC^SRF@FeShik-cGAMP or ^FITC^SRF@FeShik-cGAMP/HA internalized by Hepa1-6 cells is visualized by green fluorescence. As shown in Fig. S14c, compared with the weak green fluorescence of the ^FITC^SRF@FeShik-cGAMP, the ^FITC^SRF@FeShik-cGAMP/HA group exhibits much stronger green fluorescence intensity, and free HA markedly reduces the green fluorescence intensity of ^FITC^SRF@FeShik-cGAMP/HA. These results clearly validate that the enhanced uptake of SRF@FeShik-cGAMP/HA in Hepa1-6 cells is specifically mediated by HA–CD44 interactions, thereby facilitating selective cytotoxicity.

### Ferroptosis and necroptosis mechanisms

Given that the accumulation of Fe^2+^, SRF, and shikonin can elicit tumor cell death through ferroptosis and necroptosis [32, 33], the cytotoxicity mechanism of SRF@FeShik-cGAMP/HA toward Hepa1-6 cells is investigated. Since Fe^2+^ can catalyze H_2_O_2_ to produce ROS through the Fenton reaction, DCFH-DA is used as a fluorescent probe to detect intracellular ROS level. As expected, SRF@FeShik significantly increases intracellular ROS generation according to the release of Fe^2+^, SRF, and shikonin (Fig. S15a). Compared with SRF@FeShik-treated cells, Hepa1-6 cells treated with SRF@FeShik-HA or SRF@FeShik-cGAMP/HA present brighter green fluorescence in CLSM images. This occurs because SRF@FeShik-HA and SRF@FeShik-cGAMP/HA can effectively promote the generation of ROS by enhancing the accumulation of Fe^2+^, SRF, and shikonin, and achieving endogenous H_2_O_2_ self-replenishment in the presence of HA. The intracellular ROS level after treatment is further quantified by flow cytometry, which reveals that greater ROS production in the SRF@FeShik-HA and SRF@FeShik-cGAMP/HA groups (Fig. S15b). Subsequent expression analysis centered on genes associated with ROS indicates a notable upregulation of associated mRNAs and a significant decline in the expression of inhibitory genes (Fig. S16). The above data demonstrate that SRF@FeShik-cGAMP/HA can promote the Fenton reaction and ROS production by releasing Fe^2+^, SRF, and shikonin, and self-supplying H_2_O_2_ cascade, which is also beneficial for promoting ferroptosis.

**Fig. 2:**
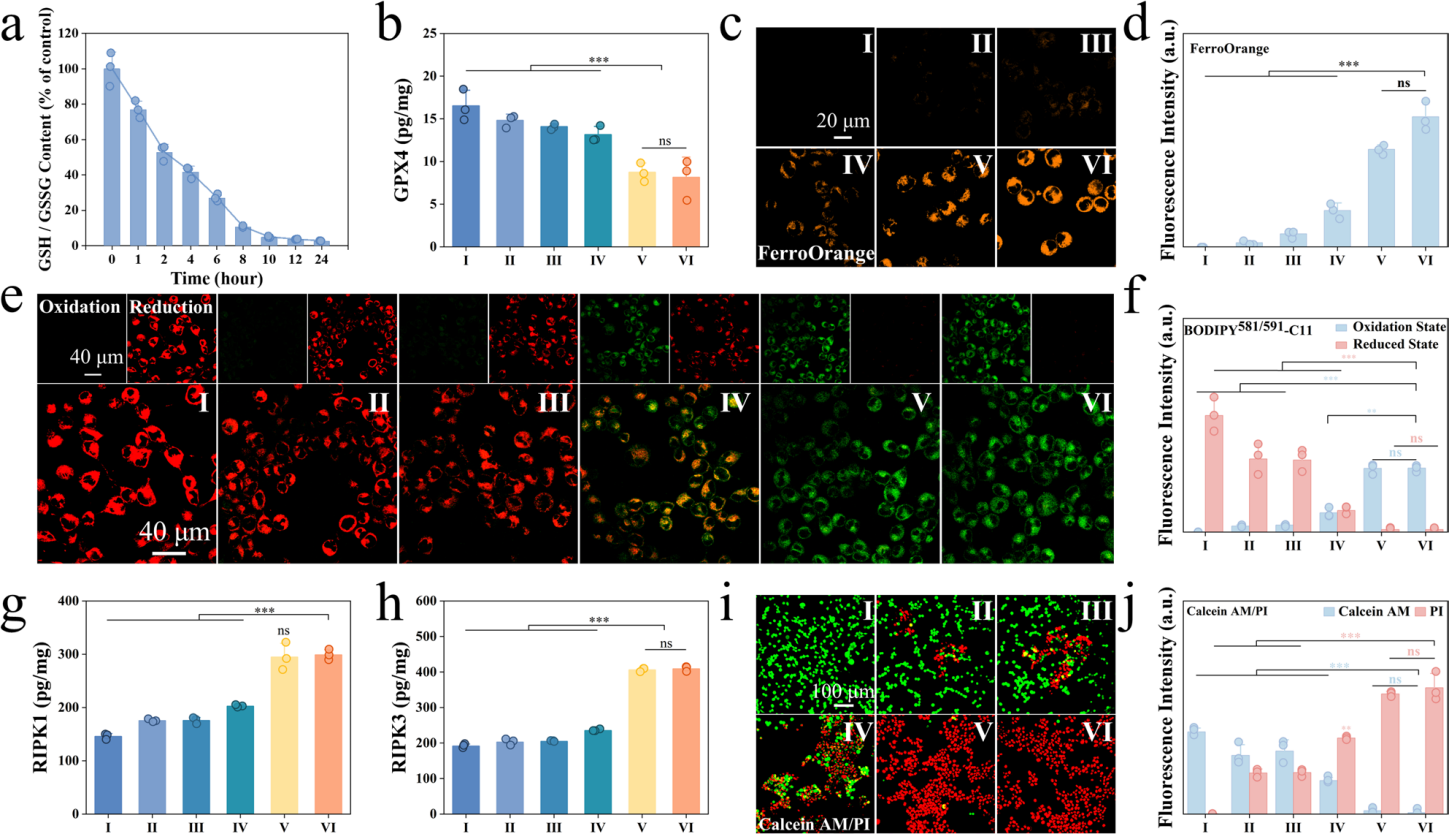
Cytotoxicity mechanisms of nanovaccines in vitro. **a** The changes in the GSH/GSSG ratio over time after SRF@FeShik-cGAMP/HA treatment (*n* = 3). **b** GPX4 expression levels of Hepa1-6 cells at different treatments (*n* = 3). **c** CLSM images of Hepa1-6 cells after incubation with FerroOrange. **d** Fluorescence intensity of FerroOrange CLSM images measured by ImageJ (*n* = 3). **e** CLSM examination of LPO accumulation in Hepa1-6 cells after treating with different treatments. **f** Fluorescence intensity of BODIPY^581/591^-C11 CLSM images measured by ImageJ (*n* = 3). Quantification of RIPK1 (**g**) and RIPK3 (**h**) expression levels in Hepa1-6 cells after different treatments by ELISA (*n* = 3). **i **Calcein AM and PI stained fluorescence imaging of Hepa1-6 cells subjected to various treatments. **j** Fluorescence intensity of Calcein AM/PI CLSM images measured by ImageJ (*n* = 3). Groups: (I) Control, (II) SRF, (III) FeShik, (IV) SRF@FeShik, (V) SRF@FeShik-HA, (VI) SRF@FeShik-cGAMP/HA

The ferroptosis inhibitor Fer-1 effectively rescues the cell death caused by SRF@FeShik-cGAMP/HA, which preliminarily confirms the occurrence of ferroptosis (Fig. S17). The depletion of GSH, the increase of Fe^2+^, the downregulation of GPX4, and the accumulation of LPO are important indicators of ferroptosis. The GSH and GSSG contents in Hepa1-6 cells from different groups are analyzed using a GSH and GSSG assay kit (Figs. 2a and S18). As previously mentioned, the decomposition of FeShik consumes GSH. Therefore, after FeShik treatment, the GSH content in Hepa1-6 cells is decreased, whereas the GSSG content is correspondingly increased. SRF can prevent system Xc- from transporting cystine into cells, and cystine is essential for the synthesis of GSH within cells. Thus, the presence of SRF can reduce the intracellular GSH level and increase the GSSG content to a certain extent. The consumption of GSH and the accumulation of GSSG in Hepa1-6 cells treated with SRF@FeShik are more pronounced than those treated with FeShik alone. Furthermore, HA increases GSH depletion and GSSG accumulation through cellular targeting and promoting the generation of H_2_O_2_. Accordingly, the GSH/GSSG ratio is significantly decreased in these groups, with the lowest ratio observed in the SRF@FeShik-HA and SRF@FeShik-cGAMP/HA groups. Importantly, following SRF@FeShik-cGAMP/HA treatment, the GSH/GSSG ratio exhibits a time‑dependent progressive decline, indicating a sustained disruption of cellular redox homeostasis (Fig. 2a). Gene set enrichment analysis (GSEA) indicates the GSH metabolism pathway is significant enriched (Fig. S19a). Additionally, expression analysis concentrating on genes involved in GSH metabolism shows an upregulation of pertinent mRNAs alongside a considerable reduction in the expression of inhibitory genes (Fig. S19b). GSH is a key regulator of GPX4 activity. Similarly, the expression of GPX4 in Hepa1-6 cells is significantly downregulated after treatment with SRF@FeShik-HA and SRF@FeShik-cGAMP/HA (Fig. 2b). Given that the intensity of FerroOrange fluorescence correlates directly with Fe^2+^ concentration, we utilize FerroOrange to assess intracellular Fe^2+^ levels. As shown in Fig. 2c and d, the orange fluorescence intensity of the SRF@FeShik-HA or SRF@FeShik-cGAMP/HA groups is significantly strong, suggesting that the constituent metal-polyphenol complexes in Hepa1-6 cells disassemble and are reduced to Fe^2+^. The accumulation of Fe^2+^ in Hepa1-6 cells presents a bright orange color. Since the production of ROS and the downregulation of GPX4 effectively accelerate the accumulation of LPO, the green fluorescence representing the oxidation state of the BODIPY^581/591^-C11 probe is enhanced in Hepa1-6 cells treated with SRF@FeShik-HA and SRF@FeShik-cGAMP/HA, while the red fluorescence representing the reduction state of the BODIPY^581/591^-C11 probe is weakened (Fig. 2e and f). Accordingly, as the main degradation product of LPO, the level of MDA in Hepa1-6 cells treated with SRF@FeShik-HA and SRF@FeShik-cGAMP/HA increases approximately ∼34-fold and ∼38-fold, respectively (Fig. S20). To further verify the role of iron accumulation, ROS, and LPO in the ferroptosis induced by SRF@FeShik-cGAMP/HA, we add DFO (an iron chelator), VC (a ROS reducer), and VE (a LPO scavenger) to analyze the cell viability. The results show that the cell death caused by SRF@FeShik-cGAMP/HA is partly rescued by these inhibitors. Specifically, the cell survival rate of the SRF@FeShik-cGAMP/HA group is 41.8 ± 4.9%, whereas the survival rates of the DFO, VC and VE groups are increased to 59.1 ± 8.6%, 58.5 ± 1.2%, and 56.2 ± 6.8%, respectively. (Fig. S17). These analyses demonstrate that SRF@FeShik-cGAMP/HA can induce Hepa1-6 cell death *via* self-amplifying ferroptosis.

SRF@FeShik-cGAMP/HA also causes tumor cell necroptosis by releasing shikonin. When the cells are pretreated with necroptosis inhibitor Nec-1, the cytotoxicity rate of SRF@FeShik-cGAMP/HA changes from 41.8 ± 4.9% to 58.7 ± 2.3% (Fig. S17). Meanwhile, the expression levels of necroptosis-related proteins, such as RIPK1 and RIPK3, are analyzed to evaluate necroptosis of Hepa1-6 cells under different treatments. The ELISA results reveal the upregulation of RIPK1 and RIPK3 in Hepa1-6 cells after SRF@FeShik-HA and SRF@FeShik-cGAMP/HA treatment (Fig. 2g and h). The above results strongly demonstrate that SRF@FeShik-cGAMP/HA can efficiently arouse necroptosis of Hepa1-6 cells.

The accumulation of excessive intracellular ROS leads to mitochondrial membrane depolarization. At high mitochondrial membrane potential, JC-1 forms aggregates and emits red fluorescence, whereas at low mitochondrial membrane potential, it exists as monomers and emits green fluorescence. Therefore, JC-1 serves as a reliable indicator of mitochondrial membrane potential changes. As shown in Fig. S21a and S21b, treatment with PBS alone shows almost no effect on mitochondrial membrane potential. Upon the addition of SRF or FeShik, mitochondrial membrane potential is reduced, and the green fluorescence intensity in the SRF@FeShik group is greater than that in the SRF or FeShik group, indicating that SRF@FeShik exerts a stronger effect in inducing oxidative stress. This is consistent with the significantly increased intracellular ROS production resulting from the release of Fe^2+^, SRF, and shikonin. The mitochondrial membrane potential in the SRF@FeShik-HA or SRF@FeShik-cGAMP/HA group is significantly lower than that in the SRF@FeShik group, as reflected by the red and green fluorescence signals in the CLSM images. The intracellular red/green fluorescence ratio of mitochondrial membrane potential is further quantified by flow cytometry, which confirms the lowest ratios in the SRF@FeShik-HA and SRF@FeShik-cGAMP/HA groups (Fig. S21c and S21d). Collectively, these results demonstrate that SRF@FeShik-cGAMP/HA induces ferroptosis and necroptosis in Hepa1-6 cells accompanied by mitochondrial dysfunction.

Calcein AM (green fluorescence) and PI (red fluorescence) are further used to co-stain live and dead cells, respectively, and to evaluate the effects of SRF@FeShik-cGAMP/HA on Hepa1-6 cells (Fig. 2i and j). The cells treated with SRF@FeShik-HA and SRF@FeShik-cGAMP/HA exhibit large areas of red fluorescence instead of green fluorescence. These results indicate that SRF@FeShik-cGAMP/HA can effectively induce cell death through ferroptosis and necroptosis.

**Fig. 3:**
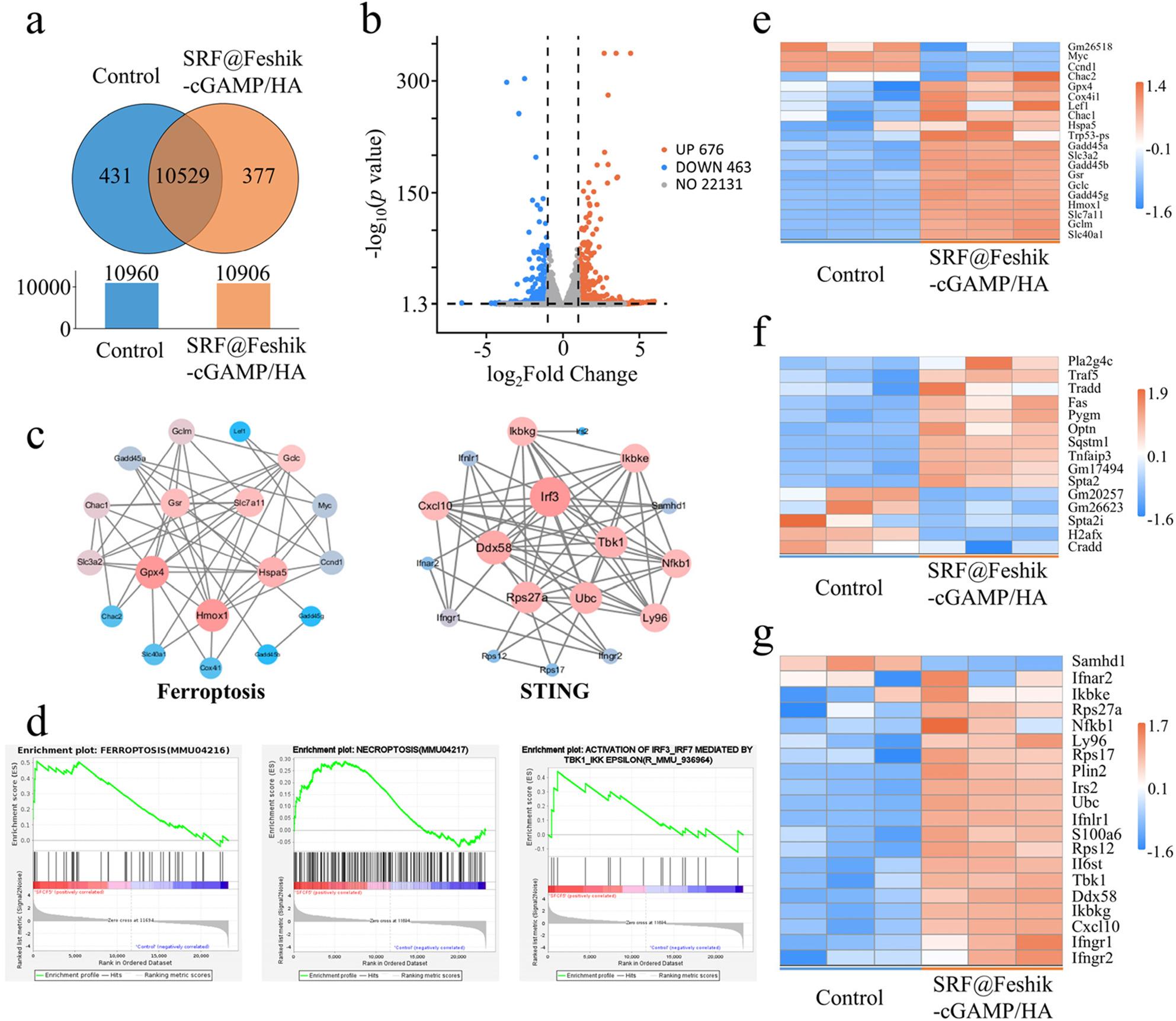
RNA-seq analysis. **a** Venn diagram of all expressed genes in each group. **b** Volcano plot of the distributions of differentially expressed genes after SRF@FeShik-cGAMP/HA treatment (|log_2_(Fold Change)| > =1 and *p*-value <= 0.05). **c** Protein-protein interaction network from the STRING database. **d** GSEA analysis of differentially expressed genes that hit the ferroptosis, necroptosis and STING pathway gene sets. Heat-map analysis of mRNA expression levels of genes involved in ferroptosis (**e**), necroptosis (**f**) and STING pathway (**g**)

To delve deeper into the cytotoxic mechanism of SRF@FeShik-cGAMP/HA nanovaccines, we conduct gene expression analysis in post-treatment Hepa1-6 cells using RNA-seq. As shown in the Venn diagram, a total of 10,529 genes is detected in both groups of samples, while 377 genes are only expressed in the SRF@FeShik-cGAMP/HA group (Fig. 3a). Principal component analysis (Fig. S22a) and the volcano plot (Fig. 3b) demonstrate a marked difference in gene expression between the two groups, suggesting that SRF@FeShik-cGAMP/HA notably influences the molecular biological mechanisms within Hepa1-6 cells. To explore the pathways link to SRF@FeShik-cGAMP/HA-induced ferroptosis and necroptosis, we carry out Kyoto Encyclopedia of Genes and Genomes (KEGG) analysis to pinpoint enriched gene sets. Numerous cellular pathways exhibit significant upregulation following SRF@FeShik-cGAMP/HA treatment, particularly those related to ferroptosis, necroptosis, and TNF signaling pathway (Fig. S22b). The network depicting protein-protein interactions among differentially expressed genes highlights strong connections, emphasizing their combined influence on cellular functions (Figs. 3c and S23). GSEA reveals substantial upregulation of pathways such as ferroptosis and necroptosis (Fig. 3d). Additional expression analysis targeting genes connected to ferroptosis and necroptosis indicates upregulation of relevant mRNAs and a significant decrease in the expression of inhibitory genes (Fig. 3e and f). These findings confirm that SRF@FeShik-cGAMP/HA nanovaccines not only effectively trigger ferroptosis but also activate necroptosis.

### Cooperation of ferroptosis and necroptosis induced ICD with STING activation for DC maturation

**Fig. 4:**
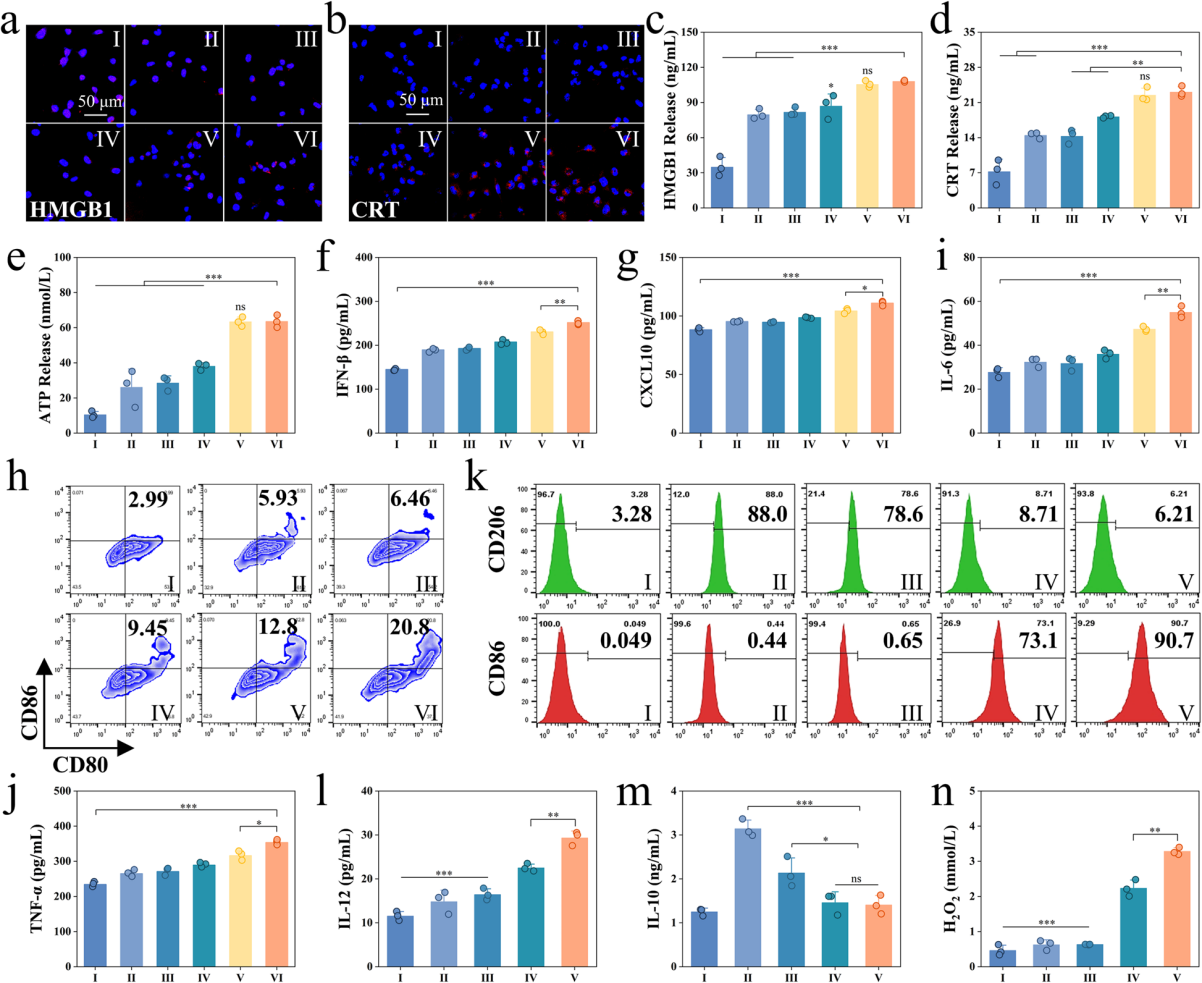
In vitro immune response study. CLSM images of HMGB1 release (**a**) and CRT exposure (**b**) in Hepa1-6 cells after different treatments. Quantification of HMGB1 (**c**) and CRT (**d**) expression levels after different treatments by ELISA (*n* = 3). **e** Quantification of ATP secretion levels in cell medium after different treatments by ATP assay kit (*n* = 3). Quantification of IFN-β (**f**) and CXCL10 (**g**) expression levels after different treatments by ELISA (*n* = 3). **h** Representative flow cytometric plots of DC maturation (CD11c^+^CD80^+^CD86^+^, gated on CD11c^+^ cells) after different treatments. Quantification of IL-6 (**i**) and TNF-α (**j**) expression levels after different treatments by ELISA (*n* = 3). **k** Flow cytometric results of the expression of CD86 (M1 macrophages) and CD206 (M2 macrophages) after treatment with different formulations. Levels of IL-12 (**l**) and IL-10 (**m**) secreted by macrophages in the different treatment groups (*n* = 3). **n** Quantitative determination of H_2_O_2_ production in the different groups (*n* = 3). Groups in a-j: (I) Control, (II) SRF, (III) FeShik, (IV) SRF@FeShik, (V) SRF@FeShik-HA, (VI) SRF@FeShik-cGAMP/HA. Groups in k-n: (I) M0, (II) M2, (III) SRF@FeShik + M2, (IV) SRF@FeShik-HA + M2, (V) SRF@FeShik-cGAMP/HA + M2

Ferroptosis and necroptosis can trigger ICD in tumor cells. Thus, distinct ICD-related biochemical hallmarks, including cell-surface exposure of CRT, the release of HMGB1 and the extracellular secretion of adenosine triphosphate (ATP), are analyzed to evaluate the ICD effect of SRF@FeShik-cGAMP/HA [34, 35]. HMGB1 is a nuclear protein, as shown in Fig. 4a. The red fluorescence signals in the control group are strong and nearly colocalize with the blue-stained cell nuclei. However, in the SRF-, FeShik-, SRF@FeShik-, SRF@FeShik-HA- and SRF@FeShik-cGAMP/HA-treated groups, the intensity of the nuclear red fluorescence signals gradually diminishes, while the red fluorescence signals outside the nucleus gradually increase, especially in the SRF@FeShik-HA- and SRF@FeShik-cGAMP/HA-treated groups. In the SRF@FeShik-HA- and SRF@FeShik-cGAMP/HA-treated groups, almost all of the red fluorescence signals surround the cell nuclei, suggesting that HMGB1 is released from the nuclei into the extracellular environment. As demonstrated in Fig. 4b, Hepa1-6 cells are labeled with an anti-CRT antibody. As anticipated, noticeable red fluorescence signals are detected in the SRF@FeShik-HA- and SRF@FeShik-cGAMP/HA-treated groups, weaker red fluorescence signals are observed in the SRF, FeShik and SRF@FeShik groups, and negligible red fluorescence signals are observed in the control group, indicating the exposure of CRT in the SRF-, FeShik-, SRF@FeShik-, SRF@FeShik-HA- and SRF@FeShik-cGAMP/HA-treated groups, especially the SRF@FeShik-HA- and SRF@FeShik-cGAMP/HA-treated groups. ELISA further reveals that SRF@FeShik-HA and SRF@FeShik-cGAMP/HA significantly increase the level of HMGB1 release (3.0-fold and 3.1-fold of the control) and CRT exposure (3.1-fold and 3.2-fold of the control) (Fig. 4c and d). The later detection of ATP secretion shows a trend consistent with that observed during CRT exposure. ATP secretion is significantly increased in Hepa1-6 cells treated with SRF@FeShik-HA (6.1-fold of the control) and SRF@FeShik-cGAMP/HA (6.1-fold of the control) (Fig. 4e). These findings confirm that SRF@FeShik-cGAMP/HA can evoke a strong ICD response through inducing ferroptosis and necroptosis in Hepa1-6 cells.

To confirm the immune responses triggered by SRF@FeShik-cGAMP/HA through the STING-dependent signaling pathway, the fluorescence signal of p-STING in Hepa1-6 cells is detected by CLSM. Compared with other groups, the SRF@FeShik-cGAMP/HA group shows a significant increase in green fluorescence signals (Fig. S24). Moreover, following various treatments, the concentrations of proinflammatory cytokines IFN-β and CXCL10, key markers of type I interferons in the STING pathway [36], are assessed in the supernatants obtained from cancer cell samples. As shown in Fig. 4f and g, the concentrations of IFN-β and CXCL10 in the SRF@FeShik-cGAMP/HA-treated group increase markedly. Furthermore, the mechanism of STING pathway is investigated *via* RNA-seq upon SRF@FeShik-cGAMP/HA nanovaccines treatment. To determine the pathways underlying SRF@FeShik-cGAMP/HA-induced STING, we perform Reactome analysis to identify enriched gene sets. Activation of IRF3 mediated by TBK1, IKBKE signaling pathways show significant upregulation after SRF@FeShik-cGAMP/HA treatment (Fig. S22b). The network of protein-protein interactions among differentially expressed genes indicates close associations, underscoring their collective impact on cellular processes (Fig. 3c). GSEA indicates significant upregulation of STING pathway (Fig. 3d). Additionally, an analysis of gene expression concerning the STING pathway indicates an increase in relevant mRNA levels alongside a significant reduction in the expression of inhibitory genes (Fig. 3g). These findings confirm that SRF@FeShik-cGAMP/HA nanovaccines effectively activate the STING pathway.

The ICD provides DCs with sufficient antigen signals and activation signals. The activation of the STING pathway promotes the secretion of type I interferons, further amplifying the immune activation cascade. The inflammatory signals emitted by dying tumor cells are identified by DCs, which are essential in both innate and adaptive immune responses. Subsequently, mature DCs engage naive T cells, triggering immune responses against tumors [37]. Based on this, the combined effects of ferroptosis and necroptosis-induced ICD and the stimulation of the STING pathway may improve DC maturation as well as activate the immune system. The process of DC maturation is characterized by an increase in the expression of costimulatory molecules such as CD80 and CD86, alongside the secretion of proinflammatory cytokines like IL-6 and TNF-α [38]. DC maturation is investigated by co-incubating BMDCs with Hepa1-6 cells under different treatments. Cells are harvested for flow cytometry evaluation, and the culture medium is obtained to measure the secretion levels of IL-6 and TNF-α. The flow cytometry results indicate that, when compared to the control group, the proportion of mature DCs increases to 12.8% following treatment with SRF@FeShik-HA, which represents a 4.3-fold increase relative to the control group (Fig. 4h). These results indicate that SRF@FeShik-HA-induced ICD can stimulate DC maturation. Surprisingly, the percentage of mature DCs further increases to 20.8% with SRF@FeShik-cGAMP/HA treatment, which is attributed to cooperation of ferroptosis and necroptosis mediated ICD with STING pathway activation. Additionally, there is a significant rise in the levels of IL-6 and TNF-α (Fig. 4i and j). These results indicate the strong ability of SRF@FeShik-cGAMP/HA nanovaccines to promote the maturation of DCs, underscoring its enhanced immunomodulatory effectiveness in the treatment of tumors.

### Tumor-associated macrophages polarization

Beyond its CD44-mediated tumor targeting capability, HA concurrently reprograms tumor-associated macrophages (TAMs) from immunosuppressive M2 to pro-inflammatory M1 phenotype. Meanwhile, the immune adjuvant cGAMP directly promotes M1 polarization by activating the STING pathway and generating type I interferons. M1 macrophages release cytotoxic H_2_O_2_ to achieve endogenous H_2_O_2_ self-replenishment. To investigate the regulatory effects of SRF@FeShik-HA and SRF@FeShik-cGAMP/HA on the phenotypic transition of M2 macrophages, we assess the expression levels of the M1 phenotypic marker (CD86) and M2 phenotypic marker (CD206) on macrophage surfaces *via* flow cytometry. Results demonstrate that following SRF@FeShik-HA treatment, CD86 expression in M2 macrophages is significantly upregulated to 73.1%, while CD206 expression is notably downregulated to 8.71% (Fig. 4k). This finding confirms that HA can effectively induce the polarization of M2 macrophages into pro-inflammatory M1 macrophages. More notably, the SRF@FeShik-cGAMP/HA treatment group exhibits a more pronounced regulatory effect on macrophage phenotypic transition, it further elevates CD86 expression to 90.7% while reducing CD206 expression to 6.21%. This observation suggests that cGAMP can synergistically enhance HA-mediated macrophage polarization by activating the STING pathway, thereby reversing the immunosuppressive state within the TME to the maximum extent.

To further validate the regulatory specificity of SRF@FeShik-cGAMP/HA toward TAMs subtypes, we quantify the secretion levels of M1-characteristic (Interleukin-12, IL-12) and M2-characteristic (Interleukin-10, IL-10) cytokines by macrophages using ELISA kit (Fig. 4l and m). The results reveal that the variation trend of the cytokine secretion profile is fully consistent with the flow cytometry data. This confirms, at the functional level, that SRF@FeShik-cGAMP/HA can efficiently and stably induce the phenotypic reprogramming of M2 macrophages toward the M1 phenotype, providing critical experimental evidence for subsequent studies on tumor immune microenvironment modulation. Moreover, the generation of H_2_O_2_ in the TME is evaluated by a H_2_O_2_ assay kit, and the data indicate a significantly increased H_2_O_2_ concentration in the SRF@FeShik-cGAMP/HA + M2 (5.2-fold) and SRF@FeShik-HA + M2 (3.7-fold) groups compared with the M2 group (Fig. 4n). These results indicate that SRF@FeShik-cGAMP/HA can effectively polarize M2 macrophages toward the M1 phenotype and produce H_2_O_2_, thus achieving endogenous H_2_O_2_ self-replenishment and promoting the ferroptosis. It demonstrates that our nanovaccines form a powerful anti-tumor immune response *via* a synergistic amplification mechanism.

### Pharmacokinetics and biodistribution

To study the pharmacokinetics and biodistribution of the nanovaccines, SRF@FeShik-cGAMP and SRF@FeShik-cGAMP/HA are labeled with IR780 to construct ^IR780^SRF@FeShik-cGAMP and ^IR780^SRF@FeShik-cGAMP/HA, respectively. The fluorescence of IR780 in blood samples taken from mice at different time intervals post-intravenous injection is measured, resulting in half-life times (*t½*) for ^IR780^SRF@FeShik-cGAMP and ^IR780^SRF@FeShik-cGAMP/HA of 4.0 ± 0.2 h and 6.3 ± 0.1 h, respectively (Fig. S25). Compared with that of ^IR780^SRF@FeShik-cGAMP, the blood circulation time of ^IR780^SRF@FeShik-cGAMP/HA is significantly prolonged. This is mainly due to the prolonged systemic circulation *via* HA-mediated stealth properties and CD44-target active delivery ability of ^IR780^SRF@FeShik-cGAMP/HA, ensuring effective and simultaneous accumulation in tumor tissues.

In vivo fluorescence images of mice, displayed in Fig. S26a, illustrate the results following the intravenous administration of ^IR780^SRF@FeShik-cGAMP and ^IR780^SRF@FeShik-cGAMP/HA. The accumulation of ^IR780^SRF@FeShik-cGAMP/HA within tumor tissues occurs gradually over the first 24 h, peaks at 36 h after injection, and persists at elevated levels even 48 h post-injection (Fig. S26b). In sharp contrast, mice injected with ^IR780^SRF@FeShik-cGAMP demonstrate persistently low fluorescence signals in tumor tissues throughout the entire monitoring duration. Ex vivo imaging of fluorescence in major organs and tumors validates that ^IR780^SRF@FeShik-cGAMP/HA displays a pronounced fluorescence signal inside the tumor at the 48-hour mark, while the fluorescence from ^IR780^SRF@FeShik-cGAMP/HA mainly gathered in the liver and lungs. The semi-quantitative assessment indicates that the fluorescence intensity in tumors treated with ^IR780^SRF@FeShik-cGAMP/HA is 1.8-fold greater than that in those treated with ^IR780^SRF@FeShik-cGAMP (Fig. S26c). These results indicate that SRF@FeShik-cGAMP/HA is effectively concentrated within tumor tissues, enhancing its therapeutic efficacy and potential for targeted tumor treatment.

As iron exposure is central to both antitumor efficacy and potential toxicity, the Fe pharmacokinetic and biodistribution are analyzed by ICP-MS. The plasma iron concentration profile and calculated key pharmacokinetic parameters including Cmax, AUC, *t½*, and clearance are performed (Fig. S27a, S27b and Table S1). The results show that SRF@FeShik-cGAMP/HA exhibits significantly prolonged blood circulation, with Cmax, AUC (0-t), AUC (0-∞), *t½*, and clearance values of 90.5 ± 3.5 µg/L, 759.0 ± 6.3 µg/L*h, 807.8 ± 11.8 µg/L*h, 6.0 ± 0.2 h, 12.4 ± 0.2 L/h/kg, respectively. For time-dependent biodistribution, quantitative Fe analysis reveals that SRF@FeShik-cGAMP/HA achieves high tumor accumulation, with a peak level of 14.9 ± 0.4% ID/g at 36 h post-injection (Fig. S27c and S27d). At the same time point, the tumor accumulation of SRF@FeShik-cGAMP is 8.7 ± 2.0% ID/g, further confirming the superior tumor-targeting ability of SRF@FeShik-cGAMP/HA. At 96 h post-injection of SRF@FeShik-cGAMP/HA, the Fe levels in major organs (heart, liver, spleen, lungs, kidneys) are 2.6 ± 0.7% ID/g, 5.2 ± 1.6% ID/g, 2.7 ± 0.8% ID/g, 6.3 ± 2.3% ID/g, and 3.0 ± 0.4% ID/g, respectively, with no significant differences compared with the SRF@FeShik‑cGAMP group, suggesting negligible off-target iron accumulation in healthy tissues. These data demonstrate that Fe is efficiently delivered and retained in tumors at levels consistent with our proposed mechanism, without excessive systemic exposure.

### Antitumor effect* in vivo*

**Fig. 5:**
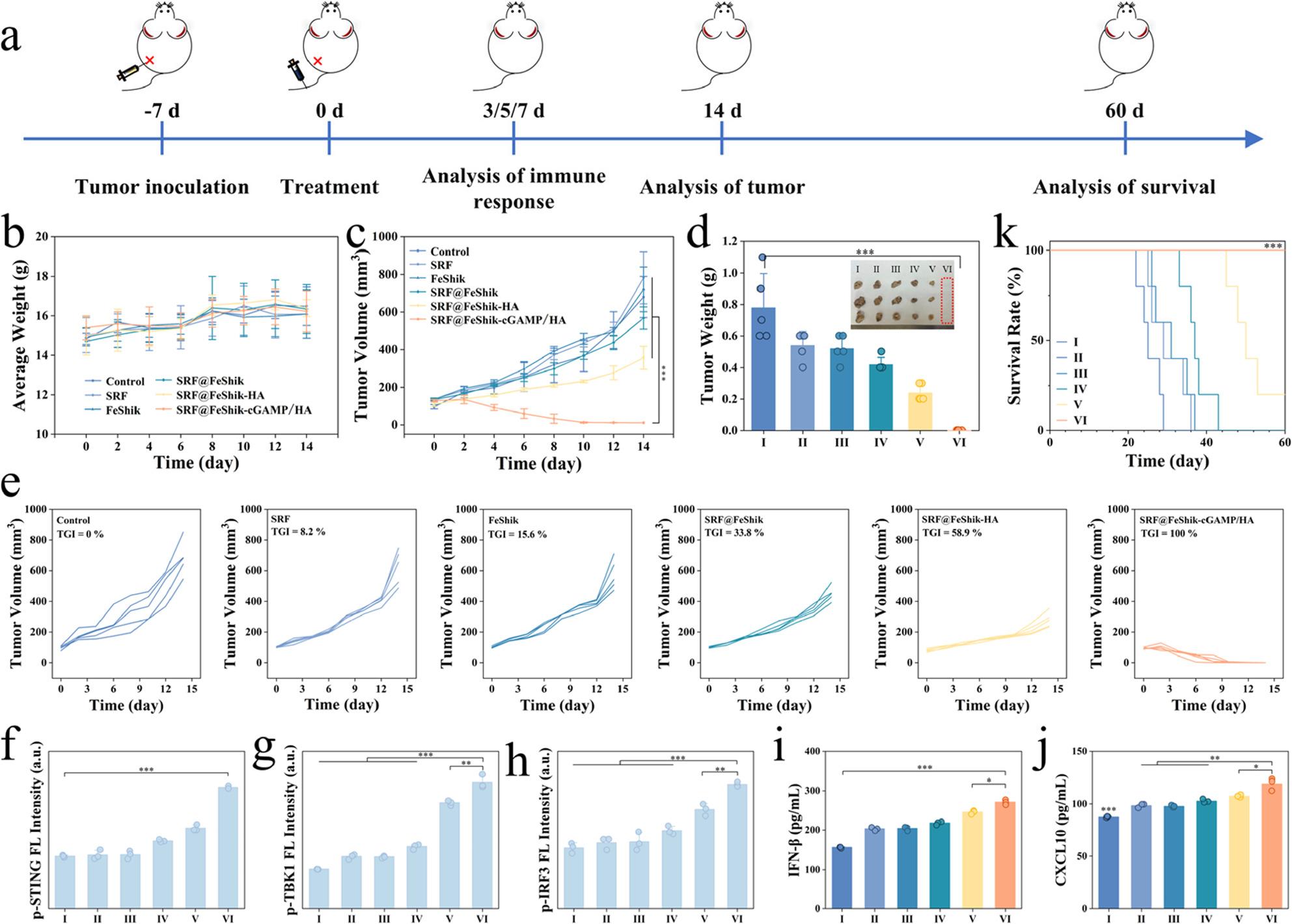
*In vivo* antitumor evaluation. **a** Schematic illustration of the tumor treatment process *in vivo*. Body weight (**b**) and tumor volume (**c**) changes in the different groups (*n* = 5). **d** Tumor weights after different treatments (*n* = 5). Insert: optical images of tumor after different treatments (*n* = 3). **e** Individual tumor growth kinetics in different groups (*n* = 5). Fluorescence intensity of p-STING (**f**), p-TBK1 (**g**), and p-IRF3 (**h**) fluorescence images measured by ImageJ (*n* = 3). The levels of IFN-β (**i**) and CXCL10 (**j**) in mouse serum after various treatments (*n* = 3). **k** Survival curves of mice under different treatments (*n* = 5). Groups: (I) Control, (II) SRF, (III) FeShik, (IV) SRF@FeShik, (V) SRF@FeShik-HA, (VI) SRF@FeShik-cGAMP/HA

The in vivo therapeutic efficacy of SRF@FeShik-cGAMP/HA is evaluated in Hepa1-6 tumor-bearing C57BL/6J mice (Fig. 5a). When the tumors reach ~ 100 mm^3^, the mice are randomly divided into six groups according to different treatments: (I) Control, (II) SRF, (III) FeShik, (IV) SRF@FeShik, (V) SRF@FeShik-HA, and (VI) SRF@FeShik-cGAMP/HA. All experimental groups show no irregularities in body weight (Fig. 5b). Tumor volumes in the mice are assessed every two days throughout the course of the experiment. The tumor volume data indicate that the tumors in the control group grow rapidly. The SRF and FeShik monotherapy groups demonstrate constrained cytotoxic efficacy, as evidenced by persistent tumor progression despite concomitant induction of the ferroptosis and necroptosis pathways. The HA-assisted SRF@FeShik-HA group exhibits a significantly lower tumor growth rate compared with the other groups, resulting in a pronounced inhibitory effect, and a tumor inhibition rate of 58.9% (Fig. 5c and e). This result might be because SRF@FeShik-HA is CD44-responsive to increase the accumulation of nanovaccines at tumor sites and promote the generation of endogenous H_2_O_2_ to achieve self-amplifying ferroptosis. Furthermore, compared with SRF@FeShik-HA, SRF@FeShik-cGAMP/HA has a stronger inhibitory effect on tumor growth, and the tumor inhibition rate is up to 100%. This might be because SRF@FeShik-cGAMP/HA activates the STING pathway, and the dual ferroptosis and necroptosis-mediated ICD as well as the activation of the STING pathway produce a synergistic immune enhancement effect, which has significant potential for stimulating antitumor immune responses and alleviating immunosuppression. This powerful immune reaction is essential for the antitumor effect observed in mice. Additionally, the results regarding tumor weight and representative images of tumors dissected 14 days post-treatment offer further visual proof of the significant effectiveness of SRF@FeShik-cGAMP/HA in hampering tumor growth (Fig. 5d). This observation aligns with the outcome observed in terms of the relative alteration of tumor suppression rate and tumor growth curve (Fig. 5e), providing unequivocal validation for the synergy of dual ferroptosis and necroptosis in combination with activation of the STING pathway, which ultimately results in potentiated tumoricidal efficacy. Subsequently, assays using H&E staining are performed to evaluate the effectiveness of the treatment in inhibiting tumor growth. As shown in Fig. S28, abundant necrosis is observed in the SRF@FeShik-HA- and SRF@FeShik-cGAMP/HA-treated groups. In the groups treated with SRF, FeShik and SRF@FeShik, the areas of necrosis decrease gradually, and almost no necrosis is observed in the control group. These results are consistent with relative alteration of tumor volume, which together indicate that our nanovaccines exhibit potent potential to suppress tumor proliferation.

To study the mechanism by which the nanovaccines cause ferroptosis and necroptosis, we use immunofluorescence to detect the expressions of GPX4, RIPK1 and RIPK3 in tumor tissues following different treatments. As shown in Fig. S29 and S30, the fluorescence signals of RIPK1 and RIPK3 are increased and those of GPX4 are decreased in the SRF@FeShik-cGAMP/HA group, suggesting that SRF@FeShik-cGAMP/HA promotes the expression of necroptosis-related RIPK1 and RIPK3 but suppress the expression of ferroptosis-related GPX4 in tumors [39]. Fe-driven ROS production and oxidative damage are further investigated. The ROS levels are evaluated using DHE fluorescence staining (Fig. S31a and S31b). The SRF@FeShik-cGAMP/HA group shows significantly enhanced fluorescence signals in tumor tissues, indicating robust ROS generation *via* Fe-mediated Fenton chemistry. In addition, LPO is detected using BODIPY^581/591^-C11 (Fig. S31a and S31c). Compared with other groups, the SRF@FeShik-cGAMP/HA treatment group exhibits markedly decreased red fluorescence (the reduced state), confirming severe lipid peroxidation in tumors. These results indicate that SRF@FeShik-cGAMP/HA induces ferroptosis and necroptosis in tumor tissues. Ferroptosis and necroptosis release DAMPs, leading to the activation of immune responses associated with non-apoptotic pathways of ICD. As shown by the immunofluorescence results (Fig. S32), the CRT related green immunofluorescence signals exhibit progressive increases in the following order: the control, SRF, FeShik, SRF@FeShik, SRF@FeShik-HA, and SRF@FeShik-cGAMP/HA groups. Concurrently, the green immunofluorescence signals indicating HMGB1 within the cell nuclei decrease and release into the cytoplasm in the same order (Fig. S33). These results are consistent with those obtained from the cell experiments, suggesting that SRF@FeShik-cGAMP/HA not only eradicates tumors *via* ferroptosis and necroptosis but also effectively induces ICD.

The activation of STING is characterized by the phosphorylation of STING, TBK1, and IRF3 [40]. Therefore, to confirm our hypothesis, we conduct immunofluorescence staining of tumor tissues for p-STING, p-TBK1, and p-IRF3 following various treatments (Figs. 5f-h, and S34). The results show that the green immunofluorescence signal intensity in the tumor tissue sections from mice treated with SRF@FeShik-cGAMP/HA is greater than that in the other groups, indicating that the increased expression of STING-related proteins in tumor tissues can be attributed to the presence of cGAMP. Moreover, the levels of IFN-β (1.7-fold of control) and CXCL10 (1.4-fold of control) in the serum of mice are higher in the SRF@FeShik-cGAMP/HA group compared to the other groups, as determined by ELISA, indicating the activation of the STING pathway (Fig. 5i and j).

To conclude, our experiments have confirmed the antitumor mechanisms associated with SRF@FeShik-cGAMP/HA. Specifically, these nanovaccines not only elicit potent ferroptosis and necroptosis responses, inducing strong ICD through non-apoptotic pathways, but also triggers activation of the STING pathway. This complex interaction involving ferroptosis, necroptosis, and STING activation leads to the effective removal of cancerous cells.

The survival times of the mice are recorded to evaluate whether SRF@FeShik-cGAMP/HA treatment can extend their survival rate (Fig. 5k). Survival durations are notably prolonged in all treatment groups when compared to the control group, especially in the SRF@FeShik-cGAMP/HA-treated group. All mice in the control, SRF, FeShik and SRF@FeShik groups have died by the 60th day, with only one mouse surviving in the SRF@FeShik-HA group, while the SRF@FeShik-cGAMP/HA group still has 100% survival rate. Compared to the SRF@FeShik-HA group, the survival time of the SRF@FeShik-cGAMP/HA group is significantly increased, which relies on the synergistic antitumor immune responses of dual ferroptosis, necroptosis-mediated ICD and activation of the STING pathway. In addition, an analysis of H&E-stained images from key organs taken from mice following 14 days of treatment shows no clear signs of pathological damage or inflammatory lesions, suggesting that there are minimal adverse effects throughout the tumor treatment process (Fig. S35). These findings highlight the strong antitumor effectiveness of our nanovaccines, resulting in a notable increase in lifespan.

### Remodeling the tumor immune microenvironment and potentiating anti-tumor immunity

In our study, we aim to explore and evaluate the concerted immunostimulatory effects elicited by combined ferroptosis, necroptosis and STING activation. Building on our previous findings from cellular experiments that demonstrate the potential of our SRF@FeShik-cGAMP/HA nanovaccines to enhance DCs maturation, we proceed to investigate the in vivo immunostimulatory effects of these nanovaccines. This involves the collection of blood, tumor, and spleen tissues to evaluate pertinent immune markers.

**Fig. 6:**
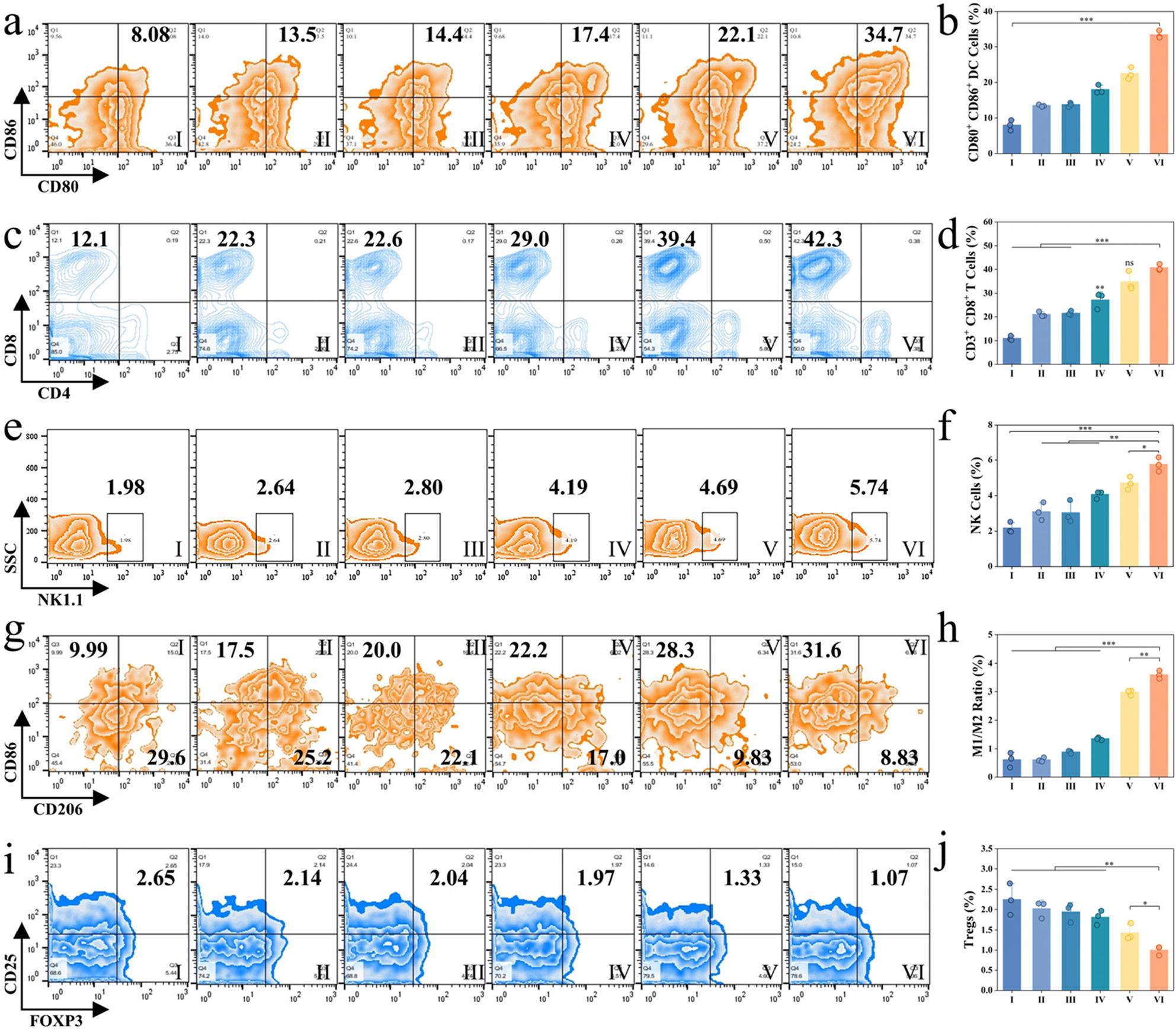
*In vivo* immune response study. Flow cytometric analysis (**a**) and corresponding quantification (**b**) of DC maturation (CD11c^+^CD80^+^CD86^+^) in spleens after different treatments (*n* = 3). Flow cytometric analysis (**c**) and corresponding quantification (**d**) of CTLs (CD3^+^CD8^+^) in tumors after different treatments (*n* = 3). Flow cytometric analysis (**e**) and corresponding quantification (**f**) of NK cells (CD45^+^CD3^−^NK1.1^+^) in tumor after different treatments (*n* = 3). Flow cytometric analysis (**g**) and corresponding quantification (**h**) of M1/M2 macrophages in tumor after different treatments (*n* = 3). Flow cytometric analysis (**i**) and corresponding quantification (**j**) of Tregs (CD4^+^CD25^+^ Foxp3^+^) in tumors after different treatments. Groups: (I) Control, (II) SRF, (III) FeShik, (IV) SRF@FeShik, (V) SRF@FeShik-HA, (VI) SRF@FeShik-cGAMP/HA

The flow cytometry results indicate that the proportion of mature DCs (CD11c^+^CD80^+^CD86^+^ ) in the spleen of mice treated with SRF@FeShik-HA is approximately 22.5 ± 1.7%, which is more than twice as high as that in the control group (8.0 ± 1.5%) (Fig. 6a and b) [41]. In addition, SRF@FeShik-cGAMP/HA induces the highest level of DCs maturation (33.4 ± 1.1%), which may be due to synergistic immune-enhancing effects of ferroptosis, necroptosis and STING pathway activation. Furthermore, the quantification of TNF-α and IL-6 levels in mouse serum *via* ELISA demonstrates a similar pattern to the findings from flow cytometry, further indicating the maturation of DCs (Fig. S36). To summarize, the previously discussed studies offer strong proof of the effective ability of the developed SRF@FeShik-cGAMP/HA nanovaccines to promote the maturation of DCs, thus laying a robust foundation for the future production of CTLs.

Considering that activated DCs are capable of stimulating T lymphocytes and provoking potent adaptive immune responses, we explore the infiltration of CD8^+^ T cells within the tumor tissues of mice subjected to different treatments. Compared to those in the control group, the SRF@FeShik-cGAMP/HA group exhibits a significant increase in the occurrence rate of CD8^+^ T cells in tumors, followed by the SRF@FeShik-HA group and the SRF@FeShik group, suggesting an enhanced systemic immune response (Fig. 6c and d). In addition, the immunofluorescence results of the CD8^+^ T cell content in tumors are consistent with the flow cytometry data (Fig. S37). These findings indicate that SRF@FeShik, SRF@FeShik-HA or SRF@FeShik-cGAMP/HA induces systematic immune responses to varying degrees.

Abundant studies report that type I interferon serves as a critical bridge linking innate and adaptive immunity. It directly activates NK cells, enhances their cytotoxic activity, and stimulates IFN-γ secretion [42–45], thereby mediating tumor cell killing and paving the way for T cell-driven, memory-pronged adaptive immune responses. As a key driver of macrophage polarization, type I interferon directly promotes M1 polarization [46, 47], while IFN-γ, the strongest known inducer of M1 polarization, can indirectly promote M1 polarization [48]. In addition, type I interferon can also directly impair the immunosuppressive capacity of Tregs [49–51], disrupting immune homeostasis in favor of immune clearance. The resulting M1 macrophages counteract the immunosuppressive milieu established by M2 macrophages and Tregs, thereby converting the TME from an immunologically “cold” tumor to a “hot” tumor, ultimately enhancing the efficacy of antitumor immunotherapy [52]. Fig. 6e and f show the levels of tumor-infiltrating NK cells in the tumors under different treatments. The percentage of infiltrating NK cells (CD45^+^CD3^−^NK1.1^+^) increases to 5.8 ± 0.4% under SRF@FeShik-cGAMP/HA treatment. In addition, the IFN-γ level in the mouse serum of the SRF@FeShik-cGAMP/HA group is 1.9-fold that of the control group (Fig. S38). The above results indicate that the type I interferons produced by the STING pathway activated by SRF@FeShik-cGAMP/HA effectively activate NK cells. Moreover, SRF@FeShik-cGAMP/HA has the capability to shift M2 macrophages towards the M1 phenotype, thus reversing immunosuppression. As illustrated in Fig. S39, the intensity of red fluorescence in M1 macrophages within the SRF@FeShik-cGAMP/HA group is markedly higher compared to the other groups, while the intensity of green fluorescence in M2 macrophages in the SRF@FeShik-cGAMP/HA group is considerably reduced. Meanwhile, flow cytometry reveals that the M1/M2 ratio is significantly increased in the SRF@FeShik-cGAMP/HA group compared with that in the other groups (Fig. 6g and h). This increase is due to the release of HA from SRF@FeShik-cGAMP/HA and the production of type I interferons, which synergistic accelerate the formation of M1 macrophages. These results indicate that SRF@FeShik-cGAMP/HA can significantly polarize M2 macrophages toward the M1 phenotype and reverse immunosuppression. Fig. 6i and j show the percentages of Tregs (CD4^+^CD25^+^ Foxp3^+^) in the tumors. Compared with those in the control group (2.3 ± 0.4%), the abundance of Tregs in SRF (2.1 ± 0.2%), FeShik (1.9 ± 0.2%), SRF@FeShik (1.8 ± 0.2%), SRF@FeShik-HA (1.4 ± 0.2%), and SRF@FeShik-cGAMP/HA (1.0 ± 0.1%) groups are decreased. The SRF@FeShik-cGAMP/HA group shows the lowest frequency of Tregs. The corresponding immunofluorescence images show that SRF@FeShik-cGAMP/HA significantly reduces the number of Foxp3^+^ cells (Fig. S40), indicating relief from immunosuppression.

Overall, the above results demonstrate the significant potential of SRF@FeShik-cGAMP/HA to remodel the tumor immune microenvironment and potentiate anti-tumor immunity. These findings elucidate the synergistic immune-enhancing effects of dual ferroptosis and necroptosis-mediated ICD and STING pathway activation. This potent immune response plays a crucial role in anti-tumor effect of mice.

### Long-term immune memory effect

**Fig. 7:**
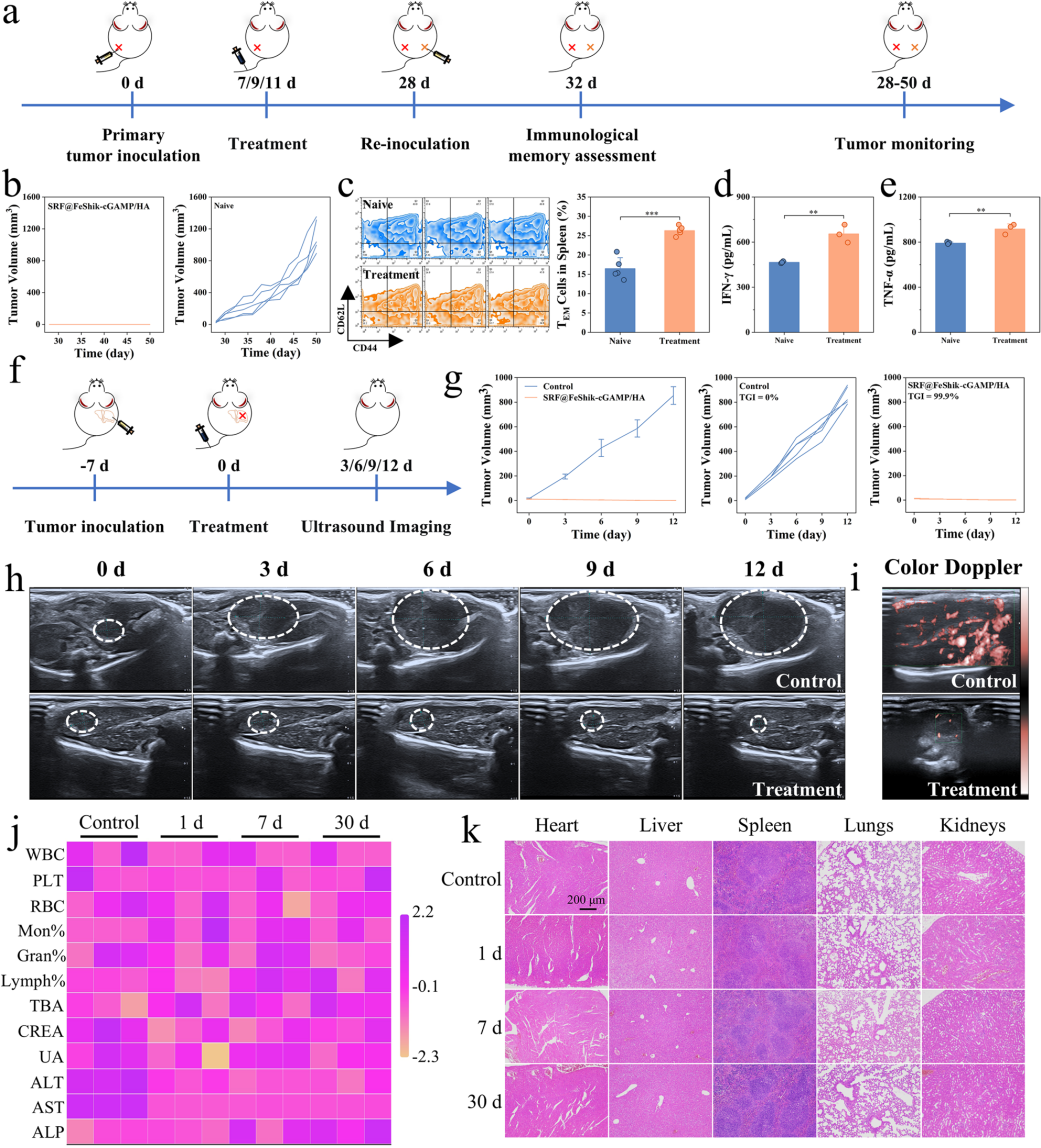
In vivo recurrence, orthotopic and safety studies. **a** Schematic illustration of the treatment protocol in tumor recurrence model. **b** Individual growth curves of re-challenged tumors in SRF@FeShik-cGAMP/HA-treated mice and naive mice (*n* = 5). **c** Representative flow cytometric plots and quantitative analysis of T_EM_ cells (CD3^+^CD8^+^CD44^+^CD62L^-^) in spleen in naive mice and SRF@FeShik-cGAMP/HA-treated mice before Hepa1-6 cell reinoculation (*n* = 5). IFN-γ (**d**) and TNF-α (**e**) levels in serum isolated from naive mice and SRF@FeShik-cGAMP/HA-treated mice after Hepa1-6 cell reinoculation (*n* = 3). **f** Schematic diagram of real-time ultrasound imaging for monitoring tumor treatment efficacy on orthotopic HCC model. **g** Tumor volume changes in the two groups (*n* = 5). **h** Ultrasound real-time imaging shows the size of the orthotopic HCC in Control mice and SRF@FeShik-cGAMP/HA-treated mice. **i** Color doppler flow image of orthotopic HCC. **j** Blood examination results from mice intravenously injected with the SRF@FeShik-cGAMP/HA at different time points.** k** H&E staining images of the major organs

Following the observation of significant immunostimulatory effects and impressive tumor suppression by the SRF@FeShik-cGAMP/HA nanovaccines in primary HCC models, an investigation into the lasting immune memory is also conducted using a tumor recurrence model (Fig. 7a). We investigate the anti-recurrence effect of SRF@FeShik-cGAMP/HA. Hepa1-6 tumor-bearing mice are re-inoculated with Hepa1-6 cells after SRF@FeShik-cGAMP/HA treatment. Furthermore, naive C57BL/6J mice that are matched for age and sex are injected with an equivalent quantity of Hepa1-6 cells. Consistent with our hypothesis, the SRF@FeShik-cGAMP/HA-treated mice completely reject the reinoculated Hepa1-6 cells (Fig. 7b). In sharp contrast, Hepa1-6 tumors grow rapidly in the naive mice without any inhibition. The findings suggest that our SRF@FeShik-cGAMP/HA nanovaccines create a robust antitumor immune memory, resulting in prolonged defense against the recurrence of tumors. To verify the mechanisms underlying the durable immune response induced by SRF@FeShik-cGAMP/HA, we profile effector memory T (T_EM_, CD3^+^CD8^+^CD44^+^CD62^−^) cells in the spleen after SRF@FeShik-cGAMP/HA treatment. Surprisingly, SRF@FeShik-cGAMP/HA (26.3 ± 1.2%) increases the percentage of T_EM_ cells in mice compared with that in naive mice (16.5 ± 2.8%) (Fig. 7c). Additionally, SRF@FeShik-cGAMP/HA-treated mice exhibit significantly increased levels of IFN-γ and TNF-α, which are generated by T_EM_ cells following a subsequent exposure to the same antigen after the reinoculation of Hepa1-6 cells (Fig. 7d and e). These findings suggest that the SRF@FeShik-cGAMP/HA can generate long-term immune memory and stimulate robust immune reactions to inhibit tumor recurrence. Consequently, the activation of dual ferroptosis and necroptosis and the STING pathway by SRF@FeShik-cGAMP/HA not only effectively triggers a systemic antitumor immune response but also promotes long-term immune memory protection.

### Real-time ultrasound imaging on orthotopic HCC model

In the field of modern clinical medicine, ultrasonic diagnostic technology emerges as an indispensable core tool in the prognostic evaluation system for HCC, owing to its unique advantages of real-time dynamic imaging, non-invasiveness, no ionizing radiation hazards, and high convenience and efficiency [53]. Its clinical value is not only reflected in the early screening stage of the disease—where it accurately detects tiny HCC lesions, clearly defines the tumor’s boundary, location, and adjacency to surrounding blood vessels, thereby providing critical anatomical basis for the formulation of subsequent treatment plans [54]. Moreover, it runs through the entire treatment process for efficacy monitoring and prognostic evaluation: by dynamically tracking changes in tumor volume, internal blood supply status, and capsule integrity, it provides real-time feedback on the effectiveness of interventional therapy, targeted therapy, and other treatment regimens [55, 56]. This helps clinicians adjust treatment strategies in a timely manner to optimize patient prognosis to the greatest extent [57].

To further verify the in vivo antitumor activity of the SRF@FeShik-cGAMP/HA nanovaccines, we establish a mouse orthotopic HCC model using Hepa1-6 cells (Fig. 7f). This model highly simulates the natural growth microenvironment of human HCC and holds greater clinical transformation significance compared with traditional subcutaneous xenograft tumor models. On this basis, high-frequency ultrasound technology is used for continuous dynamic monitoring of the model mice, and the differences in tumor growth between the SRF@FeShik-cGAMP/HA treatment group and the control group are systematically compared. The monitoring results show that 12 days after treatment, the volume of orthotopic liver cancer lesions in mice of the SRF@FeShik-cGAMP/HA treatment group is significantly lower than that in the control group (0.8 ± 0.3 mm³ VS 854.2 ± 71.6 mm³), with a tumor volume inhibition rate of 99.9% (Fig. 7g and h). Color doppler ultrasound reveal that the blood supply signal within the tumors of the treatment group is significantly weaker compared to the control group (Fig. 7i). In contrast, the tumors in the control group show a trend of continuous and rapid growth. These results not only intuitively confirm the potent inhibitory effect of the SRF@FeShik-cGAMP/HA nanovaccines on orthotopic HCC but also highlight the important supporting value of ultrasonic diagnostic technology in evaluating the in vivo efficacy of novel antitumor drugs.

From a clinical translation perspective, this study builds a bridge between nanovaccines immunotherapy and clinical ultrasound evaluation. On one hand, it confirms that nanovaccines have the potential to be candidate drugs for targeted cancer immunotherapy, providing a new direction to break the bottlenecks of traditional treatments [58, 59]. On the other hand, it further validates that ultrasound technology, as a non-invasive, real-time, and clinically compatible imaging tool, can be effectively used for efficacy monitoring and protocol guidance in cancer immunotherapy [60, 61]. The synergy of this “treatment-evaluation” system lays a foundation for more precise and personalized diagnosis and treatment strategies in the era of HCC immunotherapy, and also paves the way for the subsequent preclinical optimization and ultimate clinical application of this nanovaccines [62].

### Biosafety

It is essential to ensure the biosafety of nanovaccines for their successful translation into clinical applications. Thus, we perform analyses of routine blood and biochemical parameters in mice at various time points (0, 1, 7, and 30 days) following the administration of SRF@FeShik-cGAMP/HA to evaluate the acute (1 day), short-term (7 days), and long-term (30 days) safety of the nanovaccines (Fig. 7j). The findings demonstrate that there are no notable irregularities in the treatment group when contrasted with the control group, suggesting an absence of clear systemic toxicity. Additionally, H&E staining is employed to histopathologically assess the primary organs taken from the mice at different time intervals. The results show no significant differences between the group treated with intravenous SRF@FeShik-cGAMP/HA and the control group (Fig. 7k). Furthermore, we conduct fluorescence staining assays of ROS (Fig. S41) and LPO (Fig. S42) on the major organs extracted from mice to evaluate the oxidative damage to the major organs caused by SRF@FeShik-cGAMP/HA. The ex vivo fluorescence imaging of major organs shows no obvious oxidative damage or off-target oxidative stress in the SRF@FeShik-cGAMP/HA-treated group compared with the control group. These findings highlight the great in vivo biocompatibility of the nanovaccines and demonstrate the great potential of SRF@FeShik-cGAMP/HA for future clinical translation.

## Conclusions

In summary, we have effectively developed a customized nanovaccine (SRF@FeShik-cGAMP/HA) that demonstrates a smart response to particular chemical signals within the TME. This advancement facilitates the simultaneous co-delivery of various components while also triggering the anti-tumor immune response. The combination of ferroptosis and necroptosis significantly enhances the ICD effect, providing sufficient antigen signals and activation signals for DCs. cGAMP directly activates the STING pathway, promoting the secretion of type I interferons and further amplifying the immune activation cascade. Consequently, the number of M1 macrophages within the tumor region increases, while the number of Tregs and M2 macrophages decreases, improving the state of insufficient immune cell infiltration and functional inhibition in the TME. Meanwhile, the infiltration of mature DCs, NK cells and CTLs at the tumor site increases significantly. This leads to the elimination of primary, orthotopic and recurrent HCC tumors while fostering long-lasting antitumor immune memory, which effectively prevents tumor recurrence and prolongs the survival of the mice. Overall, the mechanism of the SRF@FeShik-cGAMP/HA nanovaccines—“TME responsiveness, multi-component synergy, and immune memory induction”—aligns with the clinical demand for “precise, long-acting, and low-toxic” antitumor immunotherapy. This work provides a promising nanovaccine platform and offers an innovative strategy for the management of HCC *via* the utilization of cascade immunotherapy.

Despite the promising therapeutic efficacy demonstrated in this work, certain aspects still require further exploration. While our study employs multiple tumor models including primary, orthotopic, and recurrent HCC models, they still cannot fully represent the complexity of clinical tumor immune microenvironments. In addition, the synergistic mechanism between ferroptosis, necroptosis, and STING pathway remains to be further validated in more clinically relevant settings. In forthcoming investigations, we will elucidate the prospective capacity of our nanovaccines to inhibit the recruitment of diverse immune cell populations, including but not limited to myeloid-derived suppressor cells (MDSCs). Additionally, we will employ more clinically-relevant individualized models such as patient-derived organoids to further elucidate the molecular mechanism.

## Supplementary Information

Supplementary Material 1.

## Data Availability

The datasets used and analysed during the current study are available from the corresponding author on reasonable request.

## References

[1] Dagher RD, Schwab SK, Brookens AD, Posey Jr. Advances in cancer immunotherapies. Cell. 2023;186(8):1814. 10.1016/j.cell.2023.02.039. 37059073 10.1016/j.cell.2023.02.039

[2] Li J, Lu W, Yang Y, Xiang R, Ling Y, Yu C, Zhou Y. Hybrid nanomaterials for cancer immunotherapy. Adv Sci. 2023;10(6):e2204932. 10.1002/advs.202204932. 10.1002/advs.202204932 PMC9951325 36567305

[3] Wang T, Wang D, Yu H, Feng B, Zhou F, Zhang H, et al. A cancer vaccine-mediated postoperative immunotherapy for recurrent and metastatic tumors. Nat Commun. 2018;9(1):1532. 10.1038/s41467-018-03915-4. 29670088 10.1038/s41467-018-03915-4 PMC5906566

[4] Saxena M, van der Burg SH, Melief CJM, Bhardwaj N. Therapeutic cancer vaccines. Nat Rev Cancer. 2021;21(6):360–78. 10.1038/s41568-021-00346-0. 33907315 10.1038/s41568-021-00346-0

[5] Efimova I, Catanzaro E, Van der Meeren L, Turubanova VD, Hammad H, Mishchenko TA, et al. Vaccination with early ferroptotic cancer cells induces efficient antitumor immunity. J Immunother Cancer. 2020;8(2):e001369. 10.1136/jitc-2020-001369. 33188036 10.1136/jitc-2020-001369 PMC7668384

[6] Wang C, Haas MA, Yeo SK, Paul R, Yang F, Vallabhapurapu S, et al. Autophagy mediated lipid catabolism facilitates glioma progression to overcome bioenergetic crisis. Br J Cancer. 2021;124(10):1711–23. 10.1038/s41416-021-01294-0. 33723393 10.1038/s41416-021-01294-0 PMC8110959

[7] Xie L, Wang G, Sang W, Li J, Zhang Z, Li W, et al. Phenolic immunogenic cell death nanoinducer for sensitizing tumor to PD-1 checkpoint blockade immunotherapy. Biomaterials. 2021;269:120638. 10.1016/j.biomaterials.2020.120638. 33421711 10.1016/j.biomaterials.2020.120638

[8] Decout A, Katz JD, Venkatraman S, Ablasser A. The cGAS-STING pathway as a therapeutic target in inflammatory diseases. Nat Rev Immunol. 2021;21(9):548–69. 10.1038/s41577-021-00524-z. 33833439 10.1038/s41577-021-00524-z PMC8029610

[9] Adkins I, Fucikova J, Garg AD, Agostinis P, Špíšek R. Physical modalities inducing immunogenic tumor cell death for cancer immunotherapy. Oncoimmunology. 2015;3(12):e968434. 10.4161/21624011.2014.968434. 25964865 10.4161/21624011.2014.968434 PMC4352954

[10] Su Q, Liu Z, Du R, Chen X, Chen L, Fu Z, et al. Facile preparation of a metal-phenolic network-based lymph node targeting nanovaccine for antitumor immunotherapy. Acta Biomater. 2023;158:510–24. 10.1016/j.actbio.2022.12.066. 36603733 10.1016/j.actbio.2022.12.066

[11] Su X, Wang WJ, Cao Q, Zhang H, Liu B, Ling Y, et al. A carbonic anhydrase IX (CAIX)-anchored rhenium(I) photosensitizer evokes pyroptosis for enhanced anti-tumor immunity. Angew Chem Int Ed Engl. 2022;61(8):e202115800. 10.1002/anie.202115800. 34842317 10.1002/anie.202115800

[12] Tang R, Xu J, Zhang B, Liu J, Liang C, Hua J, et al. Ferroptosis, necroptosis, and pyroptosis in anticancer immunity. J Hematol Oncol. 2020;13(1):110. 10.1186/s13045-020-00946-7. 32778143 10.1186/s13045-020-00946-7 PMC7418434

[13] Yang J, Ma S, Xu R, Wei Y, Zhang J, Zuo T, et al. Smart biomimetic metal organic frameworks based on ROS-ferroptosis-glycolysis regulation for enhanced tumor chemo-immunotherapy. J Control Release. 2021;334:21–33. 10.1016/j.jconrel.2021.04.013. 33872626 10.1016/j.jconrel.2021.04.013

[14] Gao J, Ye T, Miao H, Liu M, Wen L, Tian Y, et al. Antibody-functionalized iron-based nanoplatform for ferroptosis-augmented targeted therapy of HER2-positive breast cancer. Bioact Mater. 2025;52:702–18. 10.1016/j.bioactmat.2025.06.034. 40641578 10.1016/j.bioactmat.2025.06.034 PMC12241818

[15] Chen C, Wang Z, Jia S, Zhang Y, Ji S, Zhao Z, et al. Evoking highly immunogenic ferroptosis aided by intramolecular motion-induced photo-hyperthermia for cancer therapy. Adv Sci. 2022;9(10):e2104885. 10.1002/advs.202104885. 10.1002/advs.202104885 PMC8981454 35132824

[16] Friedmann Angeli JP, Krysko DV, Conrad M. Ferroptosis at the crossroads of cancer-acquired drug resistance and immune evasion. Nat Rev Cancer. 2019;19(7):405–14. 10.1038/s41568-019-0149-1. 31101865 10.1038/s41568-019-0149-1

[17] Yao L, Zhao MM, Luo QW, Zhang YC, Liu TT, Yang Z, et al. Carbon quantum dots-based nanozyme from coffee induces cancer cell ferroptosis to activate antitumor immunity. ACS Nano. 2022;16(6):9228–39. 10.1021/acsnano.2c01619. 35622408 10.1021/acsnano.2c01619

[18] Pasparakis M, Vandenabeele P. Necroptosis and its role in inflammation. Nature. 2015;517(7534):311–20. 10.1038/nature14191. 25592536 10.1038/nature14191

[19] Dannappel M, Vlantis K, Kumari S, Polykratis A, Kim C, Wachsmuth L, et al. RIPK1 maintains epithelial homeostasis by inhibiting apoptosis and necroptosis. Nature. 2014;513(7516):90–4. 10.1038/nature13608. 25132550 10.1038/nature13608 PMC4206266

[20] Kaiser WJ, Upton JW, Long AB, Livingston-Rosanoff D, Daley-Bauer LP, Hakem R, et al. RIP3 mediates the embryonic lethality of caspase-8-deficient mice. Nature. 2011;471(7338):368–72. 10.1038/nature09857. 21368762 10.1038/nature09857 PMC3060292

[21] Rickard JA, O’Donnell JA, Evans JM, Lalaoui N, Poh AR, Rogers T, et al. RIPK1 regulates RIPK3-MLKL-driven systemic inflammation and emergency hematopoiesis. Cell. 2014;157(5):1175–88. 10.1016/j.cell.2014.04.019. 24813849 10.1016/j.cell.2014.04.019

[22] Murai S, Yamaguchi Y, Shirasaki Y, Yamagishi M, Shindo R, Hildebrand JM, et al. A FRET biosensor for necroptosis uncovers two different modes of the release of DAMPs. Nat Commun. 2018;9(1):4457. 10.1038/s41467-018-06985-6. 30367066 10.1038/s41467-018-06985-6 PMC6203740

[23] Wang F, Fan J, Pan W, Liu M, Wang J, Wei X, et al. Probiotic-inspired hybrid nanovesicles for enhancing immune checkpoint therapy efficiency via tumor immune microenvironment modulation. Bioact Mater. 2025;56:197–216. 10.1016/j.bioactmat.2025.10.012. 41146853 10.1016/j.bioactmat.2025.10.012 PMC12554223

[24] DePeaux K, Delgoffe GM. Metabolic barriers to cancer immunotherapy. Nat Rev Immunol. 2021;21(12):785–97. 10.1038/s41577-021-00541-y. 33927375 10.1038/s41577-021-00541-y PMC8553800

[25] Das A, Ali N. Nanovaccine: an emerging strategy. Expert Rev Vaccines. 2021;20(10):1273–90. 10.1080/14760584.2021.1984890. 34550859 10.1080/14760584.2021.1984890

[26] Zhu J, Yuan A, Le Y, Chen X, Guo J, Liu J, et al. Yi-Qi-Jian-Pi-Xiao-Yu formula inhibits cisplatin-induced acute kidney injury through suppressing ferroptosis via STING-NCOA4-mediated ferritinophagy. Phytomedicine. 2024;156189. 10.1016/j.phymed.2024.156189. 10.1016/j.phymed.2024.156189 39515100

[27] Corrales L, Glickman LH, McWhirter SM, Kanne DB, Sivick KE, Katibah GE, et al. Direct activation of STING in the tumor microenvironment leads to potent and systemic tumor regression and immunity. Cell Rep. 2015;11(7):1018–30. 10.1016/j.celrep.2015.04.031. 25959818 10.1016/j.celrep.2015.04.031 PMC4440852

[28] Fuertes MB, Kacha AK, Kline J, Woo SR, Kranz DM, Murphy KM, Gajewski TF. Host type I IFN signals are required for antitumor CD8^+^ T cell responses through CD8α + dendritic cells. J Exp Med. 2011;208:2005–16. 10.1084/jem.20101159. 21930765 10.1084/jem.20101159 PMC3182064

[29] Demaria O, Gassart AD, Coso S, Gestermann N, Domizio JD, Flatz L, et al. STING activation of tumor endothelial cells initiates spontaneous and therapeutic antitumor immunity. Proc Natl Acad Sci U S A. 2015;112(50):15408–13. 10.1073/pnas.1512832112. 26607445 10.1073/pnas.1512832112 PMC4687570

[30] Du JR, Teng DK, Wang Y, Wang Q, Lin YQ, Luo Q, et al. Endogenous H₂O₂ self-replenishment and sustainable cascades enhance the efficacy of sonodynamic therapy. Int J Nanomed. 2023;18:6667–87. 10.2147/IJN.S431221. 10.2147/IJN.S431221 PMC10656771 38026520

[31] Feng W, Shi W, Cui Y, Xu J, Liu S, Gao H, et al. Fe (III)-Shikonin supramolecular nanomedicines as immunogenic cell death stimulants and multifunctional immunoadjuvants for tumor vaccination. Theranostics. 2023;13(15):5266–89. 10.7150/thno.81650. 37908730 10.7150/thno.81650 PMC10614674

[32] Feng W, Shi W, Liu S, Liu H, Liu Y, Ge P, Zhang H. Fe(III)-Shikonin supramolecular nanomedicine for combined therapy of tumor via ferroptosis and necroptosis. Adv Healthc Mater. 2022;11(2):e2101926. 10.1002/adhm.202101926. 34738742 10.1002/adhm.202101926

[33] Feng W, Shi W, Wang Z, Cui Y, Shao X, Liu S, et al. Enhancing tumor therapy of Fe (III)-Shikonin supramolecular nanomedicine via triple ferroptosis amplification. ACS Appl Mater Interfaces. 2022;14(33):37540–52. 10.1021/acsami.2c11130. 35944147 10.1021/acsami.2c11130

[34] Hayashi K, Nikolos F, Lee YC, Jain A, Tsouko E, Gao H, et al. Tipping the immunostimulatory and inhibitory DAMP balance to harness immunogenic cell death. Nat Commun. 2020;11(1):6299. 10.1038/s41467-020-19970-9. 33288764 10.1038/s41467-020-19970-9 PMC7721802

[35] Kroemer G, Galluzzi L, Kepp O, Zitvogel L. Immunogenic cell death in cancer therapy. Annu Rev Immunol. 2013;31:51–72. 10.1146/annurev-immunol-032712-100008. 23157435 10.1146/annurev-immunol-032712-100008

[36] Yan J, Wang G, Xie L, Tian H, Li J, Li B, et al. Engineering radiosensitizer-based metal-phenolic networks potentiate STING pathway activation for advanced radiotherapy. Adv Mater. 2022;34(10):e2105783. 10.1002/adma.202105783. 34964997 10.1002/adma.202105783

[37] Zheng J, Huang J, Zhang L, Wang M, Xu L, Dou X, et al. Drug-loaded microbubble delivery system to enhance PD-L1 blockade immunotherapy with remodeling immune microenvironment. Biomater Res. 2023;27(1):9. 10.1186/s40824-023-00350-5. 36759928 10.1186/s40824-023-00350-5 PMC9909878

[38] Yue W, Chen L, Yu L, Zhou B, Yin H, Ren W, et al. Checkpoint blockade and nanosonosensitizer-augmented noninvasive sonodynamic therapy combination reduces tumour growth and metastases in mice. Nat Commun. 2019;10(1):2025. 10.1038/s41467-019-09760-3. 31048681 10.1038/s41467-019-09760-3 PMC6497709

[39] Yang WS, SriRamaratnam R, Welsch ME, Shimada K, Skouta R, Viswanathan VS, et al. Regulation of ferroptotic cancer cell death by GPX4. Cell. 2014;156(1–2):317–31. 10.1016/j.cell.2013.12.010. 24439385 10.1016/j.cell.2013.12.010 PMC4076414

[40] Zhu P, Pu Y, Wang M, Wu W, Qin H, Shi J. MnOOH-catalyzed autoxidation of glutathione for reactive oxygen species production and nanocatalytic tumor innate immunotherapy. J Am Chem Soc. 2023;145(10):5803–15. 10.1021/jacs.2c12942. 36848658 10.1021/jacs.2c12942

[41] Shi W, Feng W, Li S, Cui Y, Liu S, Jiang H, et al. Ferroptosis and necroptosis produced autologous tumor cell lysates co-delivering with combined immunoadjuvants as personalized in situ nanovaccines for antitumor immunity. ACS Nano. 2023;17(15):14475–93. 10.1021/acsnano.3c00901. 37466500 10.1021/acsnano.3c00901

[42] Crow YJ, Casanova JL. Human life within a narrow range: the lethal ups and downs of type I interferons. Sci Immunol. 2024;9(97):eadm8185. 10.1126/sciimmunol.adm8185. 38968338 10.1126/sciimmunol.adm8185

[43] Shae D, Becker KW, Christov P, Yun DS, Lytton-Jean AKR, Sevimli S, et al. Endosomolytic polymersomes increase the activity of cyclic dinucleotide STING agonists to enhance cancer immunotherapy. Nat Nanotechnol. 2019;14(3):269–78. 10.1038/s41565-018-0342-5. 30664751 10.1038/s41565-018-0342-5 PMC6402974

[44] Marcus AJ, Mao A, Lensink-Vasan M, Wang L, Vance RE, Raulet DH. Tumor-derived cGAMP triggers a STING-mediated interferon response in non-tumor cells to activate the NK cell response. Immunity. 2018;49(4):754–e7634. 10.1016/j.immuni.2018.09.016. 30332631 10.1016/j.immuni.2018.09.016 PMC6488306

[45] Crouse J, Bedenikovic G, Wiesel M, Ibberson M, Xenarios I, Laer DV, Kalinke U, et al. Type I interferons protect T cells from NK cell attack mediated by the activating receptor NCR1. Immunity. 2014;40(6):961–73. 10.1016/j.immuni.2014.05.003. 24909889 10.1016/j.immuni.2014.05.003

[46] Yu R, Zhu B, Chen D. Type I interferon-mediated tumor immunity and its role in immunotherapy. Cell Mol Life Sci. 2022;79(3):e93490. 10.1007/s00018-022-04219-z. 10.1007/s00018-022-04219-z PMC8924142 35292881

[47] Corrales L, Gajewski TF. Molecular pathways: targeting the stimulator of interferon genes (STING) in the immunotherapy of cancer, Clin. Cancer Res. 2015;21(21):4774–9. 10.1158/1078-0432.CCR-15-1362. 10.1158/1078-0432.CCR-15-1362 PMC4750108 26373573

[48] Huang Z, Brodeur KE, Chen L, Du Y, Wobma H, Hsu EE, et al. Type I interferon signature and cycling lymphocytes in macrophage activation syndrome. J Clin Invest. 2023;133(22):e165616. 10.1172/JCI165616. 37751296 10.1172/JCI165616 PMC10645381

[49] Diamond MS, Kinder M, Matsushita H, Mashayekhi M, Dunn GP, Archambault JM, et al. Type I interferon is selectively required by dendritic cells for immune rejection of tumors. J Exp Med. 2011;208(10):1989–2003. 10.1084/jem.20101158. 21930769 10.1084/jem.20101158 PMC3182061

[50] Tian X, Ai J, Tian X, Wei X. cGAS-STING pathway agonists are promising vaccine adjuvants. Med Res Rev. 2024;44(4):1768–99. 10.1002/med.22016. 38323921 10.1002/med.22016

[51] Woo SR, Fuertes MB, Corrales L, Spranger S, Furdyna MJ, Leung MY, et al. STING-dependent cytosolic DNA sensing mediates innate immune recognition of immunogenic tumors. Immunity. 2014;41(5):830–42. 10.1016/j.immuni.2014.10.017. 25517615 10.1016/j.immuni.2014.10.017 PMC4384884

[52] Woo SR, Corrales L, Gajewski TF. The STING pathway and the T cell-inflamed tumor microenvironment. Trends Immunol. 2015;36(4):250–6. 10.1016/j.it.2015.02.003. 25758021 10.1016/j.it.2015.02.003 PMC4393801

[53] Dietrich CF, Nolsøe CP, Barr RG, Berzigotti A, Burns PN, Cantisani V, et al. Guidelines and Good Clinical Practice Recommendations for Contrast Enhanced Ultrasound (CEUS) in the Liver - Update 2020 - WFUMB in Cooperation with EFSUMB, AFSUMB, AIUM, and FLAUS. Ultraschall Med. 2020;41(5):562–85. 10.1055/a-1177-0530. 32707595 10.1055/a-1177-0530

[54] Marrero JA, Kulik LM, Sirlin CB, Zhu AX, Finn RS, Abecassis MM, et al. Diagnosis, staging, and management of hepatocellular carcinoma: 2018 practice guidance by the American Association for the Study of Liver Diseases. Hepatology. 2018;68(2):723–50. 10.1002/hep.29913. 29624699 10.1002/hep.29913

[55] Lencioni R, Llovet JM. Modified RECIST (mRECIST) assessment for hepatocellular carcinoma. Semin Liver Dis. 2010;30(1):52–60. 10.1055/s-0030-1247132. 20175033 10.1055/s-0030-1247132 PMC12268942

[56] Chen J, Zhu J, Zhang C, Song Y, Huang P. Contrast-enhanced ultrasound for the characterization of portal vein thrombosis vs tumor-in-vein in HCC patients: a systematic review and meta-analysis. Eur Radiol. 2020;30(5):2871–80. 10.1007/s00330-019-06649-z. 32020403 10.1007/s00330-019-06649-z PMC7160216

[57] Cheng MQ, Huang H, Ruan SM, Xu P, Tong WJ, He DN. Complementary Role of CEUS and CT/MR LI-RADS for Diagnosis of Recurrent HCC. Cancers. 2023;15(24):5743. 10.3390/cancers15245743. 38136289 10.3390/cancers15245743 PMC10741803

[58] Riley RS, June CH, Langer R, Mitchell MJ. Delivery technologies for cancer immunotherapy. Nat Rev Drug Discov. 2019;18(3):175–96. 10.1038/s41573-018-0006-z. 30622344 10.1038/s41573-018-0006-z PMC6410566

[59] Sahin U, Türeci Ö. Personalized vaccines for cancer immunotherapy. Science. 2018;359(6382):1355–60. 10.1126/science.aar7112. 29567706 10.1126/science.aar7112

[60] Lassau N, Koscielny S, Albiges L, Chami L, Benatsou B, Chebil M, et al. Metastatic renal cell carcinoma treated with sunitinib: early evaluation of treatment response using dynamic contrast-enhanced ultrasonography, Clin. Cancer Res. 2010;16(4):1216–25. 10.1158/1078-0432.CCR-09-2175. 10.1158/1078-0432.CCR-09-2175 20145174

[61] Sidhu PS, Cantisani V, Dietrich CF, Gilja OH, Saftoiu A, Bartels E, et al. The EFSUMB guidelines and recommendations for the clinical practice of contrast-enhanced ultrasound (CEUS) in non-hepatic applications. Ultraschall Med. 2018;39(2):e2–44. 10.1055/a-0586-1107. 29510439 10.1055/a-0586-1107

[62] Chen Q, Wang C, Zhang X, Chen G, Hu Q, Li H, et al. In situ sprayed bioresponsive immunotherapeutic gel for post-surgical cancer treatment. Nat Nanotechnol. 2019;14(1):89–97. 10.1038/s41565-018-0319-4. 30531990 10.1038/s41565-018-0319-4

