# Correction: Nanovaccines with ferroptosis, necroptosis and STING-activation for synergistic immunotherapy

**DOI:** 10.1186/s13046-026-03770-y

**Published:** 2026-07-02

**Authors:** Jia-Rui Du, Mu-Le Tu, Yong-Xu Xia, Yuan-Qiang Lin, Rui Yang, Chang Weng, Yi Liu, Hao Zhang, Hui Wang, Wen-Jie Feng, Deng-Ke Teng

**Affiliations:** 1 Department of Ultrasound, China-Japan Union Hospital of Jilin University, Changchun, 130033 P.R. China; 2 Institute of Translational Medicine, The First Hospital of Jilin University, Changchun, 130021 P. R. China; 3 3Department of Ultrasound, The Third Affiliated Clinical Hospital of Changchun University of Chinese Medicine, Changchun, 130117 P. R. China; 4 State Key Laboratory of Supramolecular Structure and MaterialsCollege of Chemistry, Jilin University, Changchun, 130012 P. R. China

**Correction: J Exp Clin Cancer Res 45, 132 (2026)**

**https://doi.org/10.1186/s13046-026-03726-2**

Following publication of the original article [1], the authors spotted errors caused by journal typesetting during the production stage, which were not present in the original manuscript submitted by the authors. Specifically, the figure content designated for Fig. 4 was incorrectly arranged as Fig. 2 leading to duplicate Fig. 2; two textual errors in the main text were also introduced by layout editing. The details are given below:

1. In page 3 - “Fe” should be corrected to “Fe^2+^”.
2. In page 8 - “1 × 10” should be corrected to “1 × 10^6^”.

The corrections do not compromise the validity of the conclusions and the overall content of the article. The author group has been updated above and the original article [1] has been corrected.

**Incorrect Fig. 4**

**Fig. 4:**
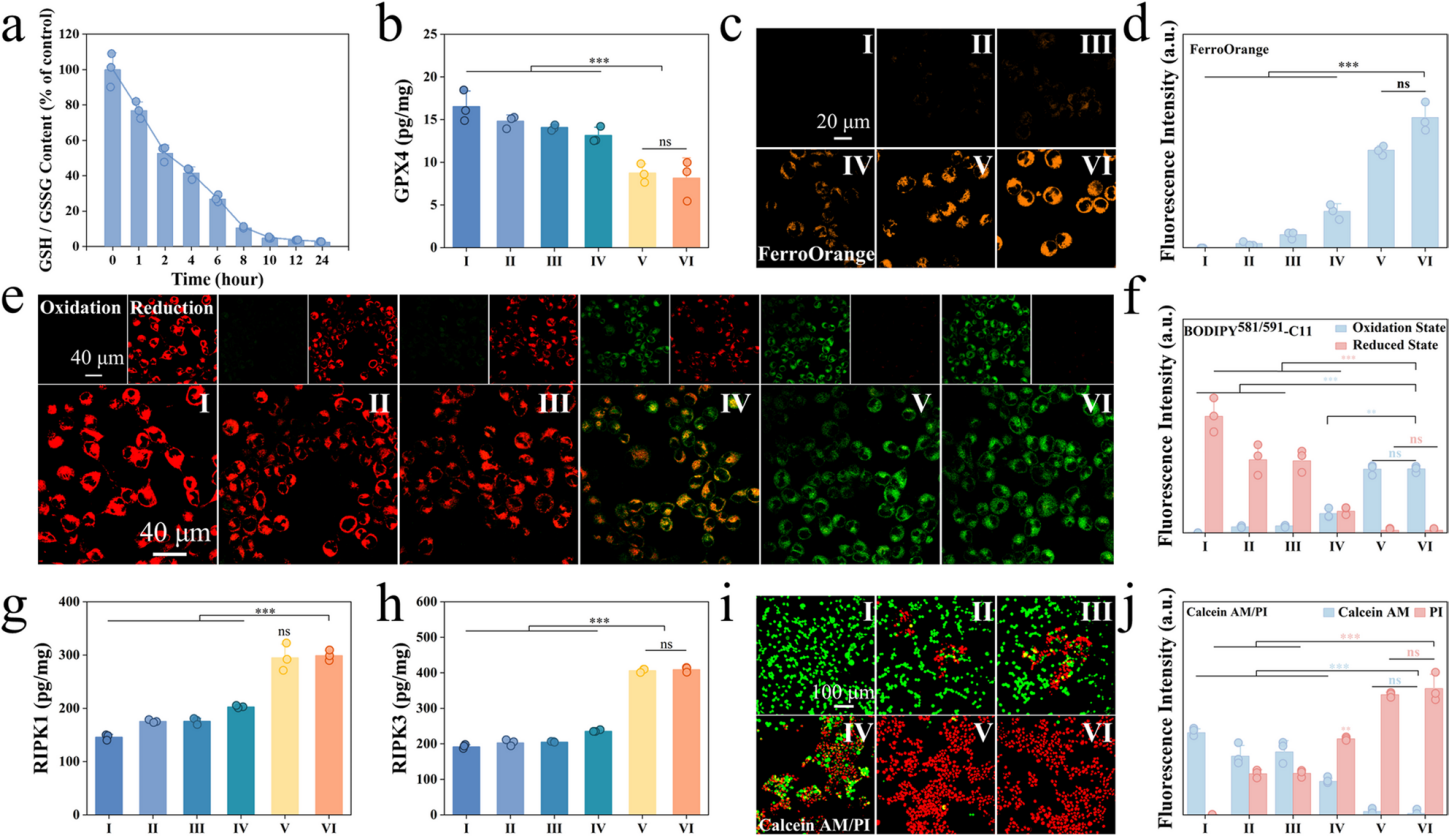
In vitro immune response study. CLSM images of HMGB1 release (**a**) and CRT exposure (**b**) in Hepa1-6 cells after different treatments. Quantification of HMGB1 (**c**) and CRT (**d**) expression levels after different treatments by ELISA (*n* = 3). **e** Quantification of ATP secretion levels in cell medium after different treatments by ATP assay kit (*n* = 3). Quantification of IFN-β (**f**) and CXCL10 (**g**) expression levels after different treatments by ELISA (*n* = 3). **h** Representative flow cytometric plots of DC maturation (CD11c+CD80 + CD86+, gated on CD11c+ cells) after different treatments. Quantification of IL-6 (**i**) and TNF-α (**j**) expression levels after different treatments by ELISA (*n* = 3). **k** Flow cytometric results of the expression of CD86 (M1 macrophages) and CD206 (M2 macrophages) after treatment with different formulations. Levels of IL-12 (**l**) and IL-10 (**m**) secreted by macrophages in the different treatment groups (*n* = 3). **n** Quantitative determination of H_2_O_2_ production in the different groups (*n* = 3). Groups in a-j: (I) Control, (II) SRF, (III) FeShik, (IV) SRF@FeShik, (V) SRF@FeShik-HA, (VI) SRF@FeShik-cGAMP/HA. Groups in k-n: (I) M0, (II) M2, (III) SRF@FeShik + M2, (IV) SRF@FeShik-HA + M2, (V) SRF@FeShik-cGAMP/HA + M2

**Correct Fig. 4**

**Fig. 4:**
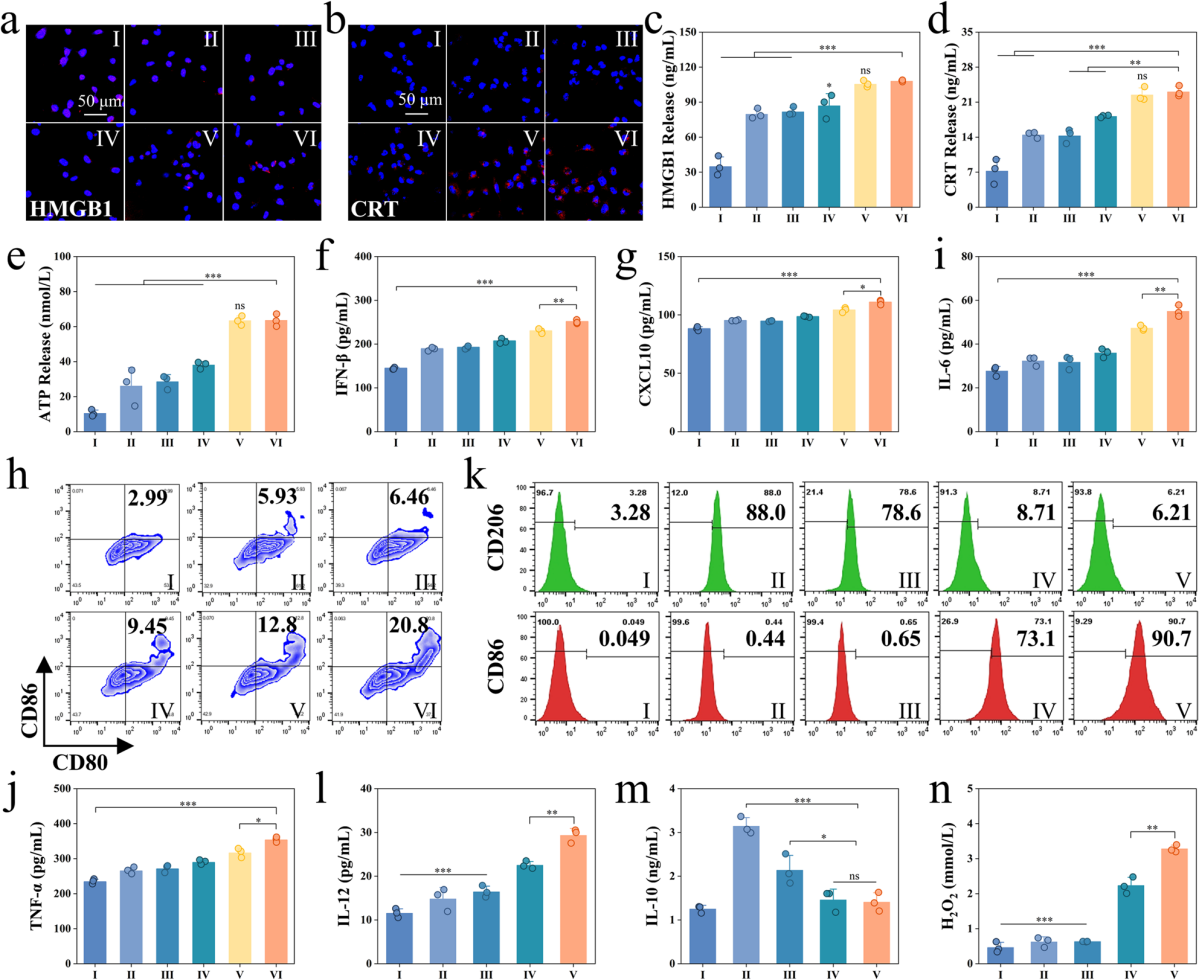
In vitro immune response study. CLSM images of HMGB1 release (**a**) and CRT exposure (**b**) in Hepa1-6 cells after different treatments. Quantification of HMGB1 (**c**) and CRT (**d**) expression levels after different treatments by ELISA (n = 3). **e** Quantification of ATP secretion levels in cell medium after different treatments by ATP assay kit (n = 3). Quantification of IFN-β (**f**) and CXCL10 (**g**) expression levels after different treatments by ELISA (n = 3). **h** Representative flow cytometric plots of DC maturation (CD11c+CD80 + CD86+, gated on CD11c+ cells) after different treatments. Quantification of IL-6 (**i**) and TNF-α (**j**) expression levels after different treatments by ELISA (n = 3). **k** Flow cytometric results of the expression of CD86 (M1 macrophages) and CD206 (M2 macrophages) after treatment with different formulations. Levels of IL-12 (**l**) and IL-10 (**m**) secreted by macrophages in the different treatment groups (n = 3). **n** Quantitative determination of H2O2 production in the different groups (n = 3). Groups in a-j: (I) Control, (II) SRF, (III) FeShik, (IV) SRF@FeShik, (V) SRF@FeShik-HA, (VI) SRF@FeShik-cGAMP/HA. Groups in k-n: (I) M0, (II) M2, (III) SRF@FeShik + M2, (IV) SRF@FeShik-HA + M2, (V) SRF@FeShik-cGAMP/HA + M2.
