# Supplementary material for: Nanovaccines with ferroptosis, necroptosis and STING-activation for synergistic immunotherapy
Source: J Exp Clin Cancer Res. 2026 May 11;45:132. doi: 10.1186/s13046-026-03726-2 (PMC13255514; doi:10.1186/s13046-026-03726-2)

Supporting Information

Nanovaccines with Ferroptosis, Necroptosis and STING-Activation for Synergistic Immunotherapy

Jia-Rui Du[1], Mu-Le Tu[3], Yong-Xu Xia[1], Yuan-Qiang Lin[1], Rui Yang[4], Chang Weng[4], Yi Liu[2,4], Hao Zhang[2,4], Hui Wang[1], Wen-Jie Feng[2,*], Deng-Ke Teng[1,*]

[1]Department of Ultrasound, China-Japan Union Hospital of Jilin University, Changchun 130033, P. R. China

[2]Institute of Translational Medicine, The First Hospital of Jilin University, Changchun 130021, P. R. China

[3]Department of Ultrasound, The Third Affiliated Clinical Hospital of Changchun University of Chinese Medicine, Changchun 130117, P. R. China

[4]State Key Laboratory of Supramolecular Structure and Materials, College of Chemistry, Jilin University, Changchun 130012, P. R. China

[*]**Corresponding Authors**

Wen-Jie Feng - Institute of Translational Medicine, The First Hospital of Jilin University, Changchun 130021, P. R. China, Email: wenjiefeng@jlu.edu.cn.

Deng-Ke Teng - Department of Ultrasound, China-Japan Union Hospital of Jilin University, Changchun 130033, P. R. China, orcid.org/0000-0003-4266-8941; Email: tengdk@jlu.edu.cn.

[*]These authors contributed equally: Wen-Jie Feng and Deng-Ke Teng.

Figure S1. TEM size distribution of SRF@FeShik.

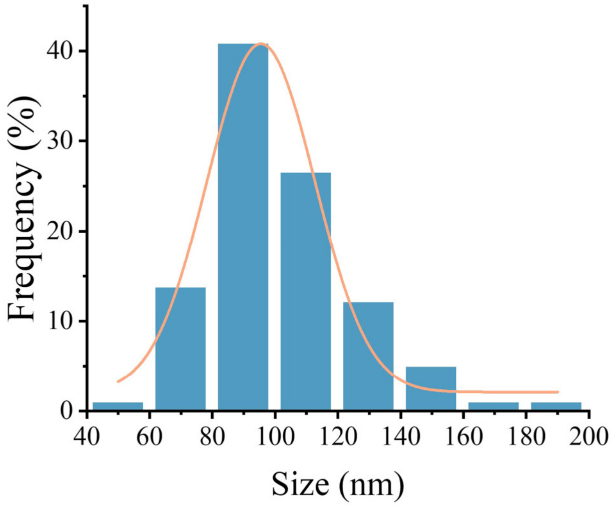

Figure S2. TEM size distribution of SRF@FeShik-cGAMP/HA.

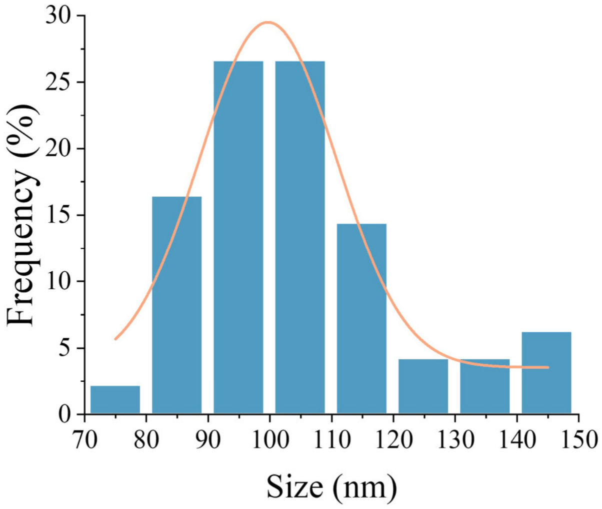

Figure S3. Hydrodynamic diameter (a-c), polydispersity index (PDI) (d-f), and zeta potential (g-i) of SRF@FeShik and SRF@FeShik-cGAMP/HA stored in H_2O, PBS (pH 7.4) or serum-containing media over a 7-day period (n = 3).

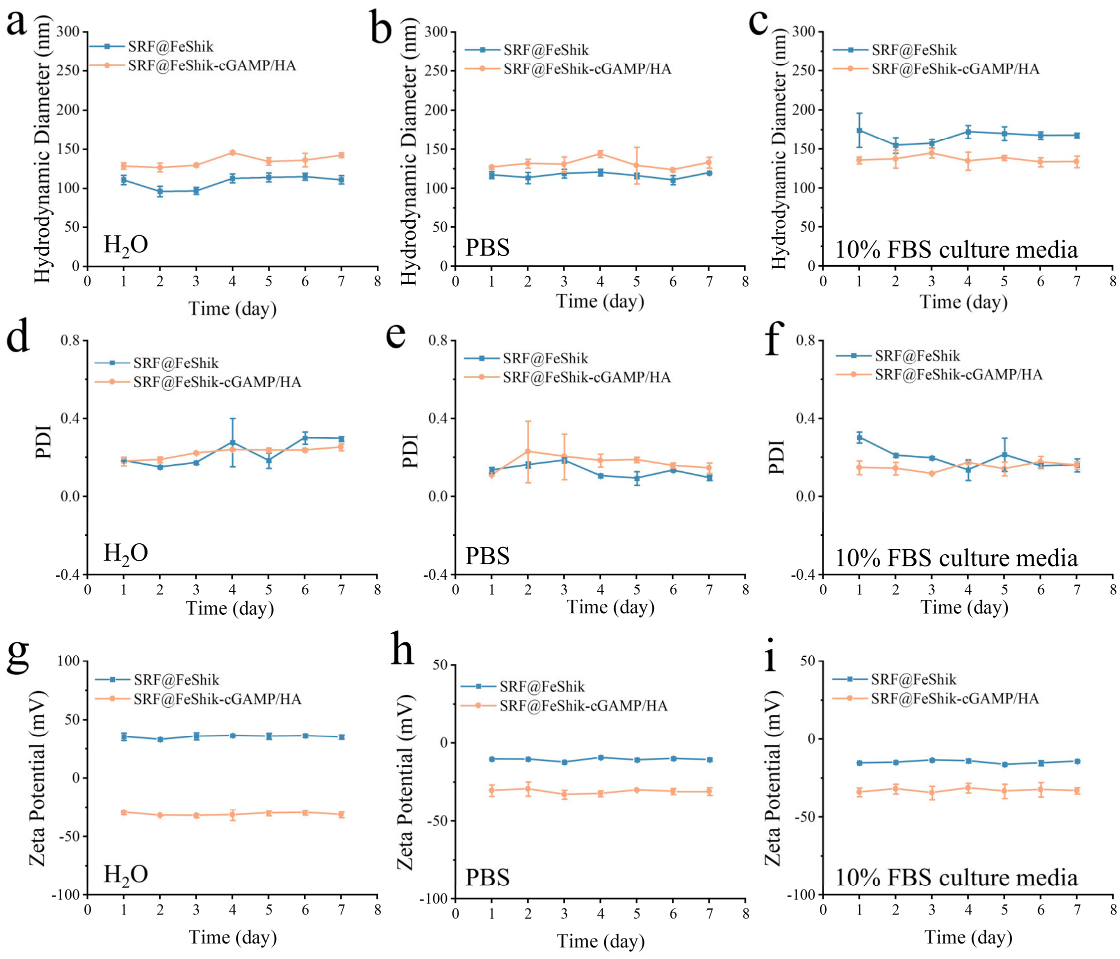

Figure S4. TEM images and corresponding photographs (a), hydrated diameters (b), and UV-vis absorption spectra (c) of solution when nanovaccines are treated with interaction-disrupting agents.

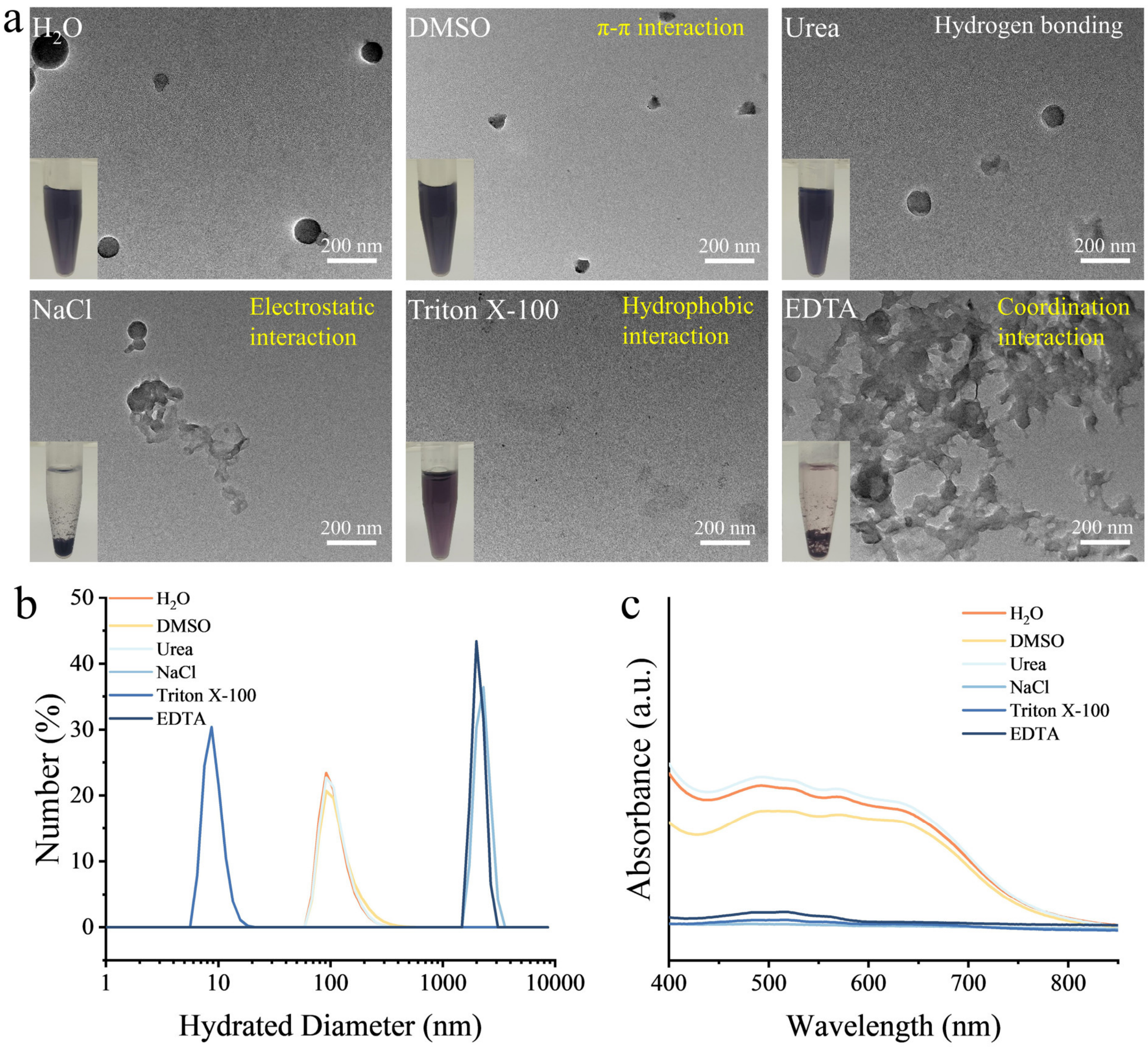

Figure S5. TEM images of nanovaccines incubated with GSH at 0 min, 15 min, 1 h, and 12 h.

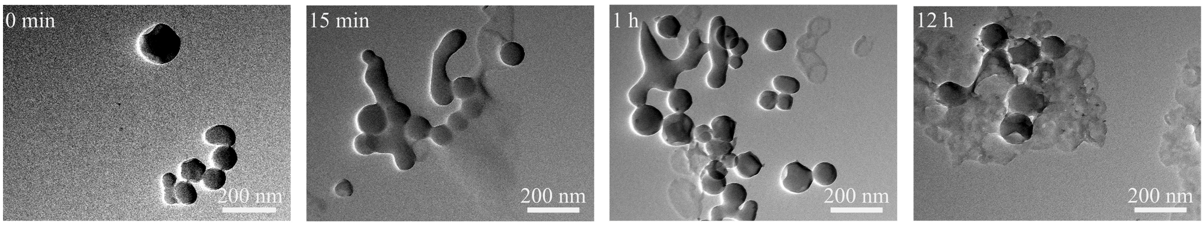

Figure S6. (a) UV-vis absorption spectra of Fe^{2+} at varying concentrations. (b) Fe^{2+} standard calibration curve.

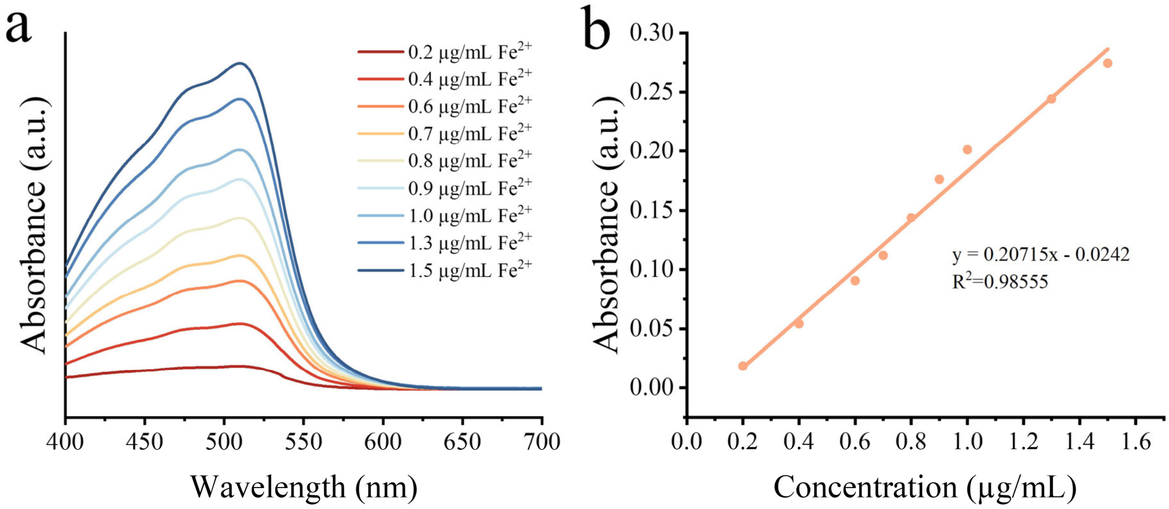

Figure S7. (a) UV-vis absorption spectra of shikonin at varying concentrations. (b) Shikonin standard calibration curve.

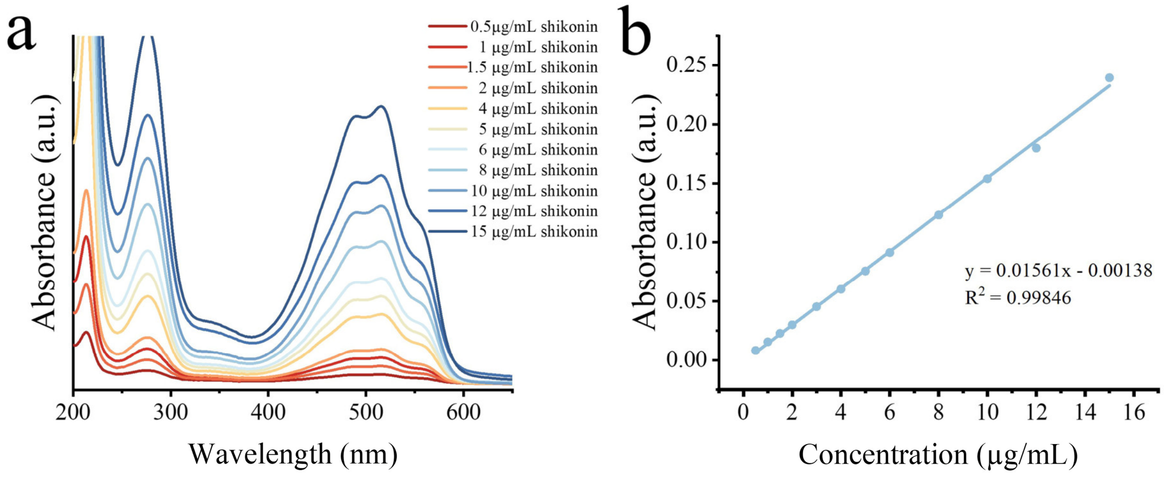

Figure S8. ·OH generation monitored by MB degradation using UV-vis spectroscopy.

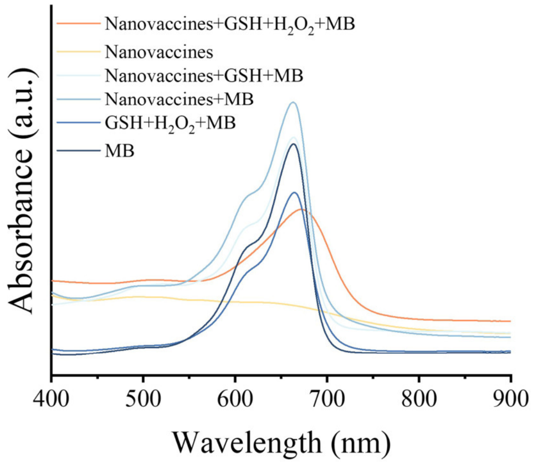

Figure S9. Accumulative SRF (a), Fe^{3+} (b), and shikonin (c) release of nanovaccines at pH 7.4, 6.5, and 5.5 (n = 3).

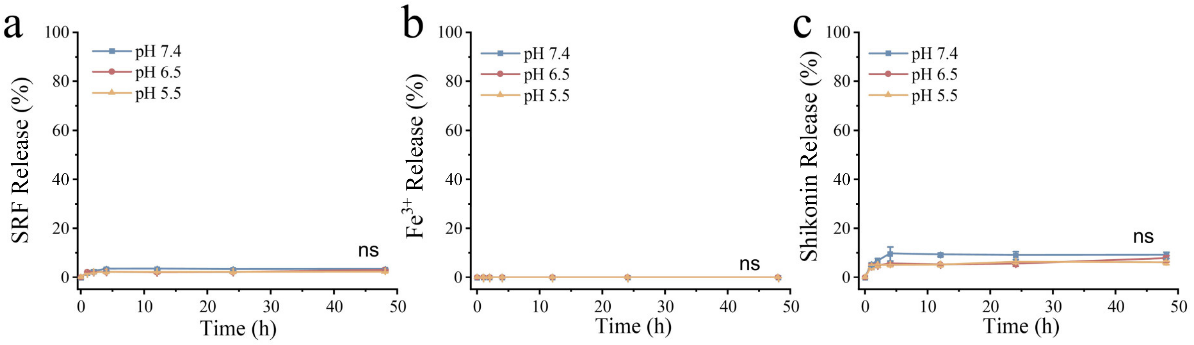

Figure S10. (a) UV-vis absorption spectra of SRF at varying concentrations. (b) SRF standard calibration curve.

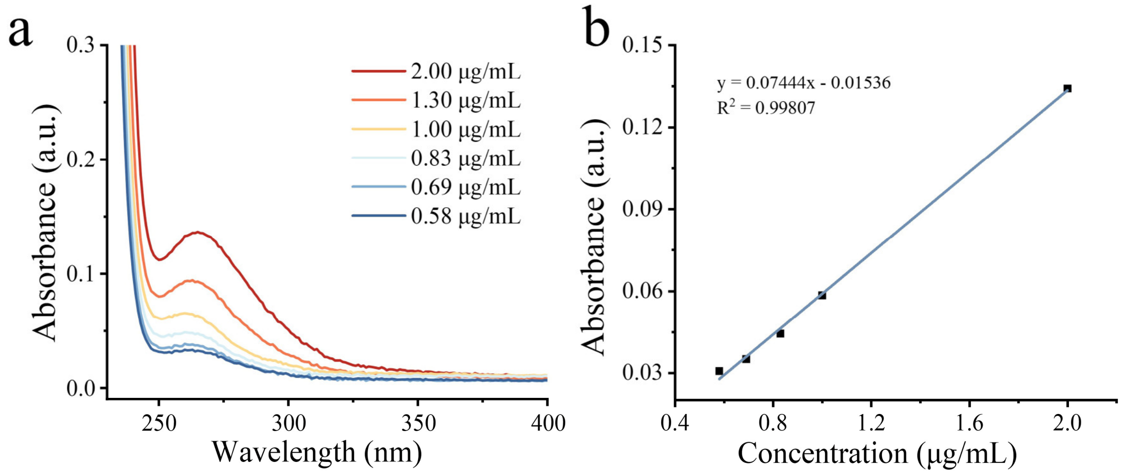

Figure S11. (a) UV-vis absorption spectra of Fe^{3+} at varying concentrations. (b) Fe^{3+} standard calibration curve.

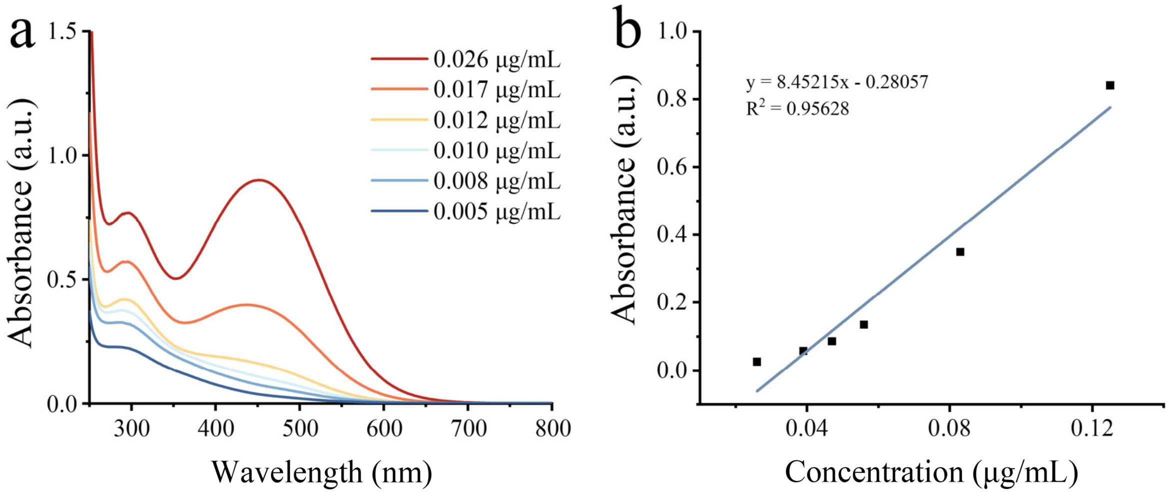

Figure S12. (a) Cell viability of Hepa1-6 cells after treatment with different concentrations of SRF@FeShik-cGAMP/HA (n = 3). (b) Cell viability of HUVECs after treatment with different concentrations of SRF@FeShik-cGAMP/HA (n = 3).

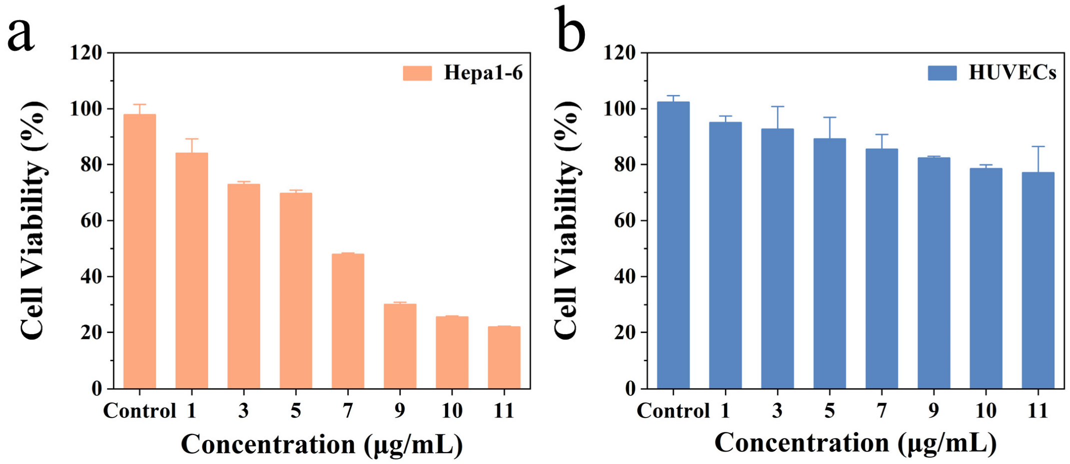

Figure S13. Fluorescence spectra of FITC, ^{FITC}SRF@FeShik-cGAMP, and ^{FITC}SRF@FeShik-cGAMP/HA.

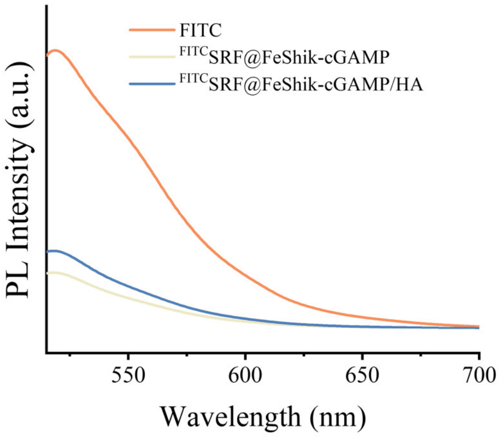

Figure S14. Flow cytometry results (a) and corresponding quantification (b) of Hepa1-6 cells in different groups (*n* = 3). (c) CLSM images of Hepa1-6 cells incubated with different groups. (d) Quantitative analysis of fluorescence intensity measured by ImageJ (*n* = 3). Groups: (I) ^{FITC}SRF@FeShik-cGAMP, (II) HA + ^{FITC}SRF@FeShik-cGAMP/HA, (III) ^{FITC}SRF@FeShik-cGAMP/HA

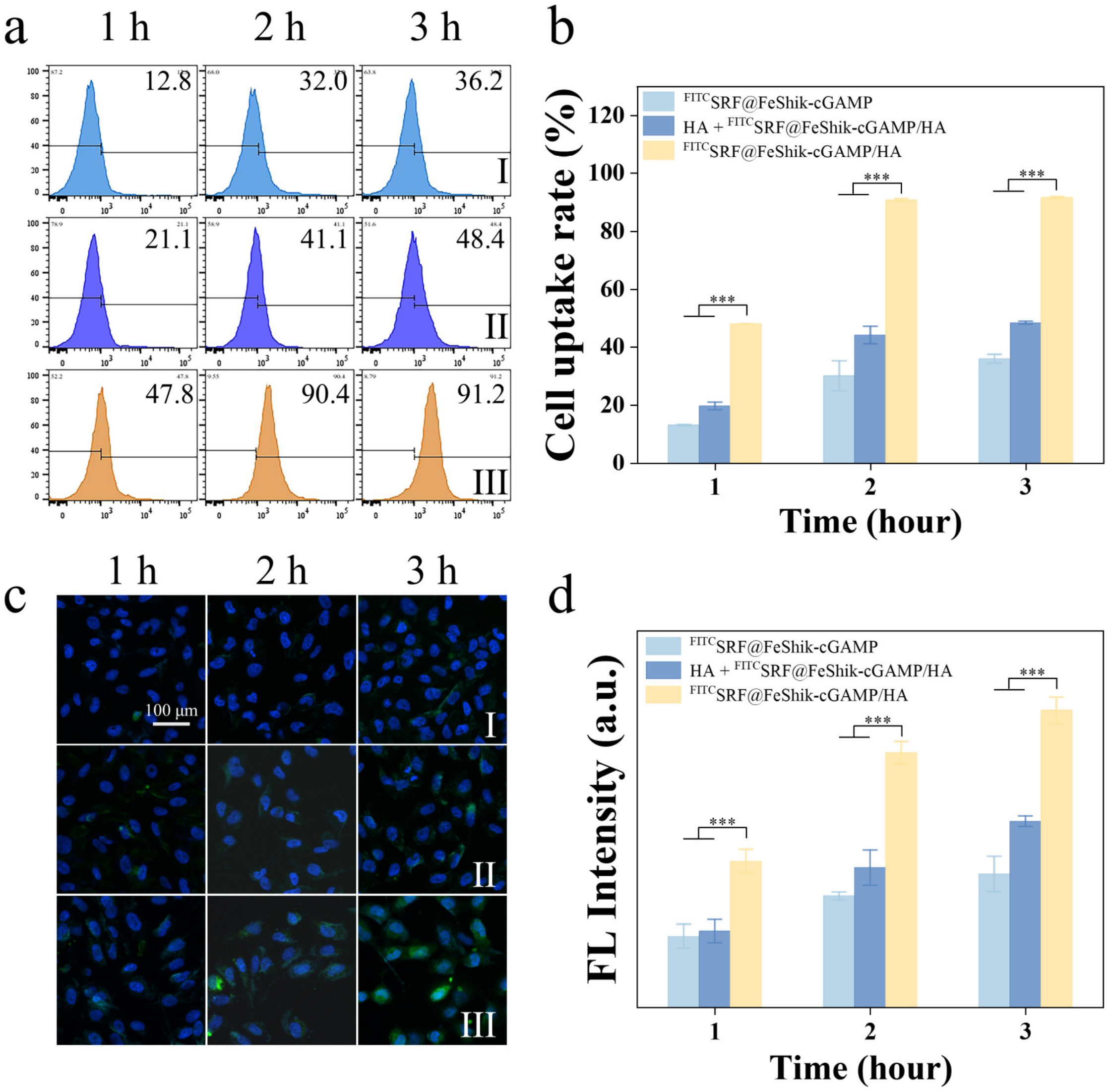

Figure S15. (a) CLSM images of intracellular ROS in Hepa1-6 cells in the different groups. (b) Flow cytometry results of Hepa1-6 cells treated with different groups. Groups: (I) PBS, (II) SRF, (III) FeShik, (IV) SRF@FeShik, (V) SRF@FeShik-HA, (VI) SRF@FeShik-cGAMP/HA.

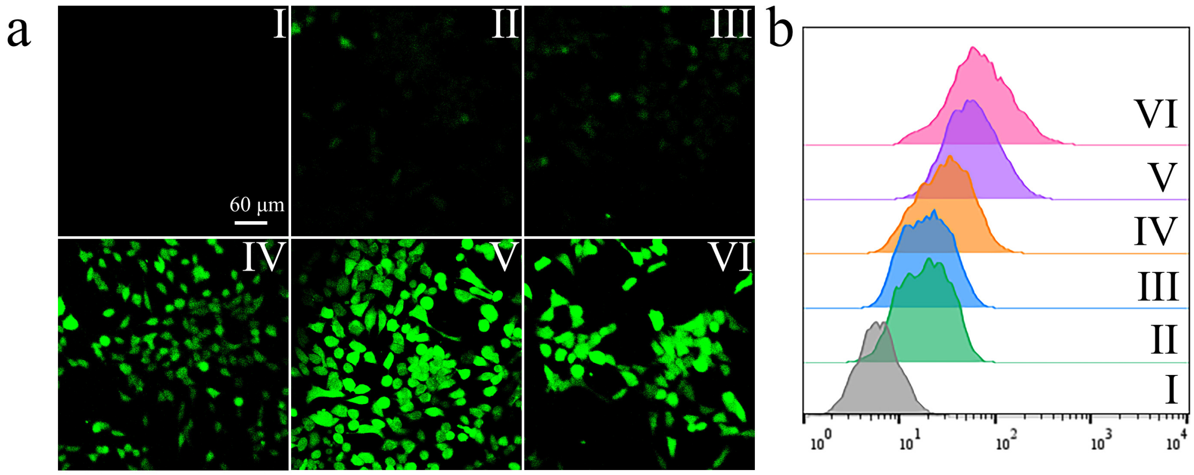

Figure S16. Heat-map analysis of mRNA expression levels of genes involved in ROS.

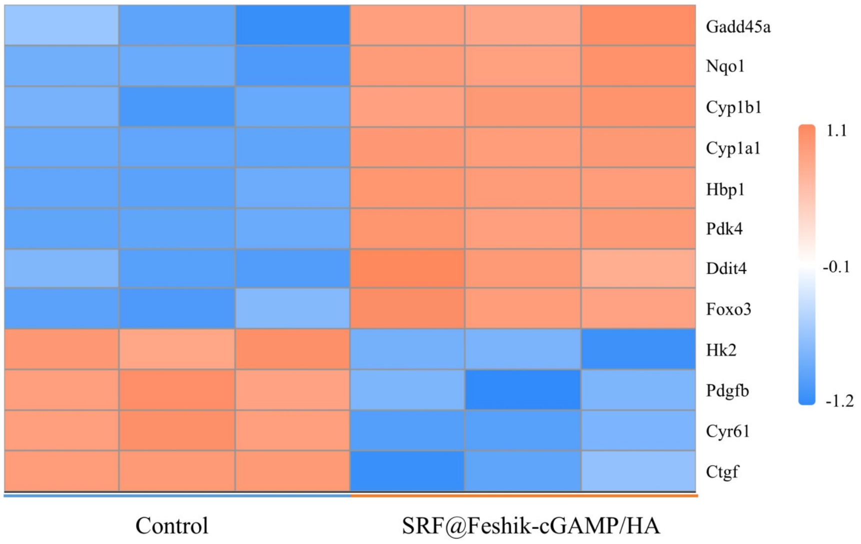

Figure S17. Cell viability of SRF@FeShik-cGAMP/HA treated Hepa1-6 cells after the addition of Fer-1, Nec-1, VC, VE and DFO (n = 3).

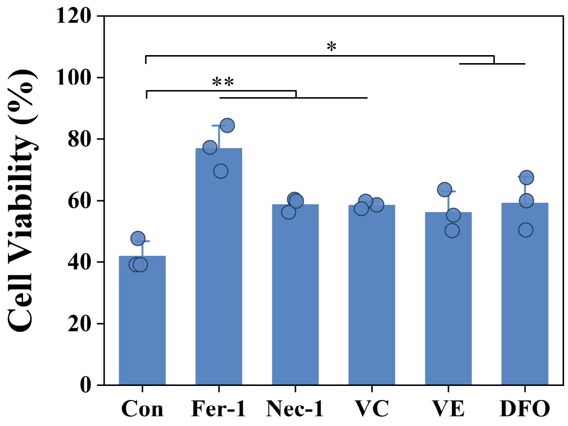

Figure S18. Relative GSH content (a), GSSG content (b), and GSH/GSSG ratio (c) of Hepa1-6 cells at different treatments (*n* = 3). Groups: (I) Control, (II) SRF, (III) FeShik, (IV) SRF@FeShik, (V) SRF@FeShik-HA, (VI) SRF@FeShik-cGAMP/HA.

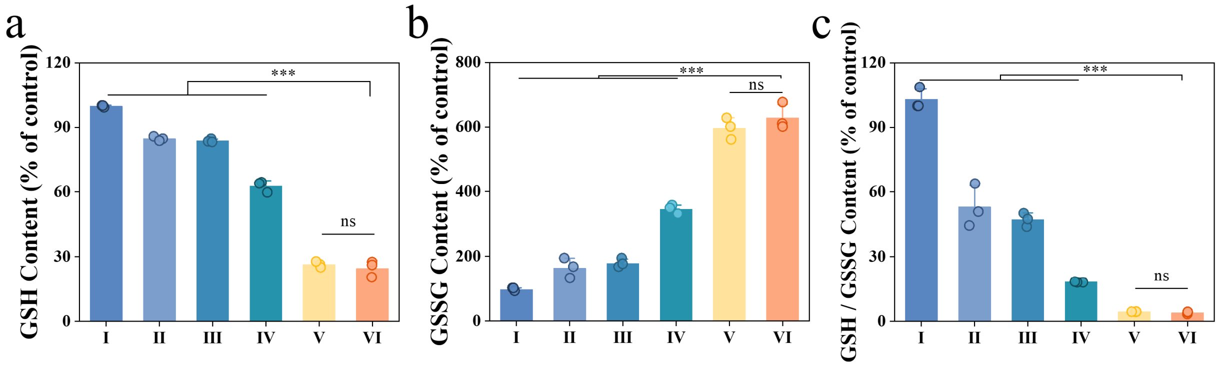

Figure S19. (a) GSEA analysis and (b) heat-map analysis of differentially expressed genes of GSH metabolism.

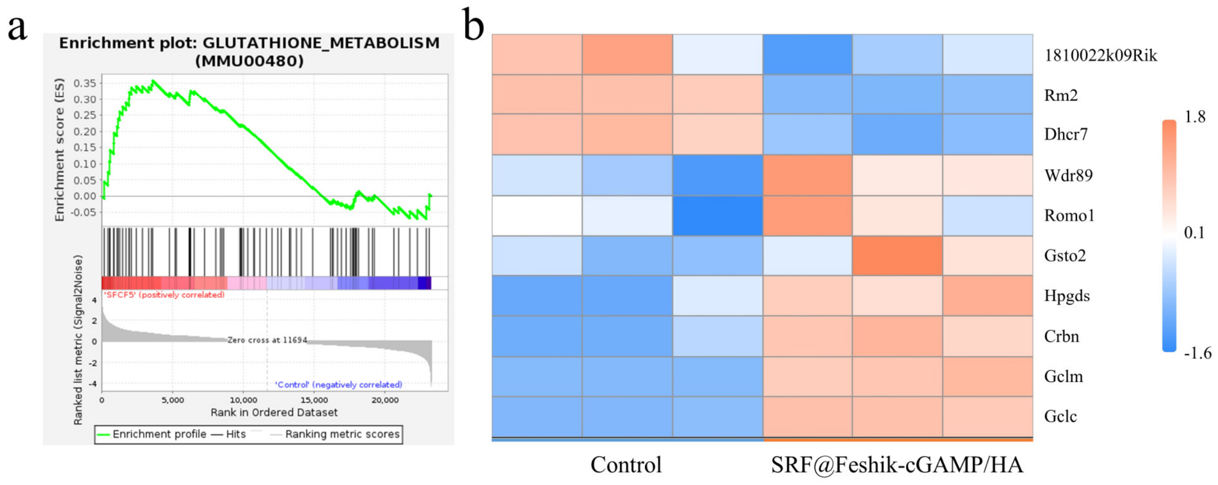

Figure S20. Relative lipid peroxidation MDA of Hepa1-6 cells treated with different groups (*n* = 3). Groups: (I) PBS, (II) SRF, (III) FeShik, (IV) SRF@FeShik, (V) SRF@FeShik-HA, (VI) SRF@FeShik-cGAMP/HA.

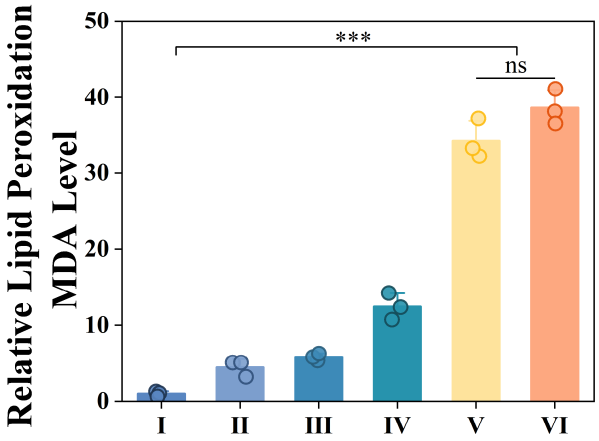

Figure S21. (a) Representative CLSM images showing the mitochondrial membrane potentials of JC-1 stained Hepa1-6 cells in different groups. Red fluorescence: JC-1 Aggregates, green fluorescence: JC-1 Monomers. (b) Quantitative analysis of fluorescence intensity ($n = 3$). Flow cytometry results (c) and corresponding quantification (d) of Hepa1-6 cells in different groups ($n = 3$). Groups: (I) PBS, (II) SRF, (III) FeShik, (IV) SRF@FeShik, (V) SRF@FeShik-HA, (VI) SRF@FeShik-cGAMP/HA.

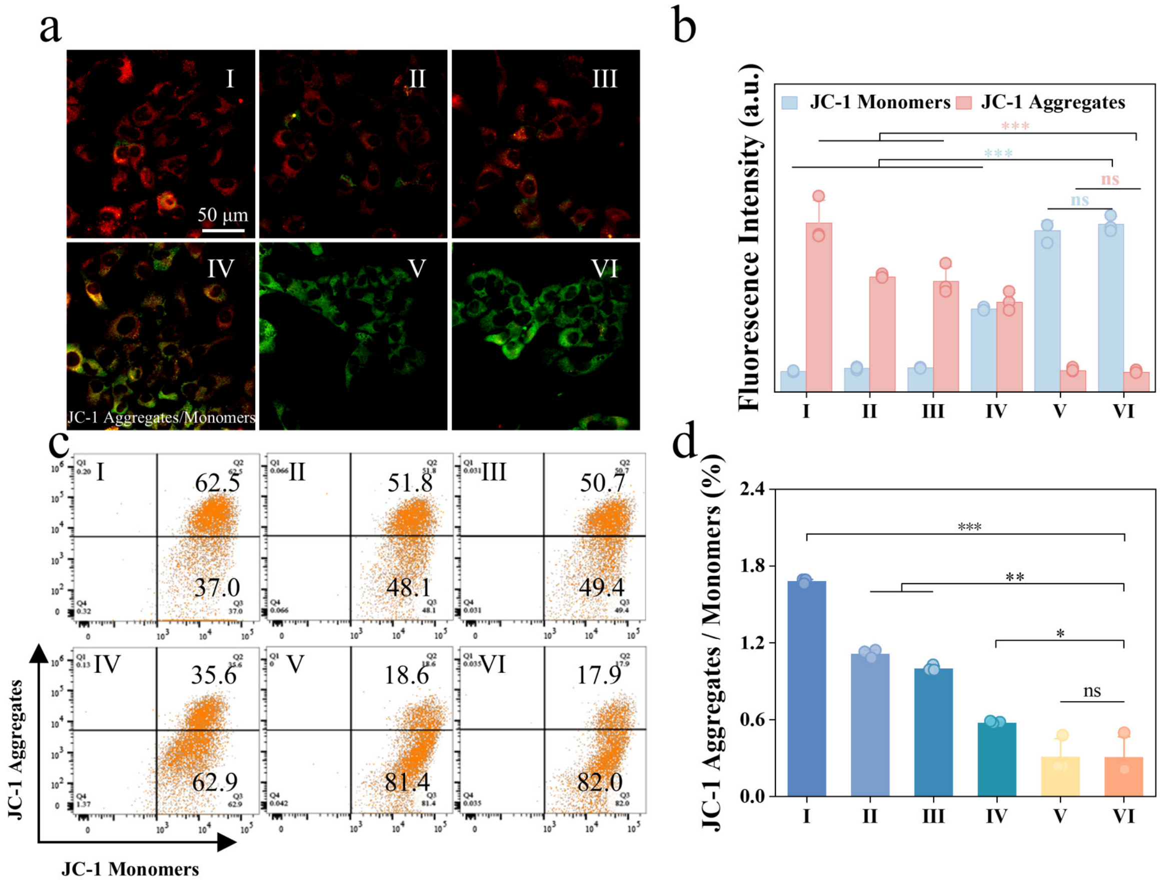

Figure S22. (a) Principal component analysis. (b) KEGG and Reactome enrichment analysis showing the differentially expressed genes between the control and SRF@FeShik-cGAMP/HA.

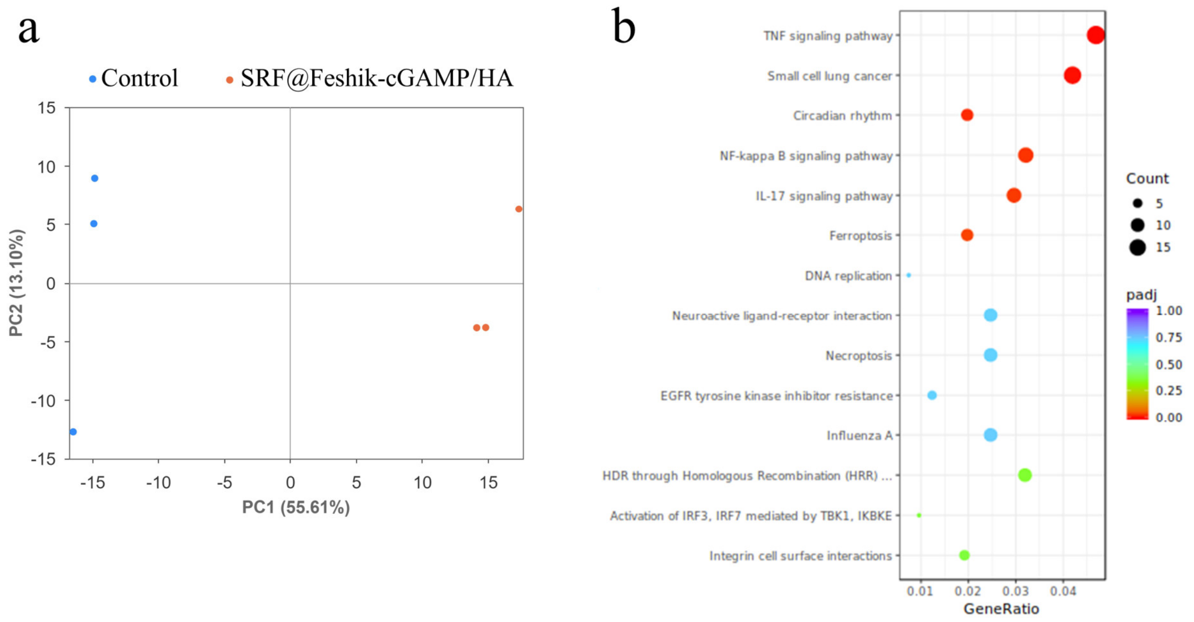

Figure S23. Protein-protein interaction network from the STRING database.

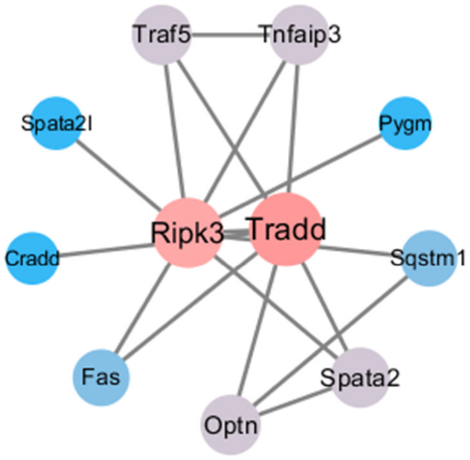

Necroptosis

Figure S24. CLSM images of p-STING expression in Hepa1-6 cells after different treatments. Groups: (I) PBS, (II) SRF, (III) FeShik, (IV) SRF@FeShik, (V) SRF@FeShik-HA, (VI) SRF@FeShik-cGAMP/HA.

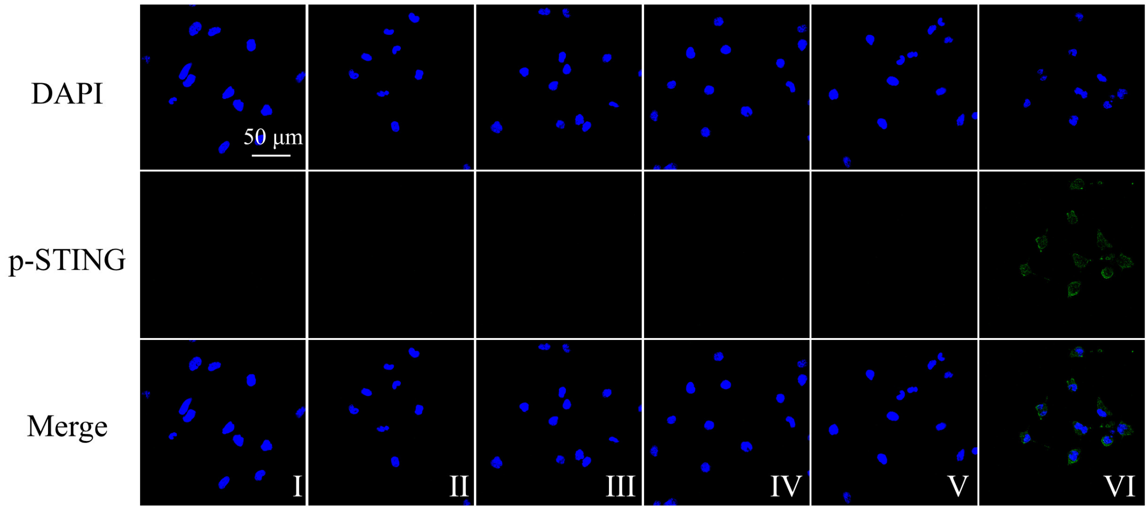

Figure S25. Blood circulation profiles of ^{IR780}SRF@FeShik-cGAMP (a) and ^{IR780}SRF@FeShik-cGAMP/HA (b) in mice by recording the IR780 fluorescence intensity of blood samples at different time points (n = 3).

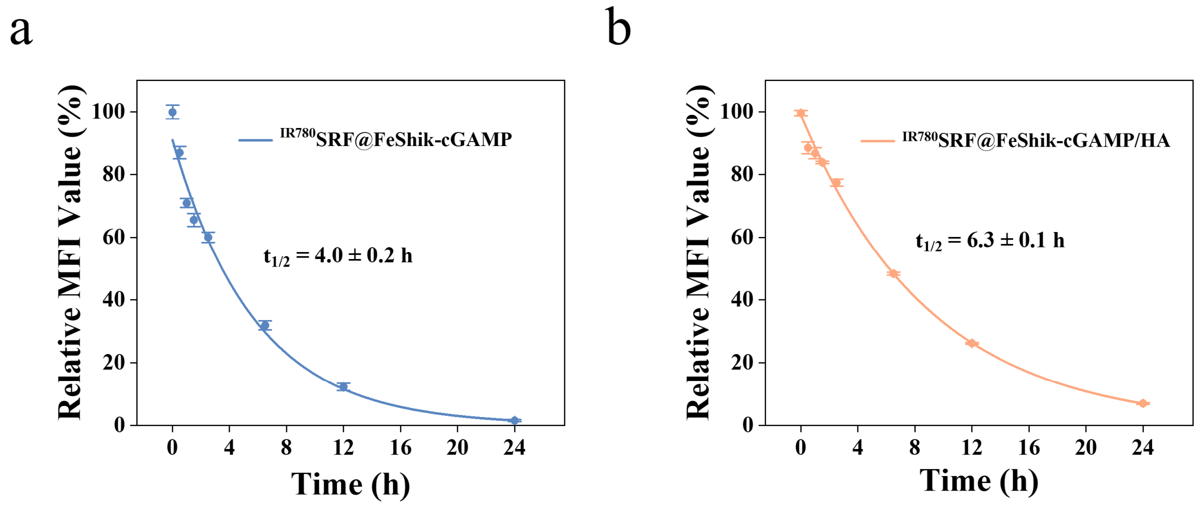

Figure S26. (a) *In vivo* fluorescence images of Hepa1-6 tumor-bearing mice after intravenous injection of ^{IR780}SRF@FeShik-cGAMP or ^{IR780}SRF@FeShik-cGAMP/HA for different times and *ex vivo* fluorescence image of tumor and major organs dissected from mice after intravenous injection for 48 h. Groups: (I) ^{IR780}SRF@FeShik-cGAMP, (II) ^{IR780}SRF@FeShik-cGAMP/HA. (b) Accumulation curves of ^{IR780}SRF@FeShik-cGAMP or ^{IR780}SRF@FeShik-cGAMP/HA in tumor tissues by measuring the fluorescence intensity of tumors at different time points (n = 3). (c) Quantification analysis of ^{IR780}SRF@FeShik-cGAMP or ^{IR780}SRF@FeShik-cGAMP/HA in tumor and major organs by testing the corresponding fluorescence intensity after intravenous injection for 48 h (n = 3).

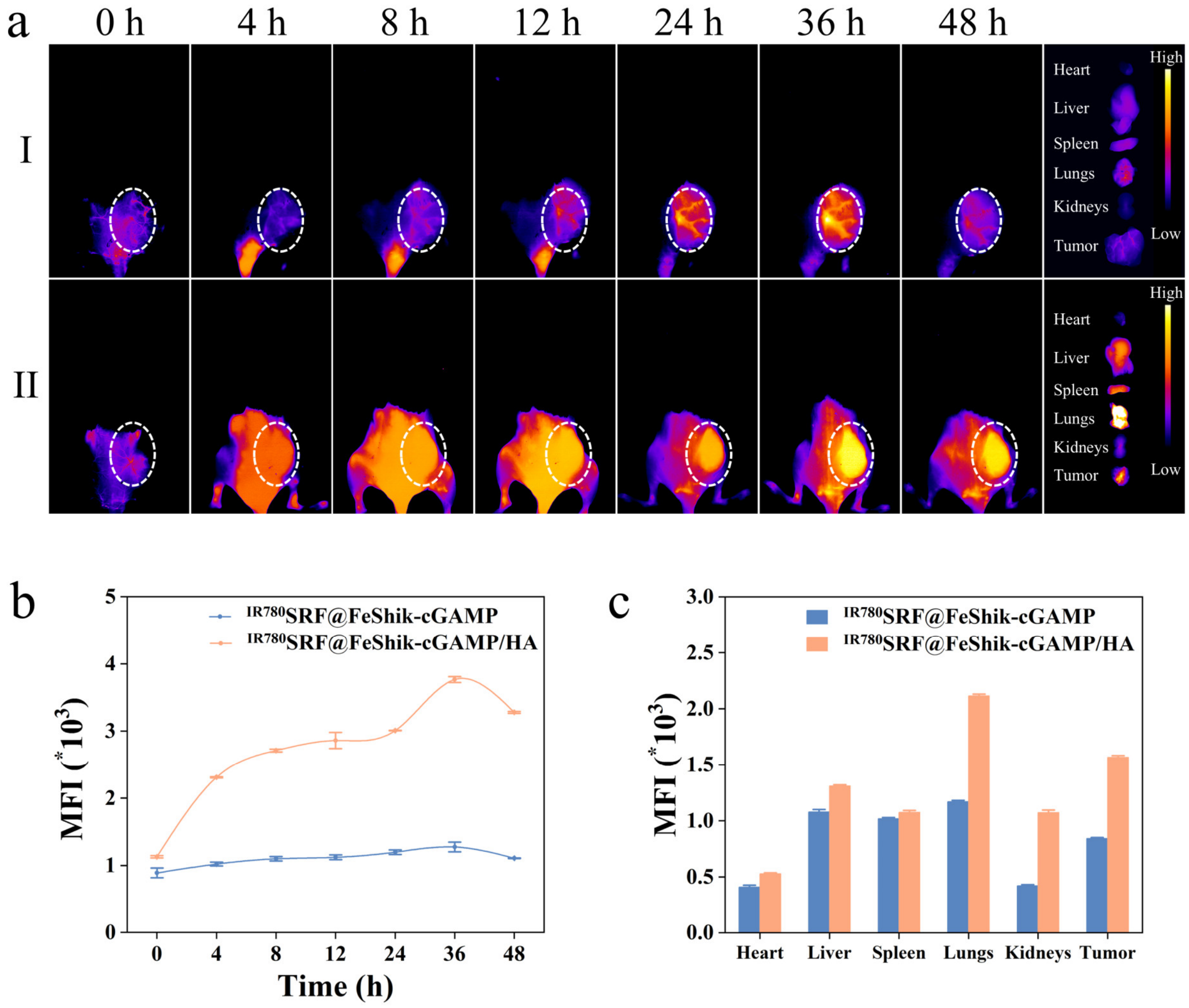

Figure S27. The blood circulation curve after intravenous injection of SRF@FeShik-cGAMP (a) and SRF@FeShik-cGAMP/HA (b) (n = 3). The biodistribution of Fe (% injected dose (ID) of Fe per gram of tissue) in main tissues at different time points after intravenous injection of SRF@FeShik-cGAMP (c) and SRF@FeShik-cGAMP/HA (d) (n = 3).

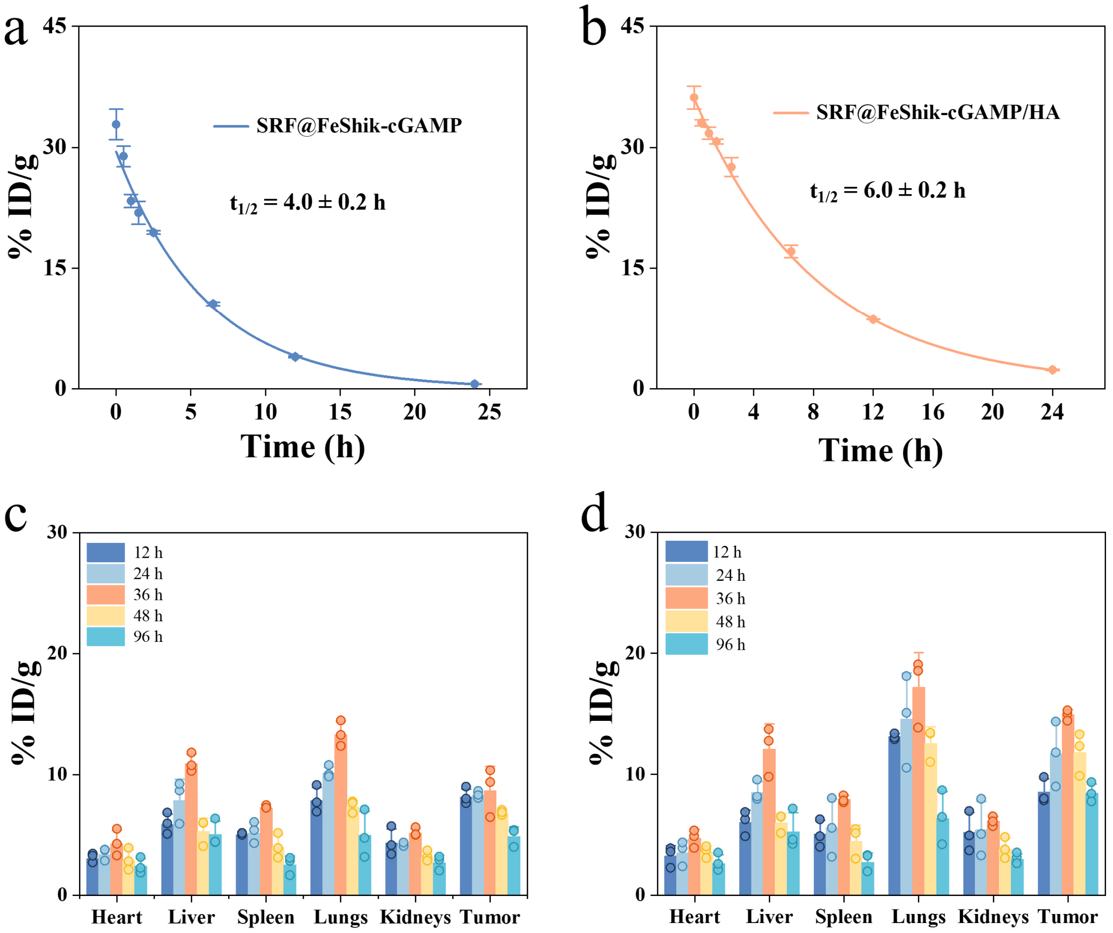

Table S1. Pharmacokinetic parameters of Fe in plasma (n = 3).

Parameters	SRF@FeShik-cGAMP	SRF@FeShik-cGAMP/HA	Unit
Cmax	82.2 ± 4.7	90.5 ± 3.5	μg/L
AUC (0-t)	468.5 ± 6.4	759.0 ± 6.3	μg/L*h
AUC (0-∞)	475.9 ± 6.0	807.8 ± 11.8	μg/L*h
$t½$	4.0 ± 0.2	6.0 ± 0.2	h
Clearance	21.0 ± 0.3	12.4 ± 0.2	L/h/kg

Figure S28. H&E staining of tumor tissue after different treatments. Groups: (I) PBS, (II) SRF, (III) FeShik, (IV) SRF@FeShik, (V) SRF@FeShik-HA, (VI) SRF@FeShik-cGAMP/HA.

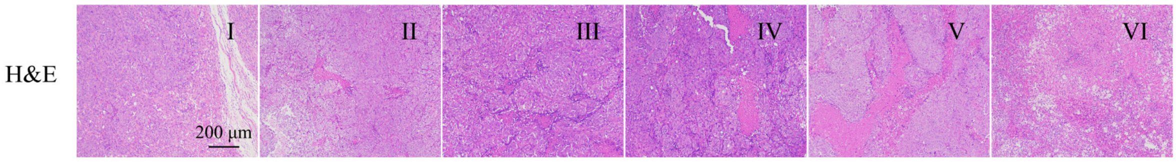

Figure S29. (a) Immunofluorescence images of RIPK1 and RIPK3 in tumors. Fluorescence intensity of RIPK1 (b) and RIPK3 (c) immunofluorescence images measured by ImageJ (n = 3). Groups: (I) PBS, (II) SRF, (III) FeShik, (IV) SRF@FeShik, (V) SRF@FeShik-HA, (VI) SRF@FeShik-cGAMP/HA.

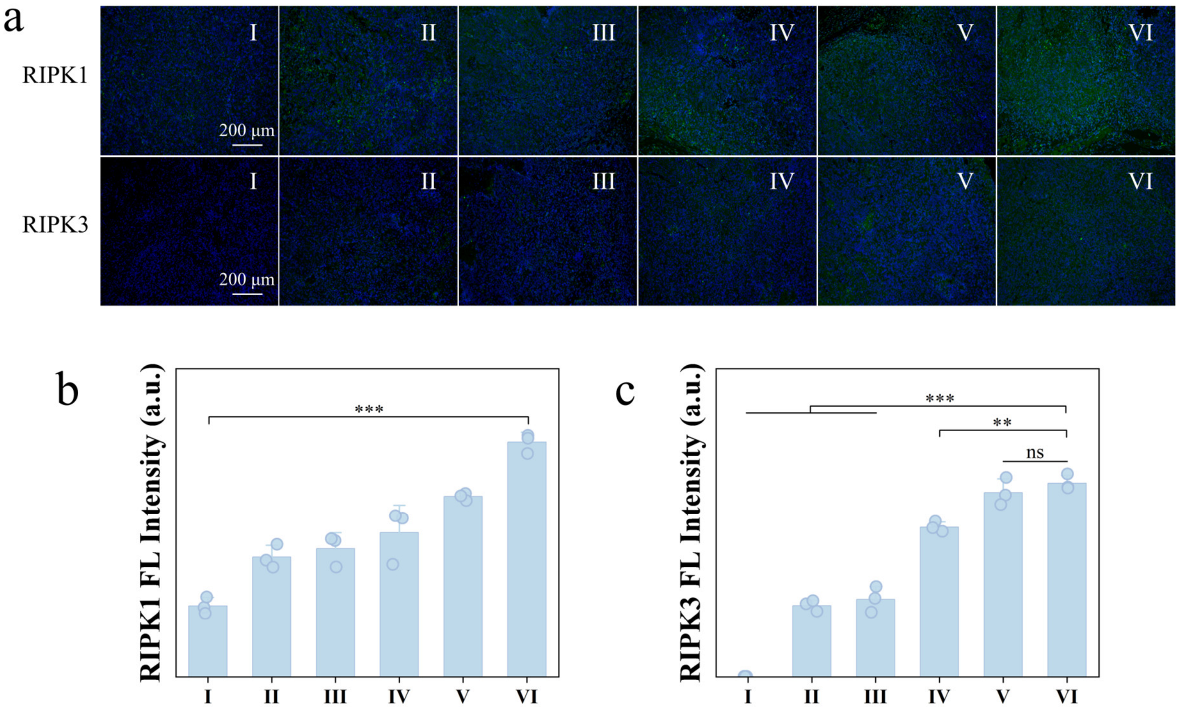

Figure S30. (a) Immunofluorescence images of GPX4 in tumors. (b) Fluorescence intensity of GPX4 immunofluorescence images measured by ImageJ (n = 3). Groups: (I) PBS, (II) SRF, (III) FeShik, (IV) SRF@FeShik, (V) SRF@FeShik-HA, (VI) SRF@FeShik-cGAMP/HA.

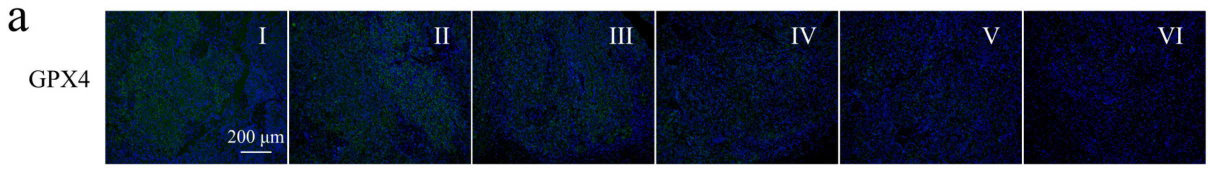

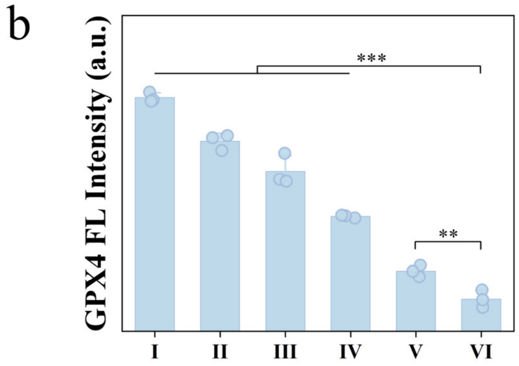

Figure S31. (a) Fluorescence staining images of DHE (pseudocolor: green) and BODIPY$^{581/591}$-C11 (red fluorescence: reduced state) in tumors. Fluorescence intensity of DHE (b) and BODIPY$^{581/591}$-C11 (c) fluorescence images measured by ImageJ (n = 3). Groups: (I) PBS, (II) SRF, (III) FeShik, (IV) SRF@FeShik, (V) SRF@FeShik-HA, (VI) SRF@FeShik-cGAMP/HA.

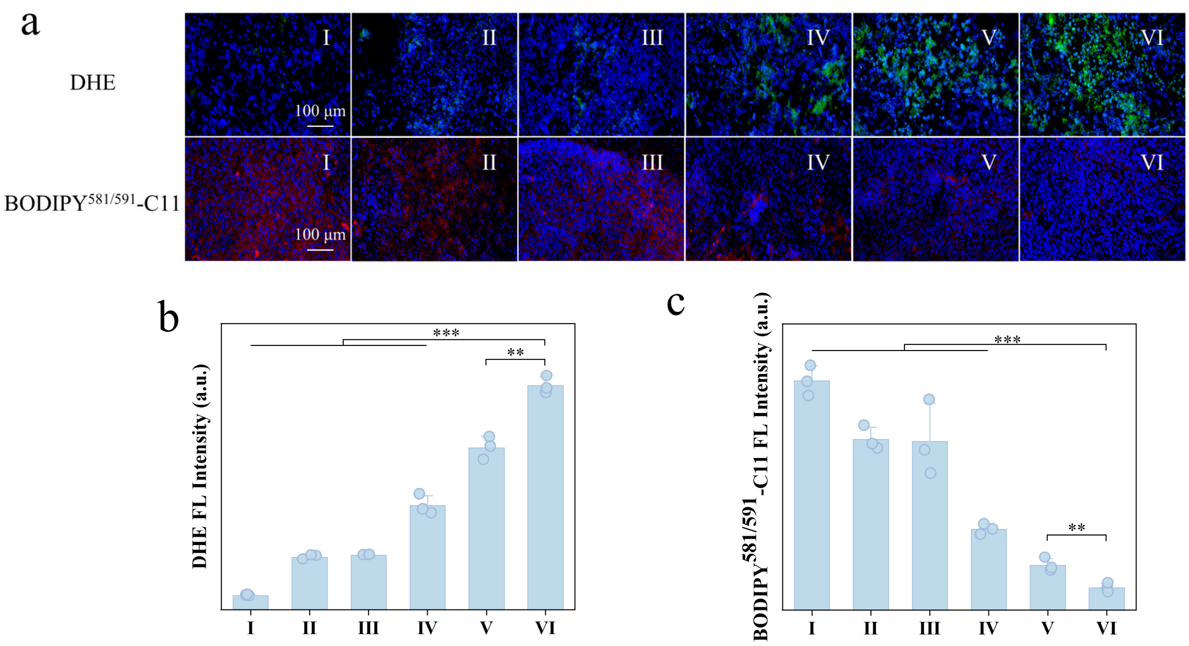

Figure S32. (a) Immunofluorescence images of CRT in tumors. (b) Fluorescence intensity of CRT immunofluorescence images measured by ImageJ ($n = 3$). Groups: (I) PBS, (II) SRF, (III) FeShik, (IV) SRF@FeShik, (V) SRF@FeShik-HA, (VI) SRF@FeShik-cGAMP/HA.

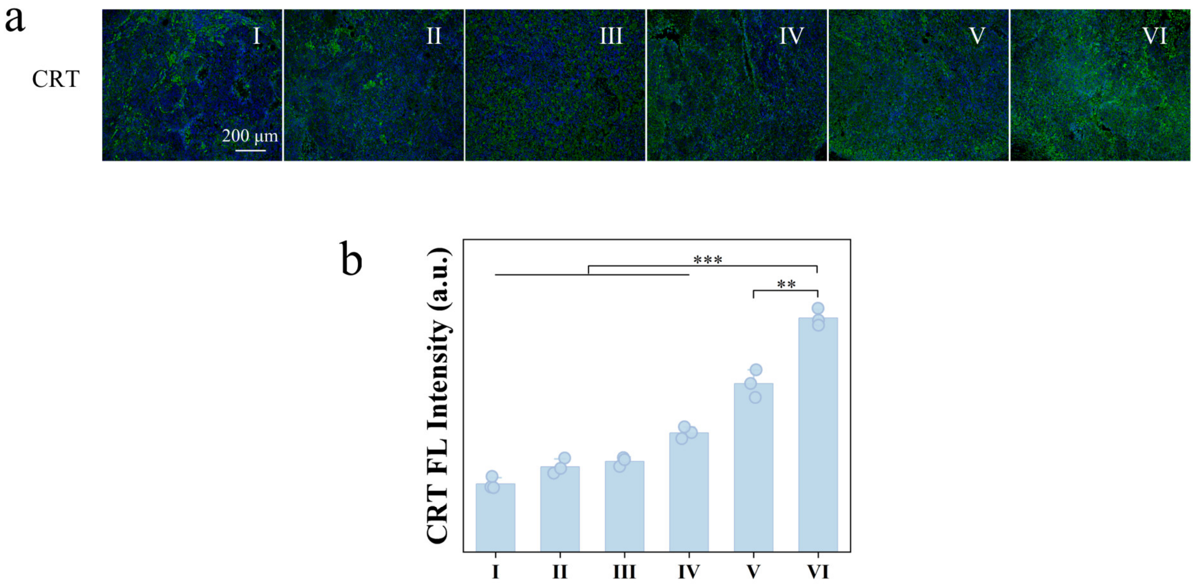

Figure S33. (a) Immunofluorescence images of HMGB1 in tumors. Groups: (I) PBS, (II) SRF, (III) FeShik, (IV) SRF@FeShik, (V) SRF@FeShik-HA, (VI) SRF@FeShik-cGAMP/HA.

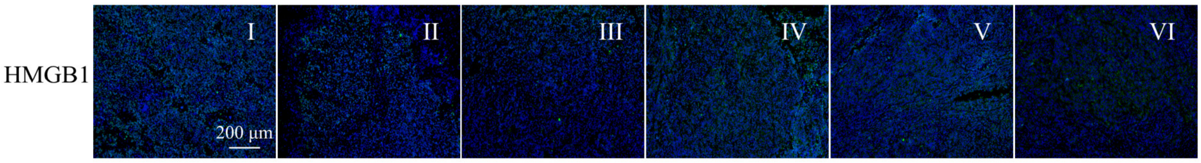

Figure S34. Immunofluorescence images of p-STING, p-TBK1, and p-IRF3 expression in tumors after different treatments. Groups: (I) PBS, (II) SRF, (III) FeShik, (IV) SRF@FeShik, (V) SRF@FeShik-HA, (VI) SRF@FeShik-cGAMP/HA.

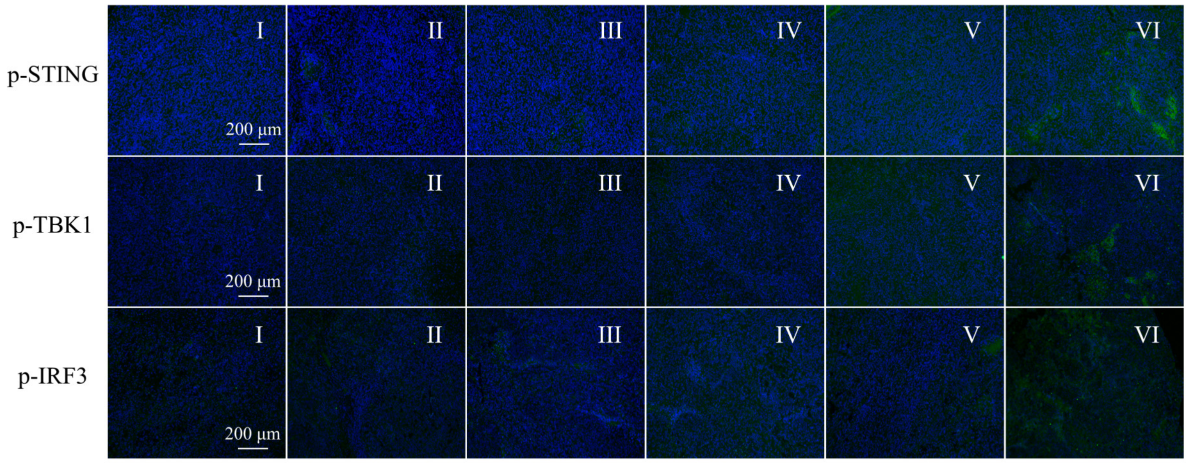

Figure S35. H&E staining of major organs after treatment with different treatments. Groups: (I) PBS, (II) SRF, (III) FeShik, (IV) SRF@FeShik, (V) SRF@FeShik-HA, (VI) SRF@FeShik-cGAMP/HA.

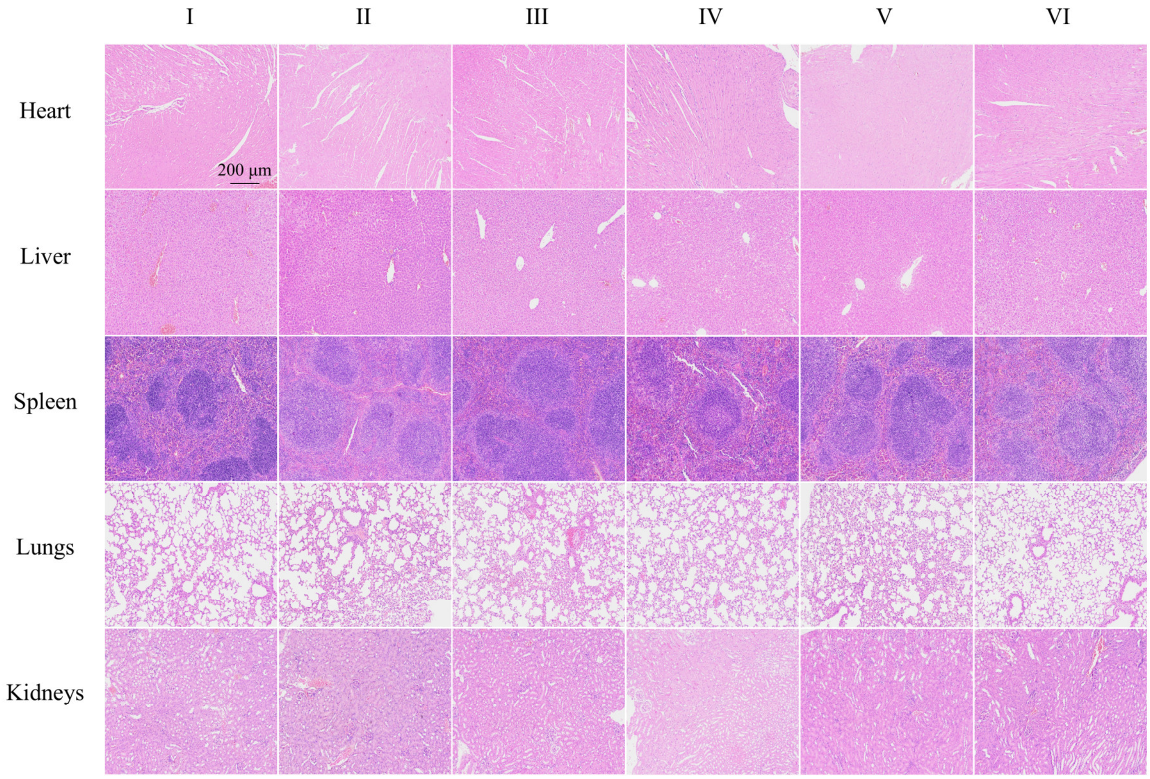

Figure S36. Quantitative analysis of TNF-α (a) and IL-6 (b) secretion levels in serum of Hepa1-6 tumor-bearing mice after different treatments ($n = 3$). Groups: (I) PBS, (II) SRF, (III) FeShik, (IV) SRF@FeShik, (V) SRF@FeShik-HA, (VI) SRF@FeShik-cGAMP/HA.

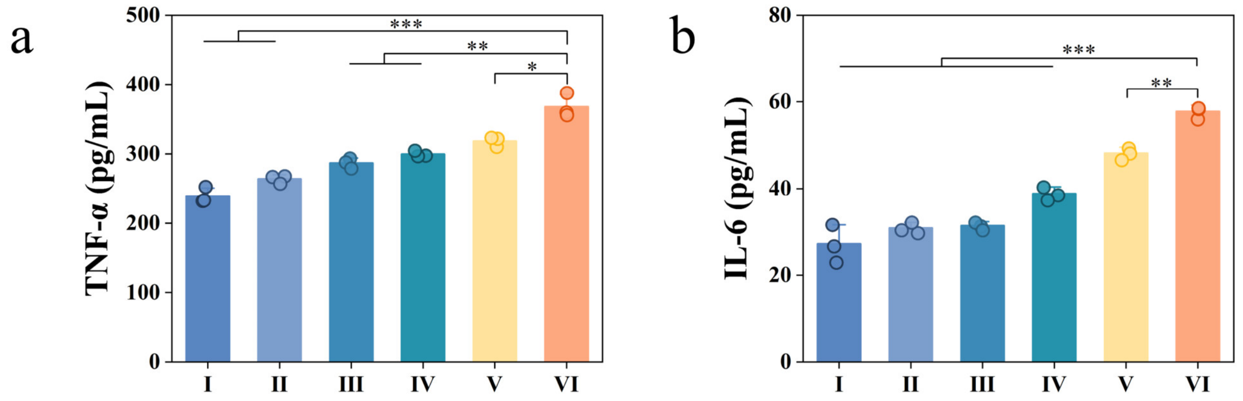

Figure S37. (a) Immunofluorescence images of $CD8^+$ T cells in tumors. (b) Fluorescence intensity of CD8 immunofluorescence images measured by ImageJ (n = 3). Groups: (I) PBS, (II) SRF, (III) FeShik, (IV) SRF@FeShik, (V) SRF@FeShik-HA, (VI) SRF@FeShik-cGAMP/HA.

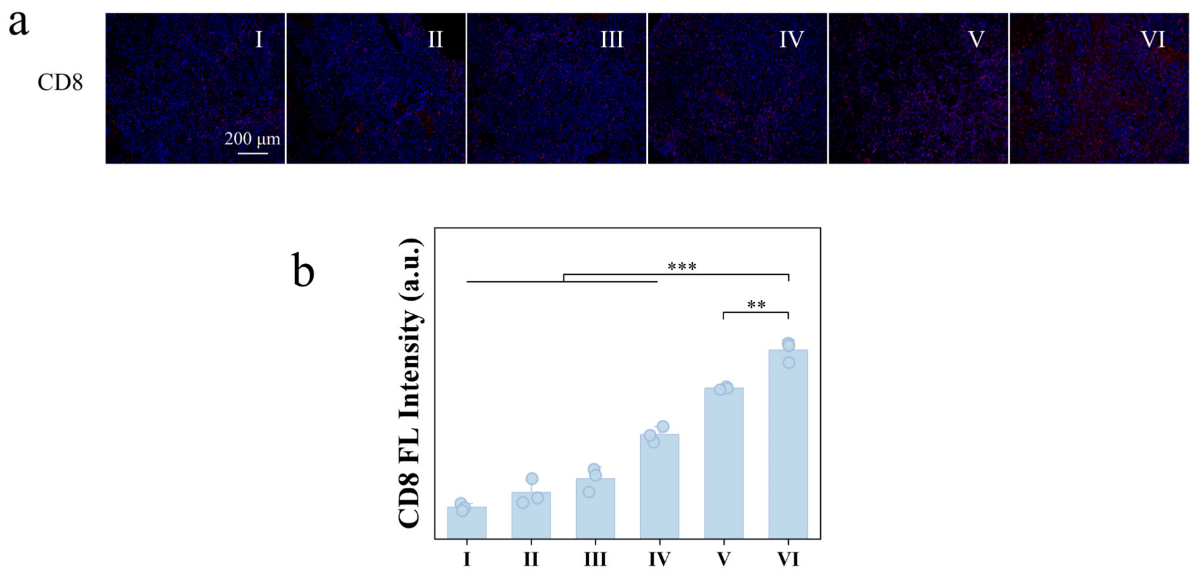

Figure S38. Quantitative analysis of IFN-γ secretion levels in serum of Hepa1-6 tumor-bearing mice after different treatments (*n* = 3). Groups: (I) PBS, (II) SRF, (III) FeShik, (IV) SRF@FeShik, (V) SRF@FeShik-HA, (VI) SRF@FeShik-cGAMP/HA.

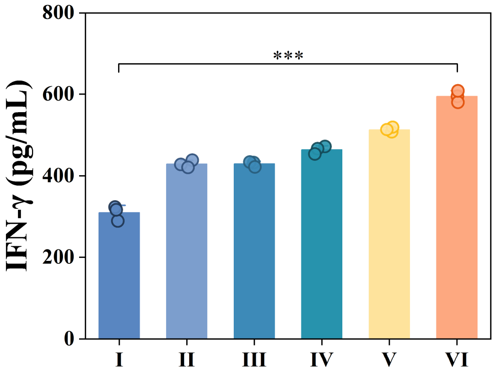

Figure S39. (a) Immunofluorescence images of CD86 and CD206 in tumors. Fluorescence intensity of CD86 (b) and CD206 (c) immunofluorescence images measured by ImageJ (n = 3). Groups: (I) PBS, (II) SRF, (III) FeShik, (IV) SRF@FeShik, (V) SRF@FeShik-HA, (VI) SRF@FeShik-cGAMP/HA.

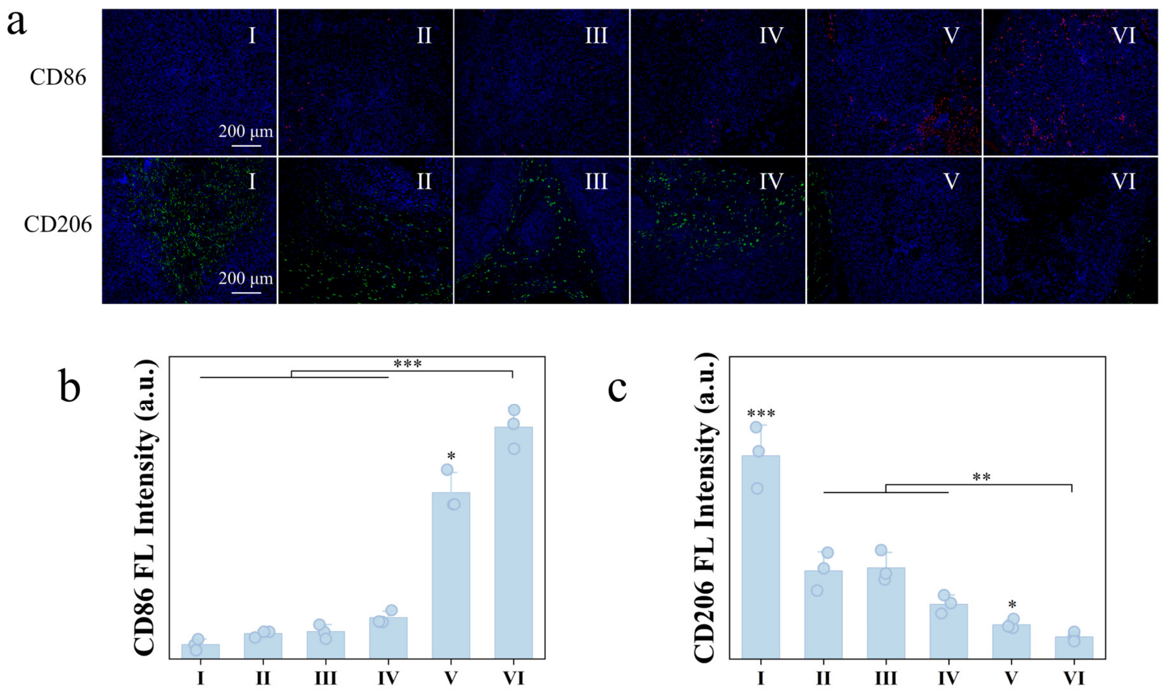

Figure S40. (a) Immunofluorescence images of Tregs in tumors. (b) Fluorescence intensity of FOXP3 immunofluorescence images measured by ImageJ (n = 3). Groups: (I) PBS, (II) SRF, (III) FeShik, (IV) SRF@FeShik, (V) SRF@FeShik-HA, (VI) SRF@FeShik-cGAMP/HA.

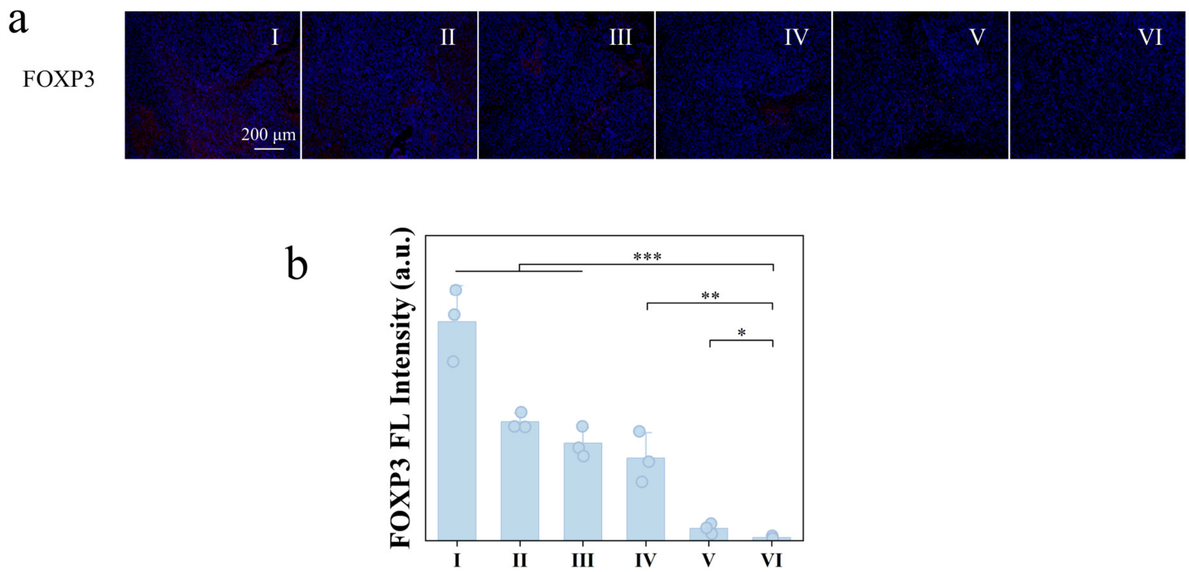

Figure S41. (a) Fluorescence images of DHE (pseudocolor: green) in major organs. (b) Fluorescence intensity of DHE fluorescence images measured by ImageJ (n = 3).

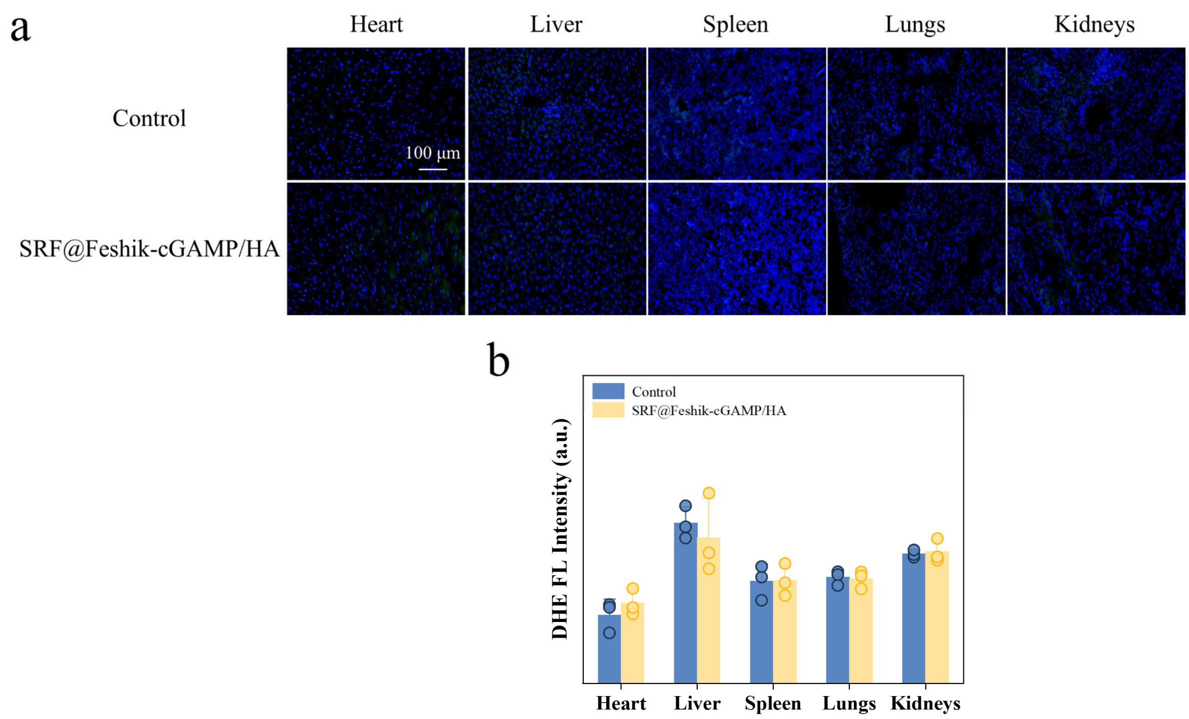

Figure S42. (a) Fluorescence images of BODIPY$^{581/591}$-C11 (red fluorescence: reduced state) in major organs. (b) Fluorescence intensity of BODIPY$^{581/591}$-C11 fluorescence images measured by ImageJ (n = 3).

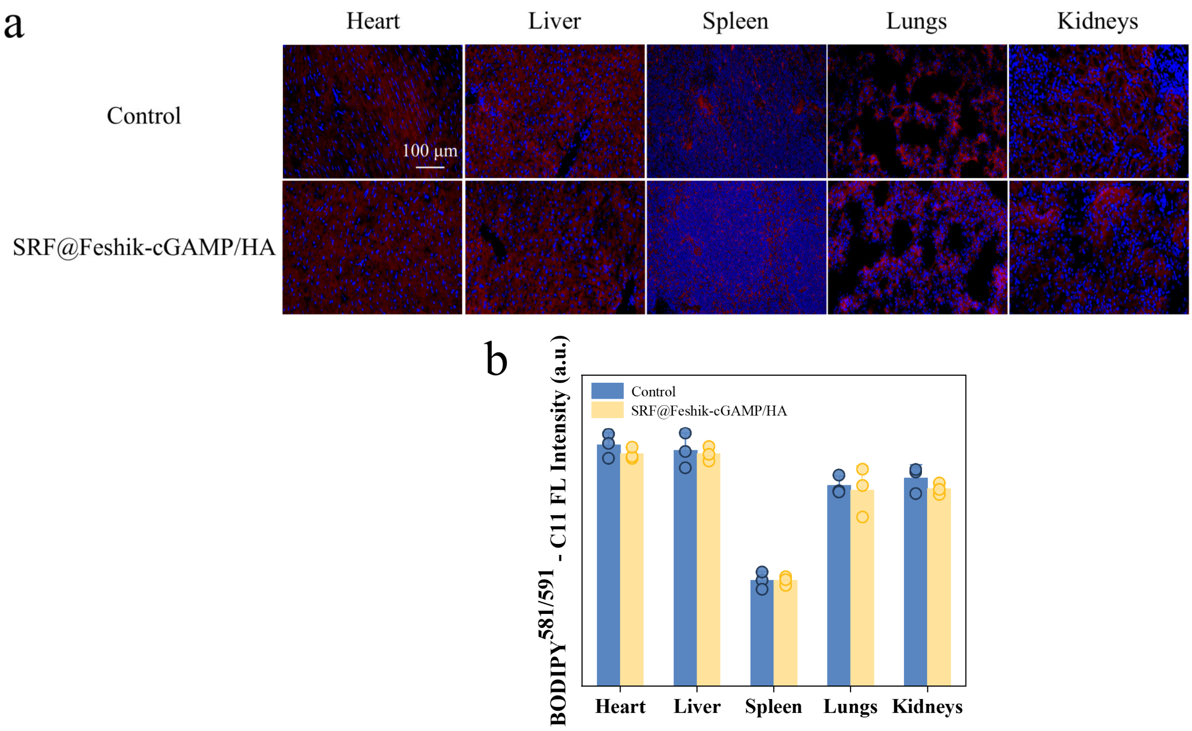

Supplement: Supplementary file 1 — Supplementary Material 1. [file 13046_2026_3726_MOESM1_ESM.pdf]
